# Matrix plasticity and the molecular basis of extracellular filament assembly in *Bacillus cereus*

**DOI:** 10.1126/sciadv.aea1826

**Published:** 2026-04-15

**Authors:** Ana Álvarez-Mena, Muhammed Bilal Abdul Shukkoor, Joaquín Caro-Astorga, Mélanie Berbon, María Luisa Antequera-Gómez, Montserrat Grifé-Ruiz, Axelle Grélard, Brice Kauffmann, Estelle Morvan, Oscar P. Kuipers, Antonio de Vicente, Birgit Habenstein, Antoine Loquet, Diego Romero

**Affiliations:** ^1^ Departamento de Microbiología, Instituto de Hortofruticultura Subtropical y Mediterránea La Mayora, Universidad de Málaga-Consejo Superior de Investigaciones Científicas, Universidad de Málaga, Málaga, Spain.; ^2^ Institut de Chimie et de Biologie des Membranes et des Nano-objets, Institut Européen de Chimie et Biologie, CNRS, UMR5248, Université de Bordeaux, Pessac, France.; ^3^ LSBU-London South Bank University, 103 Borough Road, London, SE1 0AA. UK.; ^4^ Institut Européen de Chimie et Biologie, UAR3033 CNRS, Université de Bordeaux, Inserm US01, Pessac F-33600, France.; ^5^ Department of Molecular Genetics, Groningen Biomolecular Sciences and Biotechnology Institute, Centre for Synthetic Biology, University of Groningen, Nijenborgh 7, 9747 AG, Groningen, Netherlands.; ^6^ Departamento de Microbiología, Universidad de Málaga, Bulevar Louis Pasteur 31 (Campus Universitario de Teatinos), Málaga 29071, Spain.; ^7^ Departamento de Microbiología, Facultad de Medicina, Universidad de Málaga, Bulevar Louis Pasteur 32 (Campus Universitario de Teatinos), Málaga 29071, Spain.

## Abstract

The controlled assembly of extracellular filaments is essential for bacterial multicellularity and surface colonization. While Gram-positive bacteria rely on a variety of mechanisms to construct surface-associated fibers, many noncanonical pathways remain largely unexplored. Here, we identify a regulated, sortase-independent system in *Bacillus cereus* that governs the polymerization of filaments within the extracellular matrix (ECM). This tripartite system comprises CapP, a chaperone-like protein, and the structural subunits TasA and CalY. CapP modulates filament formation in a concentration- and domain-dependent manner, promoting ordered heteropolymer assembly while preventing uncontrolled aggregation. Disrupting this pathway leads to distinct compensatory changes in matrix composition—including exopolysaccharide expression, extracellular DNA release, and flagellar regulation—revealing an unexpected level of matrix plasticity. Our findings uncover a unique mechanism of ECM biogenesis in Gram-positive bacteria and suggest that plasticity in matrix organization may be a widespread adaptive strategy across bacterial lineages.

## Teaser

A modular protein system drives filament assembly and reveals unexpected adaptability in the *Bacillus cereus* biofilm matrix.

## INTRODUCTION

The assembly of extracellular matrices (ECMs) is a defining feature of bacterial multicellularity, enabling surface colonization, collective behavior, and resilience in diverse environments. In Gram-positive bacteria, ECMs are composed of exopolysaccharides, extracellular DNA (eDNA), and structural proteins that form stable fibrillar networks. Among these, proteinaceous filaments are of particular interest due to their amyloid-like properties and essential roles in intercellular cohesion and biofilm integrity (*1*–*6*).

*Bacillus cereus* is a spore-forming, soil-dwelling bacterium and a common foodborne pathogen capable of colonizing the gastrointestinal tract of mammals and arthropods through contaminated produce and processed foods (*7*–*12*). Its ability to persist in both environmental and host-associated niches is closely linked to its capacity to form robust biofilms. Despite the ecological and clinical relevance of *B. cereus*, the molecular mechanisms governing its ECM assembly remain poorly understood. Insights from *Bacillus subtilis*, where ECM matrix formation has been extensively studied, provide a useful reference. In *B. subtilis*, TasA is the main protein component of the ECM, encoded within the *tapA*-*sipW-tasA* operon. TasA assembles into amyloid-like fibers that provide structural integrity, while TapA facilitates filament formation (*13*–*15*). SipW mediates protein secretion and has also been reported to influence expression of matrix-associated genes, highlighting a bifunctional role in both ECM processing and regulation (*16*). A SinR-SlrR regulatory switch tightly controls operon expression, coupling ECM production with the repression of motility during the transition to the biofilm state (*17*–*21*). During planktonic growth, SinR represses the *tapA*-*sipW*-*tasA* and *epsA-O* operons, whereas upon biofilm induction, SinI binds and inactivates SinR, leading to derepression of matrix genes and activation of SlrR-dependent pathways (*22*–*25*).

In contrast, *B. cereus* conserves the SinI/SinR antirepressor-repressor pair that governs the switch between biofilm formation and swimming motility but lacks *slrR* (*26*, *27*). Instead, it encodes the pleiotropic regulator PlcR, which coordinates biofilm development with virulence control (*28*, *29*). It also lacks *tapA*; however, *sipW* and one *tasA* homolog form a single operon, while a second *tasA* homolog, *calY*, is encoded in a separate operon within the same genomic region (*30*). Preliminary studies indicate that TasA and CalY mediate distinct adhesive roles, with TasA contributing mainly to abiotic surface attachment and CalY promoting intercellular cohesion (*30*). Moreover, CalY exhibits a dual functionality, acting as surface adhesin during early stationary phase and assembling into fibers during biofilm maturation (*31*). In addition, a previously uncharacterized gene, *bc1280*, located within this biofilm-active locus and regulated by *SinR*, has been associated with ECM development (*30*, *32*). This organization reflects a divergent regulatory and structural architecture of ECM assembly in *B. cereus* compared to *B. subtilis.*

Here, we identify BC1280, renamed CapP, as a critical chaperone-like factor that orchestrates the assembly of TasA-CalY heterofilaments in *B. cereus*. Using genetic, biochemical, and structural approaches, we reveal that CapP modulates filament polymerization in a concentration- and domain-dependent manner, establishing a tripartite system that regulates ECM architecture. Beyond filament assembly, we show that each component influences the transcriptional program and the composition of matrix constituents, suggesting that ECM biogenesis is governed by an integrated, dynamically regulated mechanism. Notably, genetic perturbations in this system trigger compensatory responses—including eDNA overproduction and flagellar activation—that remodel matrix structure and composition, underscoring the inherent plasticity of the *B. cereus* ECM.

Our findings uncover a previously unknown, modular strategy for ECM formation in Gram-positive bacteria and suggest that such chaperone-mediated systems may extend beyond the canonical pili and amyloid-like assembly pathways described to date. Moreover, they highlight matrix versatility as a potentially widespread and adaptive feature of bacterial biofilms.

## RESULTS

### TasA and CalY differentially influence matrix structure and gene expression

TasA and CalY share 61% amino acid identity, supporting the hypothesis that *calY* arose from *tasA* via gene duplication, a process often associated with the evolution of moonlighting proteins with complementary functions (*33*). Building on previous studies that suggested nonredundant roles for these proteins (*30*), we examined the phenotypes for Δ*tasA* and Δ*calY* mutants using crystal violet staining, which reflects the total surface-adhered biofilm material, including both cells and ECM components. As previously reported, the Δ*tasA* mutant formed a thick biofilm ring that detached after 72 hours, whereas the Δ*calY* mutant exhibited reduced biomass accumulation compared to the wild-type strain at 48 and 72 hours (fig. S1, A and B, and table S1). These observations prompted us to further examine how TasA and CalY differentially contribute to matrix architecture.

**Fig. 1.**
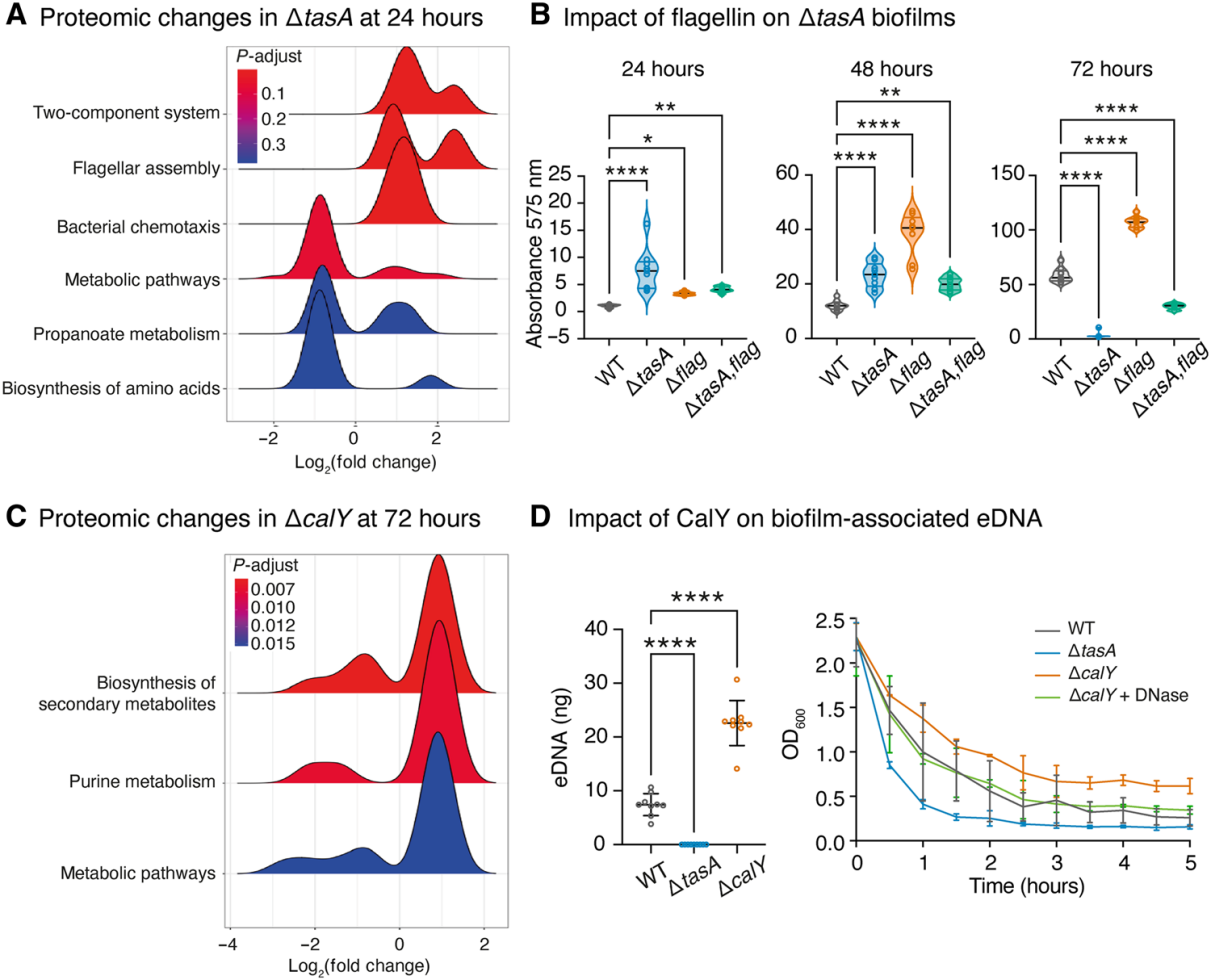
Deletion of *tasA* or *calY* triggers distinct genetic and physiological responses. (**A**) Ridgeplot representation of Kyoto Encyclopedia of Genes and Genomes (KEGG) pathways significantly affected in the Δ*tasA* strain compared to the wild-type at 24 hours, as identified by iTRAQ analysis. (**B**) Biofilm formation by wild-type (WT), Δ*tasA*, Δ*flag*, and Δ*tasA,flag* strains after 72 hours of static growth in TY medium at 28°C. Surface-adhered biomass was quantified by crystal violet staining and absorbance measurement at 575 nm. Data represent three independent experiments, each with three technical replicates. Statistical analysis was performed at each time point using ordinary one-way analysis of variance (ANOVA) with Dunnett’s multiple comparisons test against the wild-type control. At 24 hours, significance levels were *P = 0.0424, **P = 0.005, and ****P < 0.0001. At 48 hours, significance values were **P = 0.0031 and ****P < 0.0001. At 72 hours, all comparisons showed ****P < 0.0001. (**C**) KEGG pathway analysis of proteins differentially affected in Δ*calY* biofilm cells at 72 hours, as identified by iTRAQ analysis. (**D**) Left: Quantification of eDNA extracted from biofilms grown for 72 hours in TY medium. eDNA levels were normalized to the initial biomass optical density. Data represent at least nine biological replicates per strain. Statistical significance was determined using one-way ANOVA followed by Dunnett’s multiple comparisons test against the wild-type control (****P < 0.0001). Right: Autoaggregation kinetics of cell suspensions monitored by optical density at 600 nm. Data represent mean ± SD from three biological replicates. DNase, deoxyribonuclease.

To determine whether the observed biomass differences reflected altered cellular organization, we compared the cell densities of each strain in planktonic and biofilm-associated fractions over time. While planktonic growth was similar, biofilm-associated Δ*tasA* cultures showed elevated cell density at 24 hours, which normalized by 72 hours (fig. S1, C and D, and tables S2 and S3). In contrast, Δ*calY* followed wild-type dynamics early on but diverged later, with reductions in both biomass and live cell counts at 48 hours, and a pronounced decrease at 72 hours, with a ~40% reduction in biomass and an approximately 10-fold decrease in live cell counts (fig. S1, C and D, and tables S2 and S3). These differences pointed to potential shifts in ECM-related gene expression or matrix composition in the absence of each protein. We first examined the Δ*tasA* mutant, in which early biomass accumulation could reflect compensatory activation of ECM pathways. However, transcriptomic analysis at 24 hours revealed modest changes (39 up-regulated and 27 down-regulated genes), with no direct involvement in ECM biosynthesis or adhesion (fig. S1E and table S4). In contrast, proteomic analysis using iTRAQ (isobaric tags for relative and absolute quantification) identified 211 differentially accumulated proteins (Fig. 1A and table S5), with strong enrichment in components of the flagellar system, including chemotaxis proteins (CheA and CheV), motor elements (FliM and MotA), and structural proteins (FlgE, FlgK, and FliC). These observations are consistent with the TasA-linked shifts toward motility pathways reported in *B. subtilis* (*34*). In *B. cereus*, however, the signature is more evident at the protein level (iTRAQ) than in the 24-hour transcript levels, pointing to posttranscriptional regulation and species-specific control within the matrix assembly program. Given the established role of the flagellar system in recruiting planktonic cells during early biofilm formation (*35*), we hypothesized that elevated flagellin levels may contribute to the Δ*tasA* phenotype, characterized by enhanced cell recruitment and increased biofilm biomass. To test this, we deleted flagellin subunit genes in the Δ*tasA* background. Biomass quantification revealed that Δ*flag* cells formed denser biofilms than the wild type at 72 hours, while Δ*tasA,flag* double mutant partially rescued the Δ*tasA* phenotype (Fig. 1B and table S6). Estimation of biofilm-associated cell density supported this trend, as both Δ*tasA* and Δ*flag* mutants showed elevated cell counts at 24 hours, whereas the Δ*tasA,flag* strain exhibited a marked reduction (fig. S1F). By 72 hours, however, both Δ*tasA,flag* and Δ*flag* mutants exceeded wild-type levels (fig. S1F), suggesting that flagellar overexpression in Δ*tasA* may facilitate early biomass accumulation. In parallel, iTRAQ data (Fig. 1A and table S5) revealed reduced levels of proteins linked to sporulation, antimicrobial resistance, and purine metabolism in Δ*tasA*, including PurC, PurS, and PurM. These shifts indicate broader metabolic rewiring beyond flagellar induction.

We next examined Δ*calY*, which showed minimal transcriptional changes at 24 hours (fig. S1G and table S7) but substantial transcriptomic remodeling at 72 hours (161 genes up-regulated, 66 down-regulated; fig. S1H and table S8). Proteomic profiling corroborated these changes (Fig. 1C and table S9), particularly in the purine biosynthesis pathway, where multiple genes and proteins (e.g., *bc0323* and *bc0331*) were overexpressed. This pattern, combined with the up-regulation of genes involved in nucleoside and formate synthesis (e.g., *bc0640* and *bc2383*), suggested a metabolic shift toward DNA biosynthesis in the absence of *calY*. Given the established role of eDNA in *B. cereus* ECM (*36*), and the involvement of these purine metabolism genes in DNA synthesis previously linked to eDNA production, we hypothesized that increased purine metabolism in Δ*calY* may contribute to eDNA accumulation. Quantification of eDNA supported this, showing that Δ*calY* produced significantly more eDNA than wild type, while Δ*tasA* showed no detectable eDNA despite continued CalY expression (Fig. 1D). Autoaggregation assays indicated a possible role for eDNA in cell-cell adhesion, as Δ*calY* showed delayed sedimentation that was partially restored by deoxyribonuclease treatment, whereas Δ*tasA* cells sedimented faster than wild type (Fig. 1D). Further analysis of the Δ*calY* transcriptome at 72 hours (table S8) revealed the down-regulation of genes involved in cell wall degradation and up-regulated enzymes associated with peptidoglycan remodeling (*bc3441* and *bc5027*), as well as adhesion factors (*bc5056*), supporting a broader remodeling of the cell envelope in the absence of CalY.

Collectively, these data indicate that TasA and CalY influence distinct but complementary matrix-related pathways, with TasA loss activating motility and repressing eDNA, while CalY loss triggers eDNA production and cell envelope remodeling. These divergent responses suggest compensatory strategies for maintaining the ECM structure in the absence of each filament-forming protein.

### TasA and CalY coassemble into heterofilaments through direct interaction

**Fig. 2.**
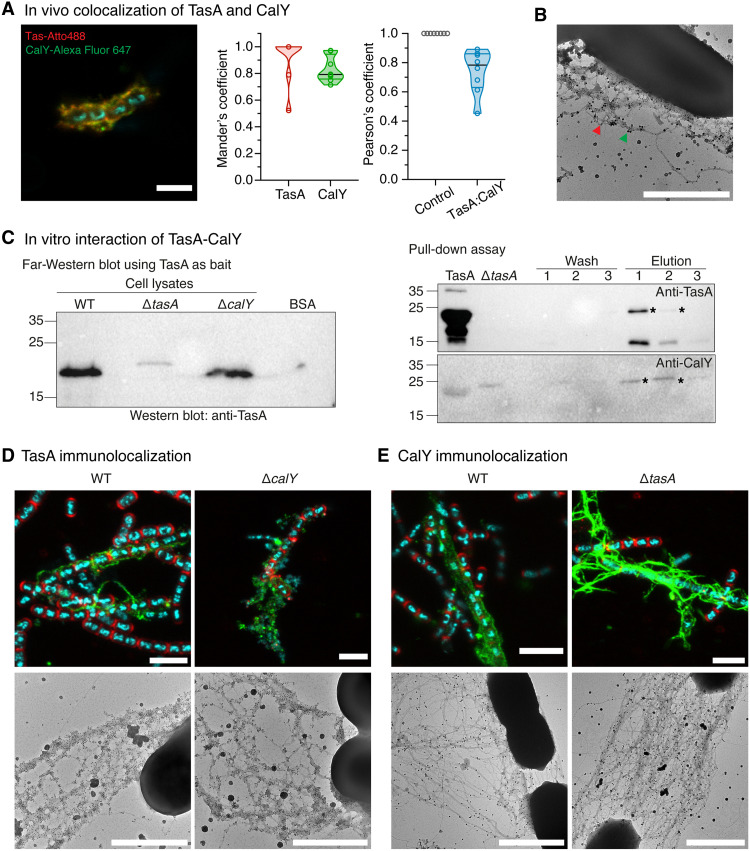
TasA and CalY form heteropolymeric filaments. (**A**) CLSM images showing TasA and CalY localization by immunocytochemistry using specific primary antibodies. TasA and CalY were detected with secondary antibodies conjugated to Atto 488 and Alexa Fluor 647, respectively. The merged image shown here corresponds to the same field of view as the single-channel images presented in fig. S1I. Colocalization was quantified in ImageJ using the colocalization threshold tool across at least six fields of view per sample from three independent experiments. Mander’s overlap coefficients and Pearson correlation values are shown as violin plots, with all individual measurements displayed. As a negative control, Pearson’s coefficient was calculated by overlapping the CalY–Alexa 647 channel with itself, which, as expected for perfect colocalization, yielded a value close to 1. In contrast, the TasA-CalY colocalization averaged 0.74 ± 0.14 across all fields. Scale bar, 4 μm. (**B**) TEM images showing TasA and CalY localization using specific primary antibodies. TasA and CalY were detected with secondary antibodies coupled to 10-nm and 20-nm nanogold particles, respectively. Images were collected from two independent experiments, with three to five fields per condition. Scale bar, 1 μm. (**C**) Left: Far-Western blot using purified TasA (produced by heterologous expression in *E. coli*) as bait and cell lysates from wild-type, Δ*tasA*, and Δ*calY* strains. Proteins were renatured and probed with anti-TasA antibodies. Bovine serum albumin (BSA) was used as a negative control. The expected molecular weight of both TasA and CalY is ~21.8 kDa, and the detected immunoreactive bands correspond to this size. Right: Pull-down assay using purified TasA (heterologously produced in *E. coli*) as bait and Δ*tasA* cell lysate as prey. Lanes correspond to input TasA, input Δ*tasA* cell lysate, wash fractions obtained using 50 mM imidazole, and elution fractions obtained using 500 mM imidazole. Western blots were sequentially detected with anti-TasA and anti-CalY antibodies. Asterisks indicate the TasA and CalY bands at ~21.8 kDa that coelute in the 500 mM imidazole fraction, consistent with their interaction. Experiments were performed twice independently with consistent results. Minor cross-reactivity of the anti-CalY antibody with purified TasA is observed due to high sequence identity. (**D**) Immunolocalization of TasA in the WT and *calY* mutant. CLSM images show TasA labeled in green (Atto 488), the cell wall in red (WGA), and DNA in blue (Hoechst). CLSM images were collected from three independent experiments, analyzing at least six fields per sample. Electron microscopy (EM) micrographs display immunogold labeling using 10-nm nanogold for TasA, collected from two independent experiments with three to five representative fields per condition. Scale bars, 5 μm (CLSM) and 1 μm (TEM). (**E**) Immunolocalization of CalY in the WT and *tasA* mutant. CLSM images show CalY labeled in green (Atto 488), the cell wall in red (WGA), and DNA in blue (Hoescht). CLSM images were collected from three independent experiments, analyzing at least six fields per sample. EM micrographs display immunogold labeling using 20-nm nanogold for CalY, collected from two independent experiments with three to five representative fields per condition. Scale bars, 7 μm (WT strain CLSM), 5 μm (Δ*tasA* CLSM), and 1 μm (TEM).

To determine whether the distinct phenotypes of Δ*tasA* and Δ*calY* strains arise from differences in supramolecular organization, we investigated the structural relationship between TasA and CalY. Prior work indicated that CalY is secreted into the ECM during biofilm development and may promote TasA polymerization in vitro by intercalating into filamentous structures without disrupting their architecture (*37*). These findings raise the possibility that TasA and CalY assemble into heteropolymeric filaments in vivo. We first assessed their spatial colocalization using immunocytochemistry and confocal laser scanning microscopy (CLSM). Biofilms were stained with antibodies targeting TasA and CalY, labeled with Atto 488 and Alexa Fluor 647, respectively. In wild-type cells, TasA and CalY showed substantial overlap within the ECM, with a Pearson’s correlation coefficient of 0.74 ± 0.14, consistent with coassembly into shared supramolecular structures (Fig. 2A and fig. S1I). Transmission electron microscopy (TEM) further confirmed the colocalization, showing filaments decorated with both anti-TasA and anti-CalY nanogold particles (Fig. 2B).

To evaluate whether this colocalization reflects a direct physical interaction, we performed a far-Western blot using recombinant TasA, produced in *Escherichia coli*, as a probe. When incubated with cell lysates from wild-type, Δ*tasA*, and Δ*calY* strains, TasA bound specifically to components present in the Δ*tasA* and wild-type lysates but not to bovine serum albumin (BSA) controls, suggesting interaction with native CalY (Fig. 2C). We confirmed this interaction by pull-down assay: His-tagged TasA immobilized on nickel–nitrilotriacetic acid (Ni-NTA) resin was incubated with Δ*tasA* lysate, and eluted fractions were analyzed by immunoblotting with an anti-CalY antibody, which revealed the copurification of CalY alongside TasA, indicating a stable complex between the two proteins (Fig. 2C).

Having established a direct interaction, we next examined whether TasA and CalY affect each other’s spatial distribution. CLSM analysis revealed that TasA filaments localize at the cell surface in wild-type cells, but in Δ*calY* mutants, TasA appears as scattered foci throughout the ECM (Fig. 2D). Some of these TasA foci coincided with extracellular Hoechst-stained regions, consistent with the previously noted increase in eDNA in Δ*calY* (Fig. 1D). TEM confirmed the presence of anti-TasA nanogold particles along filaments in both wild-type and Δ*calY* biofilms, indicating that TasA filament formation occurs even when its spatial organization is altered (Fig. 2D). Conversely, CLSM analysis revealed that CalY filaments are present in both wild-type and Δ*tasA* strains, although Δ*tasA* cells exhibited a more extensive and structured CalY network (Fig. 2E). TEM corroborated these observations, showing ECM filaments decorated with anti-CalY nanogold particles in both strains, with Δ*tasA* biofilms displaying a denser CalY filament scaffold (Fig. 2E).

Together, these findings support a model in which TasA and CalY copolymerize into surface-associated heterofilaments and mutually influence each other’s structural distribution. In the absence of one component, the remaining paralog undergoes compensatory spatial reorganization, potentially to maintain ECM function under altered matrix conditions.

### CapP is required for ECM maturation and biofilm development across *B. cereus* strains

**Fig. 3.**
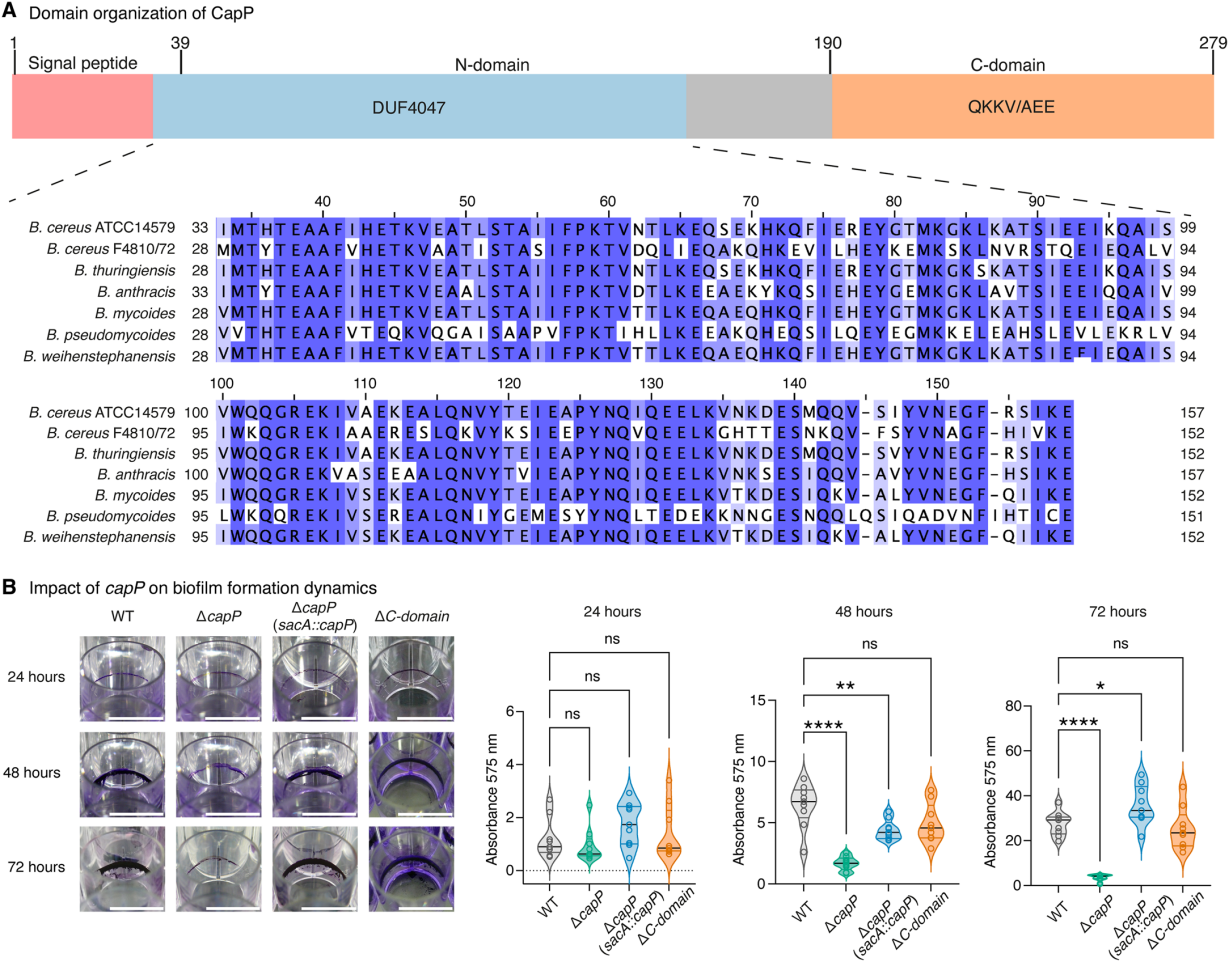
CapP is essential for biofilm development in *B. cereus*. (**A**) Schematic representation of CapP domain architecture. The signal peptide (M^1^-A^39^) is shown in pink, the DUF4047 domain (I^33^-E^157^) located within the N-domain is highlighted in blue, and the C-domain containing the repetitive region (Q^190^-E^279^) is shown in orange. Amino acid alignment of DUF4047 among *B. cereus* group strains indicates high conservation; residues are color-coded using Jalview’s (*86*) default Percentage Identity scheme, where higher identity values are represented by darker tones and lower identity by progressively lighter tones: >80% identity in the darkest tone, >60% in an intermediate tone, >40% in a pale tone, and ≤40% in white. (**B**) Biofilm formation was evaluated for WT, Δ*capP*, Δ*capP* (*sacA*::*capP*) (Δ*capP* complemented by chromosomal integration of *capP* at the *sacA* neutral locus under its native promoter), and Δ*C-domain* strains using crystal violet staining at different time points. Scale bars, 1 cm. Adhered biofilm biomass was quantified by measuring absorbance at 595 nm. Violin plots show all individual measurements from three independent experiments, each with three technical replicates. Statistical analysis was performed using ordinary one-way ANOVA with Dunnett’s multiple comparison test versus WT; symbols on the plots indicate significance: ns, not significant; *P < 0.05, **P < 0.01, and ****P < 0.0001.

While TasA and CalY function as structural components of the ECM, the genomic region encoding these proteins also contains an uncharacterized gene, *bc1280*, whose potential role in ECM organization remains unknown. We next explored the contribution of this gene product—hereafter referred to as CapP (CalY-assisted polymerization protein) to biofilm development. Phylogenetic analysis revealed that CapP is conserved within the *B. cereus* group but absent from *B. subtilis* (fig. S2A). The CapP sequence includes three regions: (i) a predicted N-terminal signal peptide (residues 1 to 39); (ii) an N-terminal domain (residues 40 to 189) containing a conserved DUF4047 motif; and (iii) a C-terminal domain [(C-domain); residues 190 to end] composed of strain-variable QKKV/AEE tandem repeats (Fig. 3A and fig. S2, B and C).

Previous transcriptomic analyses showed that *capP* is specifically expressed in biofilm-associated cells and is part of the SinR regulon (*27*, *32*). We confirmed this pattern using reverse transcription quantitative polymerase chain reaction (RT-qPCR) and found that *capP* is transcribed independently of the adjacent *sipW-tasA* and *calY* operons (fig. S2, D and E). Notably, deletion of *capP* (Δ*capP*) in *B. cereus* ATCC14579 strongly impaired biofilm formation, resulting in a more severe phenotype than that of Δ*tasA* or Δ*calY* strains (Fig. 3B and table S10). This developmental arrest was also observed in strain *B. cereus* F4810/72, an emetic strain belonging to a separate phylogenetic group relative to *B. cereus* ATCC14579, in which *capP* deletion abolished pellicle formation (fig. S2F). Biofilm formation was fully restored at 72 hours upon complementation of Δ*capP* by chromosomal integration of *capP* at the *sacA* neutral locus under its native promoter (Fig. 3B and table S10), confirming that the observed defect was directly attributable to the gene loss. These results also suggest that the *capP* promoter is located in the noncoding region between *tasA* and *capP*.

Given the repetitive nature of the CapP C-domain, a feature often associated with structural stability (*38*), we tested whether this region was required for function. Notably, a truncated version lacking residues Q191 to E279 (Δ*C-domain*) restored biofilm formation to wild-type levels, as confirmed by biomass quantification using crystal violet staining (Fig. 3B). No significant differences were observed at 24 hours, when surface attachment is predominant, but biomass of the Δ*capP* strain diverged from that of the wild type at 48 hours, coinciding with ECM maturation (Fig. 3B and table S10). To assess the role of CapP in cell-to-cell cohesion, we performed autoaggregation assays. Wild-type and Δ*C-domain* strains showed rapid sedimentation, whereas Δ*capP* sedimented more slowly after 24 hours (fig. S2G). These data indicate that CapP is required for the transition from initial surface adhesion to mature ECM assembly, a process that appears to rely on its N-terminal domain.

### CapP function is posttranscriptional and independent of TasA/CalY expression

**Fig. 4.**
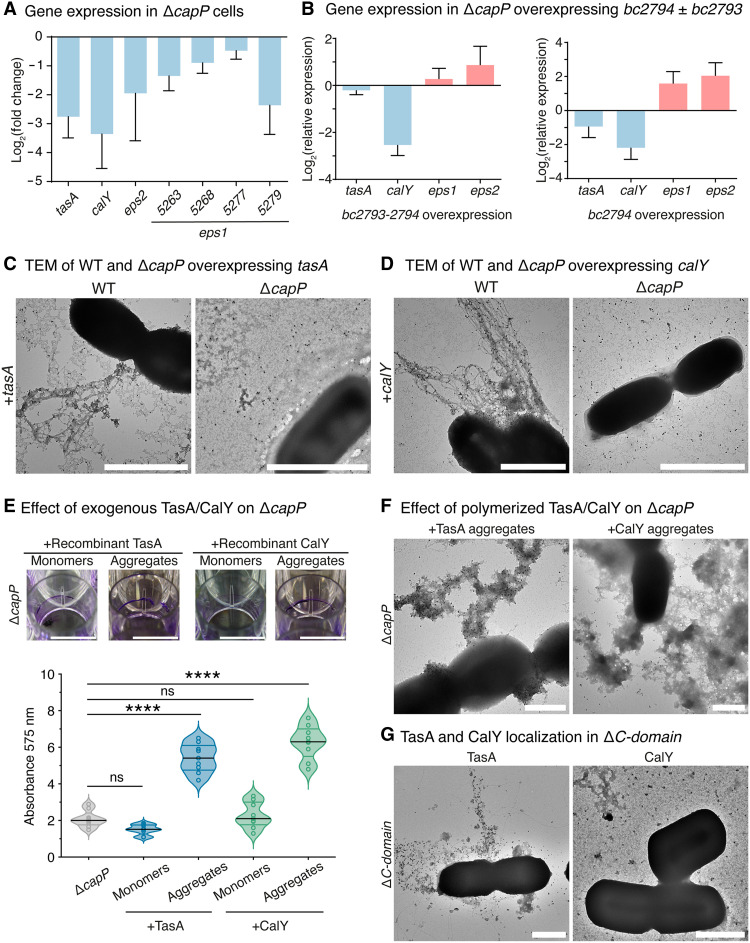
The biofilm-defective phenotype of Δ*capP* is independent of the expression level of *tasA* or *calY*. (**A**) Relative expression levels of *tasA*, *calY*, *eps2*, and genes from the *eps1* operon (*bc5263*, *bc5268*, *bc5277*, and *bc5279*) in Δ*capP* planktonic cells compared to the wild-type at 48 hours, as determined by RT-qPCR. Data are presented as mean ± SD from three biological replicates. (**B**) Relative expression levels of *tasA*, *calY*, *eps1*, and *eps2* in planktonic cells at 72 hours in the Δ*capP* strain overexpressing *bc2793-bc2794* or *bc2794*, compared to the wild-type strain. Data are presented as mean ± SD from three biological replicates. (**C** and **D**) Transmission electron micrographs of WT and Δ*capP* strains overexpressing *tasA* or *calY*, immunolabeled with specific anti-TasA or anti-CalY antibodies, respectively. Images were collected from two independent experiments, with three to five representative fields per condition. Scale bars, 2 μm. (**E**) Extracellular complementation of Δ*capP* by addition of 6 μM TasA or CalY (monomeric or polymerized). Scale bars, 1 cm. Biofilm biomass was quantified by crystal violet staining and absorbance at 575 nm. Data represent three biological replicates, each including three technical replicates. Statistical analysis was performed using one-way ANOVA followed by Dunnett’s multiple comparisons test, using the Δ*capP* mutant as the control. Statistical significance is indicated as: ****P < 0.0001. (**F**) Phenotype reversion of Δ*capP* upon addition of 6 μM polymerized TasA or CalY, visualized by immunolabeling with anti-TasA and anti-CalY antibodies. Images were collected from two independent experiments, with three to five representative fields per condition. Scale bars, 1 μm. (**G**) Negative-stained micrographs of TasA and CalY immunolabeling in the Δ*C-domain* strain. Images were collected from two independent experiments, with three to five representative fields per condition. Scale bars, 1 μm.

The severe biofilm-deficient phenotype of the Δ*capP* strain could arise from two nonmutually exclusive causes: impaired expression of ECM components or failure in filament polymerization. To explore this, we first performed transcriptomic analysis at 24 and 48 hours. While modest changes were observed at 24 hours (35 up-regulated, 4 down-regulated genes), more substantial shifts appeared at 48 hours, including strong repression of *tasA* and *calY* [log_2_ fold change (FC) ≈ −3.1 for both] (tables S11 and S12). RT-qPCR confirmed reduced expression of these genes as well as genes within the *eps1* and *eps2* exopolysaccharide loci (Fig. 4A). Among the down-regulated genes, we identified *bc2793* and *bc2794*, encoding a putative Clp protease and an extracytoplasmic function (ECF) sigma factor, respectively. Given the known role of ECF sigma factors in coordinating envelope stress responses (*39*, *40*), we hypothesized that this module might contribute to ECM regulation. Overexpression of *bc2793-2794*, or *bc2794* alone, in Δ*capP* cells significantly enhanced biofilm formation upon isopropyl-β-d-thiogalactopyranoside (IPTG) induction (fig. S3A and table S13). However, deletion of *bc2794* had little effect, and deletion of the operon produced only mild reduction in biofilm mass substantially less severe than in Δ*capP* (fig. S3A and table S14). These data suggest that this regulatory module promotes ECM formation but is insufficient to compensate for the loss of CapP.

Consistent with this, RT-qPCR revealed that overexpression of *bc2793-2794* restored *eps1* and *eps2* expression but not *tasA* or *calY* (Fig. 4B), reinforcing the idea that CapP is required for ECM protein functionality rather than their transcriptional regulation. We next asked whether exogenous overexpression of *tasA*, *calY*, or both could rescue the Δ*capP* phenotype. None of these constructs restored biofilm formation (fig. S3, B and C, and table S15), suggesting that CapP does not act through transcriptional activation of *tasA* and *calY*. TEM images of Δ*capP* cells overexpressing *tasA* showed anti-TasA labeling dispersed throughout the ECM, with no structured filaments observed (Fig. 4C and fig. S3D). Likewise, overexpression of *calY* alone led to a dispersed signal and the absence of detectable filaments (Fig. 4D). To directly test the role of CapP in polymerization, we added in vitro preassembled TasA and CalY filaments to Δ*capP* cultures. This partially restored the wild-type ring biomass phenotype (Fig. 4E, fig. S3E, and table S16). In contrast, the monomeric conformation of TasA or CalY failed to rescue biofilm formation. Furthermore, TEM analysis confirmed the presence of immunoreactive TasA and CalY aggregates on cell surfaces (Fig. 4F), indicating that preformed filaments can at least partially bypass the need for CapP in vivo. To dissect domain-specific functions, we examined filament formation in the Δ*C-domain* strain. TEM and immunogold labeling revealed TasA filaments with altered morphology, and CalY was observed as monomers or oligomers, but not filaments (Fig. 4G). These data suggest that the C-domain of CapP may facilitate proper polymerization and/or surface anchoring of TasA and CalY, predominantly targeting CalY.

Collectively, these findings may support a model in which CapP acts as a chaperone-like mediator of TasA-CalY polymerization. Its absence results in filament misassembly and impaired ECM formation, a phenotype only partially rescued by exogenous filaments. Further investigation is required to determine whether the down-regulation of *tasA* and *calY* observed at later time points reflects a feedback response to blocked secretion and aggregation in the cell envelope/extracellular milieu or cytoplasm.

### CapP localizes to the cell wall and associates with CalY in the ECM

To determine the subcellular localization of CapP and its spatial relationship with ECM components, we performed a combination of cell fractionation, immunodetection, and imaging assays. Because CapP is expressed at low levels and was not detectable by direct immunocytochemistry, we used a Δ*capP* strain containing a plasmid overexpressing a C-terminally His-tagged version of the protein. This construct fully rescued biofilm formation both with and without IPTG induction (fig. S4, A and B, and tables S17 and S18), likely due to basal promoter activity, confirming its functionality.

**Fig. 5.**
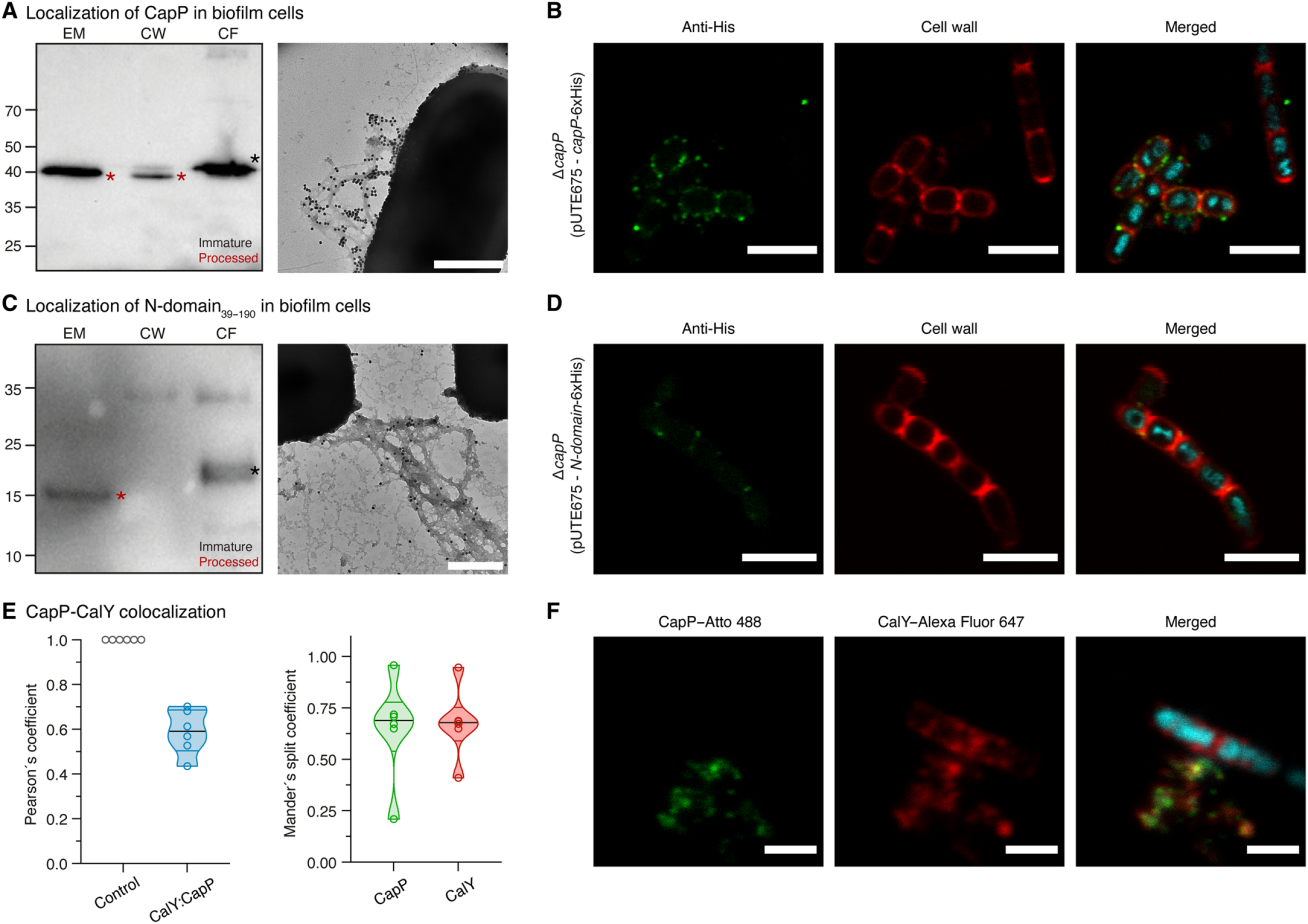
CapP is a cell wall–associated protein that colocalizes with CalY in the ECM. (**A**) Left: Western blot analysis of full-length CapP fused to a C-terminal His tag in extracellular medium (EM), cell wall (CW), and membrane/cytosolic (FC) fractions of the Δ*capP* strain after 48 hours of biofilm growth. Overexpression was induced with 10 μM IPTG using the pUTE657 vector. Immunoreactive bands were detected with an anti-His antibody. Cropped and spliced images are shown. The experiment was repeated three times with similar results. Right: Negative-stain TEM images of CapP-His immunolabeled with anti-His and nanogold-conjugated secondary antibodies. Images were collected from two independent experiments, with three to five representative fields per condition. Scale bar, 500 nm. (**B**) Immunolocalization of CapP-His in biofilm samples visualized by CLSM using an anti-His primary antibody and Atto 488–conjugated secondary antibody (green). The cell wall was labeled with WGA (red) and DNA with Hoechst (blue). Images were collected from three independent experiments, analyzing at least three fields per sample. Scale bars, 5 μm. (**C**) Western blot analysis of the truncated N-domain_39–190_-His in EM, CW, and CF fractions of the Δ*capP* strain after 48 hours. Overexpression was induced with 10 μM IPTG using the pUTE657 vector. Detection was performed with an anti-His antibody. Cropped and spliced blots are shown. The experiment was repeated three times with similar results. Right: Negative-stain TEM images of N-domain_39–190_-His immunolabeled with anti-His and nanogold-conjugated secondary antibodies. Images were collected from two independent experiments, with three to five representative fields per condition. Scale bar, 500 nm. (**D**) CLSM imaging of N-domain_39–190_-His localization in biofilm samples, with detection as in (B). Images were collected from three independent experiments, analyzing at least three fields per sample. Scale bars, 5 μm. (**E**) Colocalization analysis between CapP and CalY. Pearson’s correlation coefficient and Mander’s split coefficients were calculated from at least six fields of view across three independent experiments. (**F**) CLSM immunolocalization of CapP (green) and CalY (red). CapP was labeled with an anti-His primary antibody and Atto 488–conjugated secondary antibody and CalY with an anti-CalY primary antibody and Alexa Fluor 657–conjugated secondary antibody. DNA was stained with Hoechst (blue). Images were collected from three independent experiments, analyzing at least three fields per sample. Scale bars, 2 μm.

Immunoblotting of fractionated samples revealed that CapP-His was present in the ECM, cell wall, and membrane/cytoplasmic fractions (Fig. 5A). Bands in the membrane/cytoplasmic fractions corresponded to the immature protein, whereas smaller bands in the extracellular medium and cell wall reflected the processed, secreted form (~4.56-kDa difference due to signal peptide cleavage). TEM with anti-His immunogold labeling showed discrete gold particles regularly distributed along filaments emerging from the cell surface, suggesting that CapP-His is associated with matrix fibers (Fig. 5A). CLSM further confirmed partial localization at the cell envelope: CapP-His–specific fluorescence was detected in discrete foci along the cell wall, often adjacent to wheat germ agglutinin (WGA)–stained peptidoglycan (Fig. 5B and fig. S4, C and D).

To assess the contribution of the domains of CapP to its localization, we expressed only the N-terminal domain (N-domain_39–190_-His) in Δ*capP* cells. This construct restored biofilm formation without induction (fig. S4, A and B, and tables S17 and S18), suggesting functional activity. Fractionation assays showed that the N-domain_39–190_-His localized to the extracellular and cytoplasmic fractions but was absent from the cell wall (Fig. 5C), indicating that the C-domain mediates cell wall retention. In these assays, the N-domain_39–190_-His appeared as two bands: a ~23-kDa immature form in the membrane/cytoplasmic fraction and a ~17-kDa processed form in the extracellular medium, mirroring the processing observed for full-length CapP-His. TEM confirmed the presence of N-domain_39–190_-His–associated foci along extracellular filaments, whereas CLSM showed no detectable N-domain signal at the cell surface (Fig. 5, C and D). Together, these observations indicate that the C-domain anchors CapP to the cell wall, while the N-domain contributes to matrix assembly in the ECM.

We next investigated whether CapP colocalizes with CalY or TasA in the ECM. Using the functional CapP-His construct in Δ*capP* cells, we performed immunocytochemistry with specific antibodies against TasA, CalY, and His. CLSM revealed strong colocalization of CapP-His and CalY along ECM fibrils (Pearson’s coefficient = 0.59 ± 0.10; Fig. 5, E and F), with Mander’s coefficients indicating ~65% pixel overlap (Fig. 5, E and F). Notably, CapP-His appeared as discrete foci along these CalY-positive filaments (Fig. 5F). In contrast, no significant colocalization was observed between CapP-His and TasA (Pearson’s coefficient = 0.19 ± 0.21; overlap <26%; fig. S4, E and F).

These findings support a model in which CapP associates preferentially with CalY in the matrix, potentially guiding or stabilizing its polymerization. The absence of colocalization with TasA further highlights a division of labor in filament assembly, consistent with earlier data showing distinct structural roles for TasA and CalY.

### CapP self-assembles via its N-terminal domain into oligomeric complexes

**Fig. 6.**
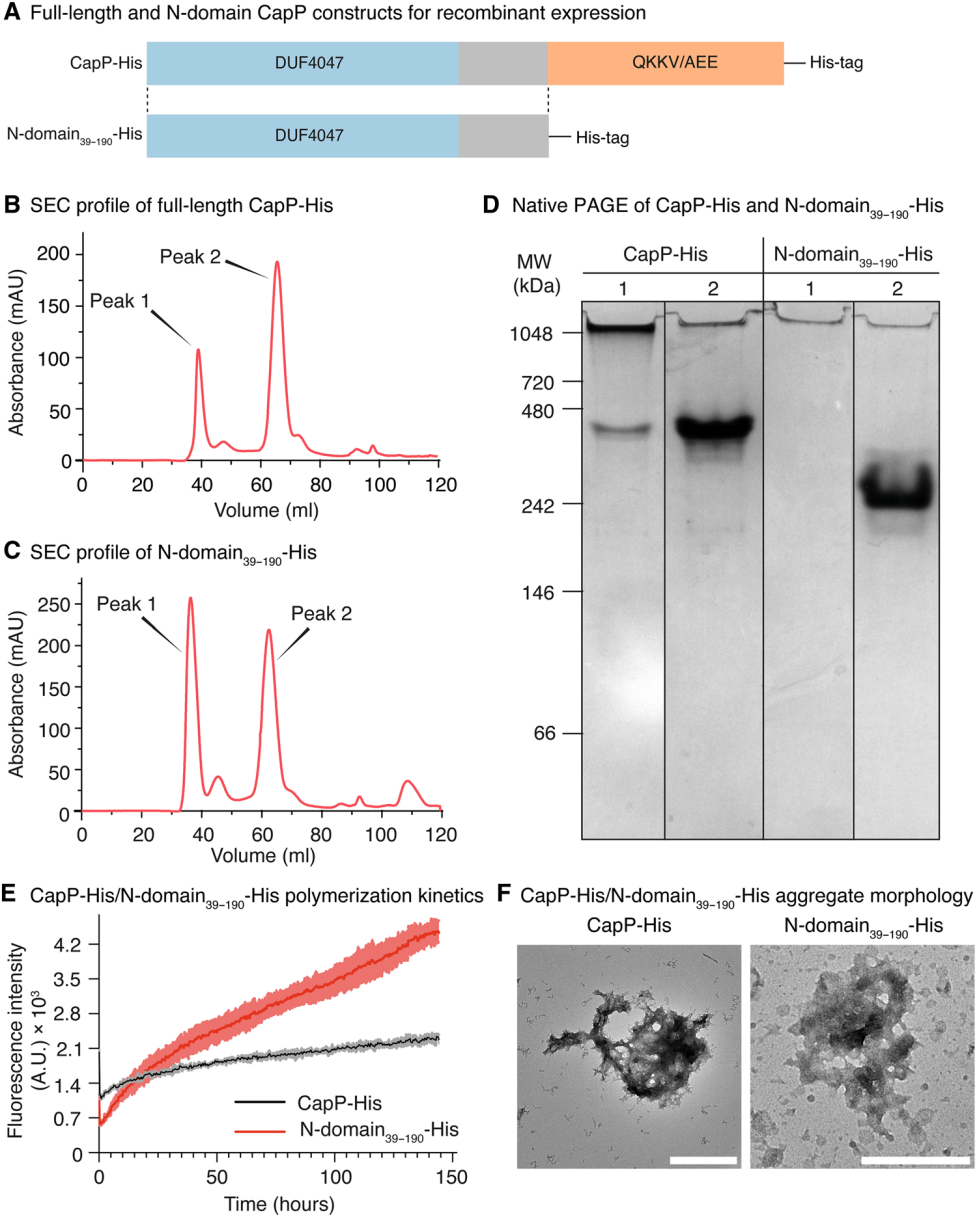
Heterologously expressed CapP oligomerizes into macromolecular complexes via its N-terminal domain. (**A**) Schematic representation of two constructs based on the full-length CapP sequence. Each construct was fused to a C-terminal polyhistidine tag and expressed recombinantly in *E. coli*. (**B**) Size exclusion chromatography (SEC) profile of full-length CapP-His purified under native conditions. (**C**) SEC profile of the N-domain_39–190_-His purified under native conditions. (**D**) Native PAGE of CapP-His and N-domain_39–190_-His following native purification. Numbers indicate the first and second peaks identified by SEC. Gel images were cropped and spliced for clarity; lines mark lane boundaries. MW, molecular weight. (**E**) Aggregation kinetics of CapP-His (black) and N-domain_39–190_-His (red) monitored by ThT fluorescence. A.U., arbitrary units. Results are representative of three independent experiments with three technical replicates each; error bars represent ± SD. (**F**) Transmission electron micrographs of CapP-His and N-domain_39–190_-His aggregates at the end of the polymerization assays shown in (E). Scale bars, 1 μm (CapP-His) and 500 nm (N-domain_39–190_-His). mAU, milli–absorbance units.

To assess the oligomerization behavior of CapP, we heterologously expressed and purified both the full-length protein and a truncated version comprising only the N-terminal domain (residues A^39^ to G^190^) (Fig. 6A). Proteins were expressed in *E. coli* Lemo21 (DE3), purified under soluble conditions by affinity and size exclusion chromatography, and verified by tandem mass spectrometry (fig. S5A). Size exclusion chromatography revealed that both constructs eluted in two distinct peaks, indicating the formation of multiple oligomeric species under native buffer conditions [20 mM tris and 50 mM NaCl (pH 7.0); Fig. 6, B and C]. Dynamic light scattering confirmed this heterogeneity, with particle sizes ranging from ~10 to 600 nm (fig. S5, B and C, and table S19). Notably, the second elution peak for the N-terminal construct contained a higher proportion of small oligomers (~53%) compared to the full-length CapP (~18%), suggesting a greater tendency to form defined assemblies in the absence of the C-domain (fig. S5, B and C, and table S19). Native polyacrylamide gel electrophoresis (PAGE) corroborated these findings: First-elution samples failed to enter the gel, indicating aggregates of >1000 kDa, whereas second-elution samples resolved as discrete bands at ~480 kDa for CapP and ~240 kDa for the N-domain, both equivalent to ~15-mers (Fig. 6D). Moreover, TEM imaging confirmed that the first fractions contained amorphous, polydisperse aggregates, while the second fractions consisted of smaller, more uniform complexes (fig. S5, D and E).

To characterize the assembly dynamics of these small oligomers, we monitored thioflavin T (ThT) (*41*) binding as an indicator of polymerization. Both proteins exhibited rapid signal increases without a lag phase, indicating preformed oligomers that act as nucleation seeds (Fig. 6E). The N-domain_39–190_-His construct displayed a more pronounced polymerization curve and reached twice the fluorescence intensity of the full-length protein after 150 hours, consistent with its higher seeding efficiency. Last, TEM analysis of late-stage aggregates from both proteins revealed amorphous, nonfibrillar particles reminiscent of CalY assemblies but distinct from the ordered filaments formed by TasA (Fig. 6F) (*37*). These findings demonstrate that the N-terminal domain of CapP mediates oligomerization and that removal of the C-domain enhances the formation of small, aggregation-prone complexes, which is potentially relevant to the proposed function of CapP as a chaperone in ECM polymerization.

### CapP N-domain nucleates rigid β-structured cores, and the C-domain modulates assembly dynamics

**Fig. 7.**
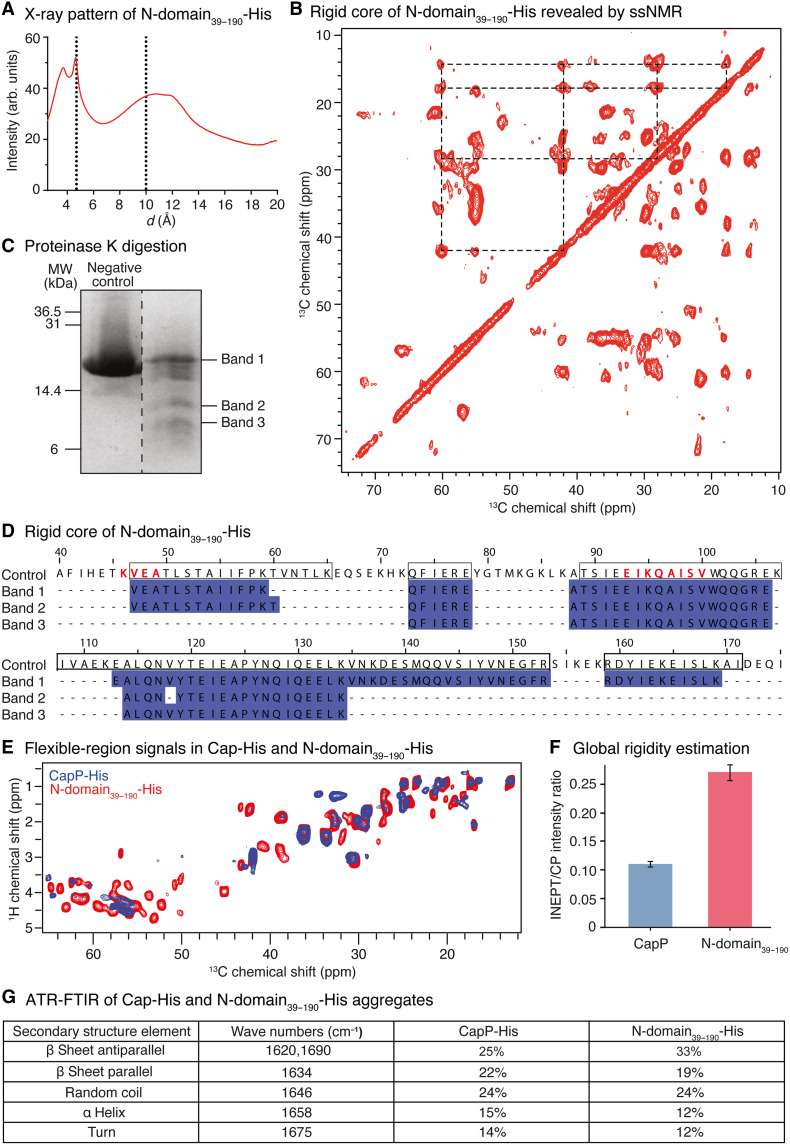
The N-domain_39–190_ assembles into a rigid core enriched in β sheets, while the C-domain modulates global protein rigidity. (**A**) X-ray diffraction pattern of N-domain_39–190_-His showing reflections at 4.7 and 10 Å, corresponding to interstrand and intersheet spacings, respectively. (**B**) Two-dimensional (2D) ^13^C-^13^C PDSD spectrum at 50 ms for N-domain_39–190_-His, highlighting chemical shifts of isoleucine residues in β sheet conformation. (**C**) SDS-PAGE of N-domain_39–190_-His aggregates after 45-min proteinase K digestion. The three marked bands were analyzed by tandem mass spectrometry. (**D**) Mapping of the rigid core of N-domain_39–190_-His based on MS analysis of the selected bands after proteinase K digestion. Boxed amino acids indicate rigid regions in the absence of proteinase K, and residues in red were identified by SSNMR PDSD at 50 and 200 ms. (**E**) Superimposed ^1^H-^13^C INEPT spectra of CapP-His (blue) and N-domain_39–190_-His (red) recorded at 600 MHz (^1^H frequency) showing signals corresponding to flexible regions. Spectra were acquired at 276 K with 96 scans for CapP-His and at 300 K with 640 scans for N-domain_39–190_-His. (**F**) Estimation of global rigidity based on the INEPT/CP intensity ratio for CapP-His and N-domain_39–190_-His. (**G**) Secondary structure analysis of CapP-His and N-domain_39–190_-His aggregates by ATR-FTIR spectroscopy. Representative spectra are shown; experiments were repeated three times with similar results.

Given that the N-domain of CapP retains both biofilm-rescuing activity and the ability to form oligomers, we initiated structural analysis using this truncated construct (residues 39 to 190). Bioinformatic predictions using FoldUnfold (*42*) indicated that most of the N-terminal region is structured, whereas the C-terminal repeats are largely unfolded (fig. S5F). Moreover, AlphaFold modeling (*43*) predicted a well-defined α-helical region within the N-domain, consisting of three α helices arranged in a helical bundle (fig. S5G). The N-terminal beginning, which contains the signal peptide, and the C-domain region were both associated with low-confidence values, consistent with structural disorder. It should be noted, however, that these in silico predictions do not account for the physiological environment of the protein, and the low pLDDT scores indicate that these regions should be interpreted with caution. X-ray diffraction of N-domain_39–190_-His assemblies instead revealed cross-β reflections at ~4.7 and ~10 to 11 Å, characteristic of amyloid-like architectures (Fig. 7A) (*44*). To further probe these structural features, aggregation-prone segments were identified using multiple bioinformatic tools (fig. S7F) (*45*–*49*), revealing three main regions L^51^-P^58^, I^98^-S^99^, and V^144^-N^149^, which align with hydrophobic segments predicted by ProtScale (fig. S5H) (*50*).

Magic-angle spinning (MAS) solid-state nuclear magnetic resonance (SSNMR) of uniformly ^13^C/^15^N-labeled N-domain assemblies provided further resolution. Dipolar-based two-dimensional (2D) ^13^C-^13^C spectroscopy, using cross-polarization transfer, identified ~35 to 40 spin systems, consistent with a central rigid core enriched in β sheet conformation. For example, isoleucine residues showed characteristic chemical shifts [Cα, ~60 parts per million (ppm); Cβ, ~42 ppm], indicative of β sheet secondary structure (Fig. 7B). Proteinase K digestion followed by mass spectrometry (MS) confirmed the presence of protease-resistant segments (V^47^-K^59^ and A^88^-K^134^) (Fig. 7, C and D). Further examination of the SSNMR data using a 200-ms proton-driven spin diffusion (PDSD) experiment (fig. S6A) enabled sequential assignment of residues K^46^-A^49^ and E^93^-V^100^ within these protease-resistant regions. In addition, the spectral resolution (60 to 120 Hz full width at half-height) indicated a relatively well-ordered arrangement of subunits within the complex.

To examine the contribution of the C-domain, we analyzed full-length CapP-His under similar conditions. Upon ultracentrifugation, full-length CapP-His formed a viscous, gel-like pellet, unlike the white precipitate of the N-domain_39–190_-His. X-ray diffraction again revealed β-structure signatures (~4.7 Å), and 2D SSNMR spectra of both constructs overlapped substantially, indicating that the rigid β-core of the N-domain is preserved in the full-length protein (fig. S6, B and C). Only a few additional signals were observed in full-length CapP-His, suggesting that the C-domain does not substantially contribute to the rigid core structure.

To assess dynamic regions (*51*), we used J-coupling polarization transfer SSNMR to detect flexible residues. Full-length CapP-His displayed markedly fewer insensitive nuclei enhanced by polarization transfer (INEPT) signals compared to the N-domain_39–190_-His (Fig. 7E), implying greater global rigidity. Quantitative comparison of 1D ^13^C-detected INEPT/CP (cross-polarization) signal ratios confirmed this difference (0.1 for full-length CapP-His versus 0.25 for the N-domain_39–190_-His; Fig. 7F), indicating that the C-domain contributes to reduced conformational mobility. Last, attenuated total reflection Fourier transform infrared (ATR-FTIR) spectroscopy was used to compare the secondary structure content. While both proteins contained a mix of β sheets, turns, and random coil elements, the N-domain_39–190_-His exhibited a higher proportion of antiparallel β sheets (33% versus 25%), whereas full-length CapP-His showed more parallel β sheets and α helices (Fig. 7G and fig. S6, D to F). These results suggest that the C-domain introduces additional structure and reduces flexibility, potentially stabilizing CapP assemblies in the ECM.

### CapP modulates CalY polymerization via a concentration- and domain-dependent chaperone-like mechanism

**Fig. 8.**
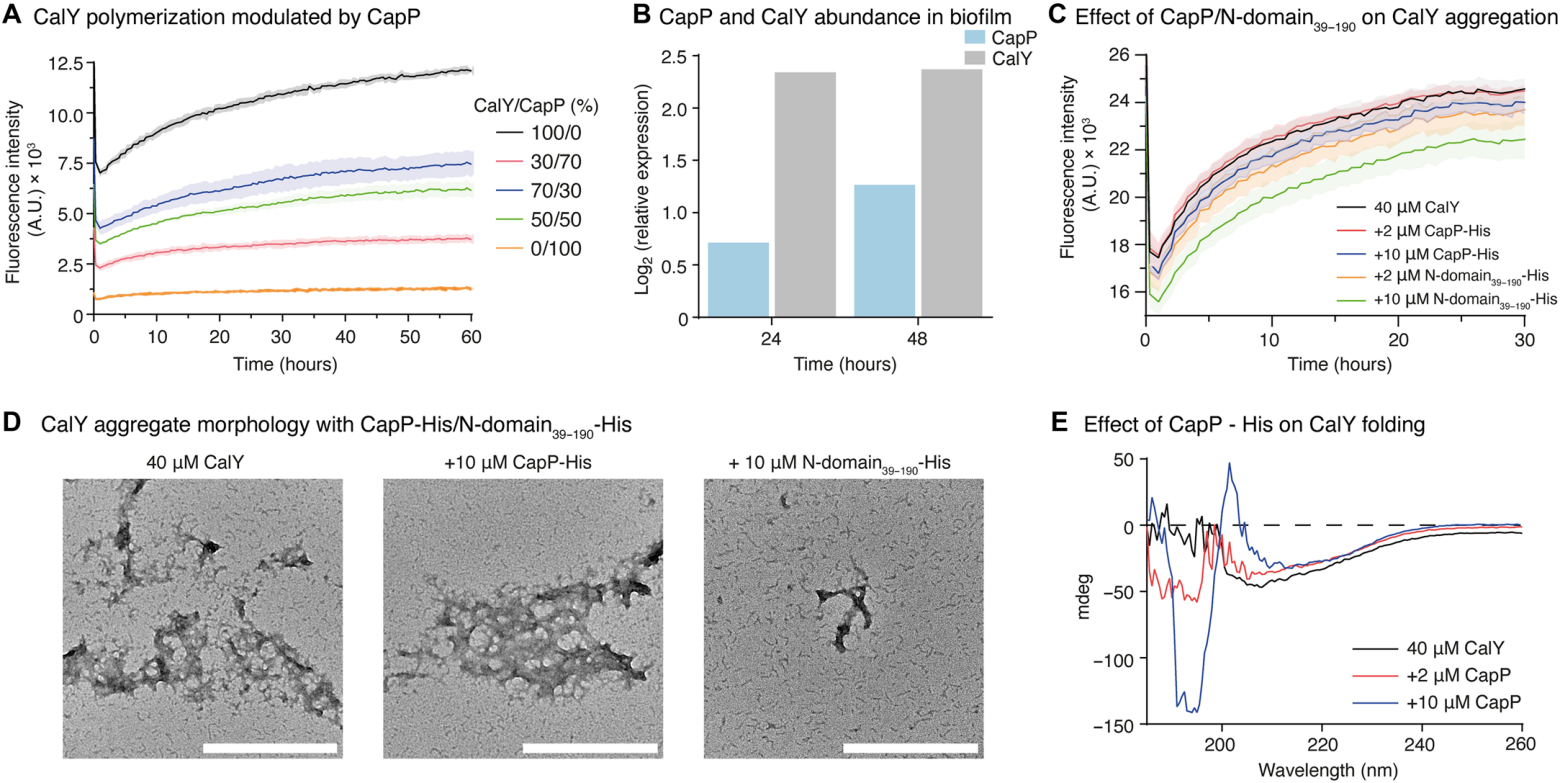
CapP acts as a chaperone for CalY, with its activity modulated by the C-domain. (**A**) Polymerization kinetics of CalY at different CalY:CapP-His molar ratios monitored by ThT fluorescence. Results are representative of three independent experiments with three technical replicates each; error bars represent ± SD. (**B**) Relative abundance of CapP and CalY during biofilm formation determined by iTRAQ analysis. (**C**) ThT fluorescence kinetics of CalY aggregation in the presence of CapP-His or N-domain_39–190_-His at 2 and 10 μM. Results are representative of three independent experiments with three technical replicates each; error bars represent ± SD. (**D**) Transmission electron micrographs of negatively stained CalY aggregates formed in the presence of CapP-His or N-domain_39–190_-His at 10 μM after 30 hours of incubation at 37°C. Scale bars, 500 nm. (**E**) Circular dichroism spectra of CalY incubated alone or with CapP-His at 2 and 10 μM after 16 hours under agitation at 200 rpm at 37°C. Representative spectra are shown; experiments were repeated three times with similar results.

The in vivo colocalization of CapP and CalY in the ECM, along with the observed disruption of CalY filament formation in Δ*capP* strains, suggested that CapP may directly influence CalY assembly. To test this, we monitored CalY polymerization in vitro in the presence of increasing concentrations of CapP-His using ThT fluorescence. At CalY:CapP molar ratios of 70:30 and 50:50, CalY polymerization kinetics were markedly altered compared to CalY alone (Fig. 8A). This inhibitory effect, together with previous evidence that CapP levels are tightly regulated in vivo, prompted us to quantify the relative abundance of CalY and CapP during biofilm development using iTRAQ proteomic analysis. At 24 hours, CalY levels were approximately 3.3-fold higher than those of CapP; by 48 hours, this ratio decreased to ~2:1 (Fig. 8B and fig. S7A), suggesting that CapP accumulates during the transition to mature biofilms. Using these physiological ratios, we performed copolymerization assays. At low CapP-His concentrations (2 and 10 μM), no notable changes were observed in the polymerization of CalY (40 μM). However, when the N-terminal domain (N-domain_39–190_-His) was added at the same concentrations, CalY polymerization was strongly delayed, with reduced final fluorescence intensity (Fig. 8C). This effect was more pronounced when using aged protein preparations enriched in preformed oligomers (fig. S7, B and C), suggesting that oligomeric CapP is particularly effective at interfering with CalY nucleation. TEM imaging supported these findings. While coincubation with full-length CapP-His produced aggregates similar to those of CalY alone, coincubation with the N-domain_39–190_-His led to the accumulation of small oligomers, not fibrils (Fig. 8D). These results are consistent with in vivo data showing impaired CalY filament formation in the Δ*C-domain* strain (Fig. 4G), reinforcing the idea that the C-domain region influences CalY polymer growth.

To explore whether CapP affects the CalY secondary structure, we performed circular dichroism analysis after 16 hours of agitation at 200 rpm (fig. S7D). After removing the CapP contributions from the spectra, DichroWeb (*52*) analysis indicated that in the presence of 10 μM CapP-His, CalY exhibited an increased β sheet content compared with the control or the low CapP-His concentration (2 μM) (Fig. 8E and table S20). These results confirm that CapP not only modulates the rate of CalY polymerization but also alters its structural state.

Together, our data support a model in which CapP acts as a concentration- and domain-dependent chaperone-like modulator of CalY polymerization. This role is likely fine-tuned during biofilm development to control the assembly and organization of the ECM.

## DISCUSSION

The ECM of *B. cereus* remains poorly defined, with strain-specific variability complicating efforts to understand its role in persistence, virulence, and resistance (*53*). Our study identifies a tripartite protein system—TasA, CalY, and CapP—that governs ECM assembly in *B. cereus* ATCC14579 via a sortase-independent mechanism. We show that TasA and CalY form heteropolymeric filaments essential for ECM integrity and that their assembly is strictly dependent on CapP, a chaperone-like protein previously annotated as BC1280. Deletion of CapP strongly impairs biofilm formation, underscoring its pivotal role.

Although functionally reminiscent of TapA in *B. subtilis* (*14*), CapP differs in expression timing, localization, and mechanism. TapA is active early and in planktonic cells, while CapP is exclusively expressed in biofilm-associated cells and cannot be replaced by TapA in functional complementation assays (*30*). Neither TasA nor CalY filaments form in Δ*capP* mutants, even when their expression is artificially restored, demonstrating that the role of CapP extends beyond transcriptional regulation. Structural and biochemical analyses revealed that CapP’s N-terminal domain mediates self-assembly into oligomers, forming a rigid β sheet–rich core. This domain alone can rescue biofilm formation but lacks full localization to the cell wall. The disordered C-domain appears to anchor CapP to the surface and modulate its chaperone function. In vitro, CapP regulates CalY polymerization in a concentration- and domain-dependent manner, promoting filament formation at low levels and inhibiting it at higher levels, resembling behaviors of known extracellular chaperones. Unlike sortase-linked pili or disulfide-crosslinked fibers like Ena, CapP-mediated filamentation constitutes a unique, sortase-independent strategy (*54*–*56*). In this system, CapP nucleates or stabilizes CalY, which then recruits TasA into heterofilaments. These observations place *B. cereus* ECM biogenesis into a broader mechanistic framework, alongside type IV pili, curli, and chaperone-usher pathways (*57*, *58*), while establishing CapP as a defining element of a previously uncharacterized Gram-positive matrix system. Using SSNMR and ATR-FTIR, we showed that CapP’s N-domain forms a rigid core enriched in β sheets, involving hydrophobic residues critical for nucleation (e.g., K^46^VEATLSTAIIFP^58^ and E^93^IKQAISV^100^). The C-domain, in contrast, enhances structural rigidity but is not part of the core, reinforcing its proposed role in spatial regulation.

Our findings also highlight *B. cereus* ECM adaptability. Deleting *tasA* triggers overexpression of flagellar components and CalY rearrangement; conversely, Δ*calY* induces eDNA overproduction and a TasA-eDNA interaction pattern. These phenotypes suggest feedback mechanisms that maintain ECM cohesion despite loss of key components. Notably, increased biomass in Δ*tasA* was linked to enhanced flagellin production at the protein, but not RNA, level—implying posttranscriptional regulation, a mechanism also described in *B. subtilis* via EpsE (*59*). This is reminiscent of the regulatory role of TasA in *B. subtilis*, where it maintains a motile subpopulation within the biofilm through modulation by the CssRS two-component system, suggesting that TasA-dependent control of motility may be a conserved feature across *Bacillus* species (*34*). In *B. cereus*, although the Spo0A-SinI/SinR axis is conserved and cssR/S-like homologs are present, an equivalent TasA-sensing pathway has not been demonstrated; together with the absence of TapA and the presence of CapP-TasA-CalY, this points to species-specific matrix assembly and regulation. The ECM of Δ*calY* mutants showed that TasA foci colocalized with eDNA, raising the possibility that eDNA compensates for protein loss by enhancing cell aggregation. This is consistent with the roles described for eDNA in *Staphylococcus aureus* and *Bacillus licheniformis*, where it interacts with amyloid-like matrix proteins (*60*–*62*). Investigating whether TasA or CalY binds directly to eDNA, and how such interactions modulate polymerization, will be an important next step.

**Fig. 9.**
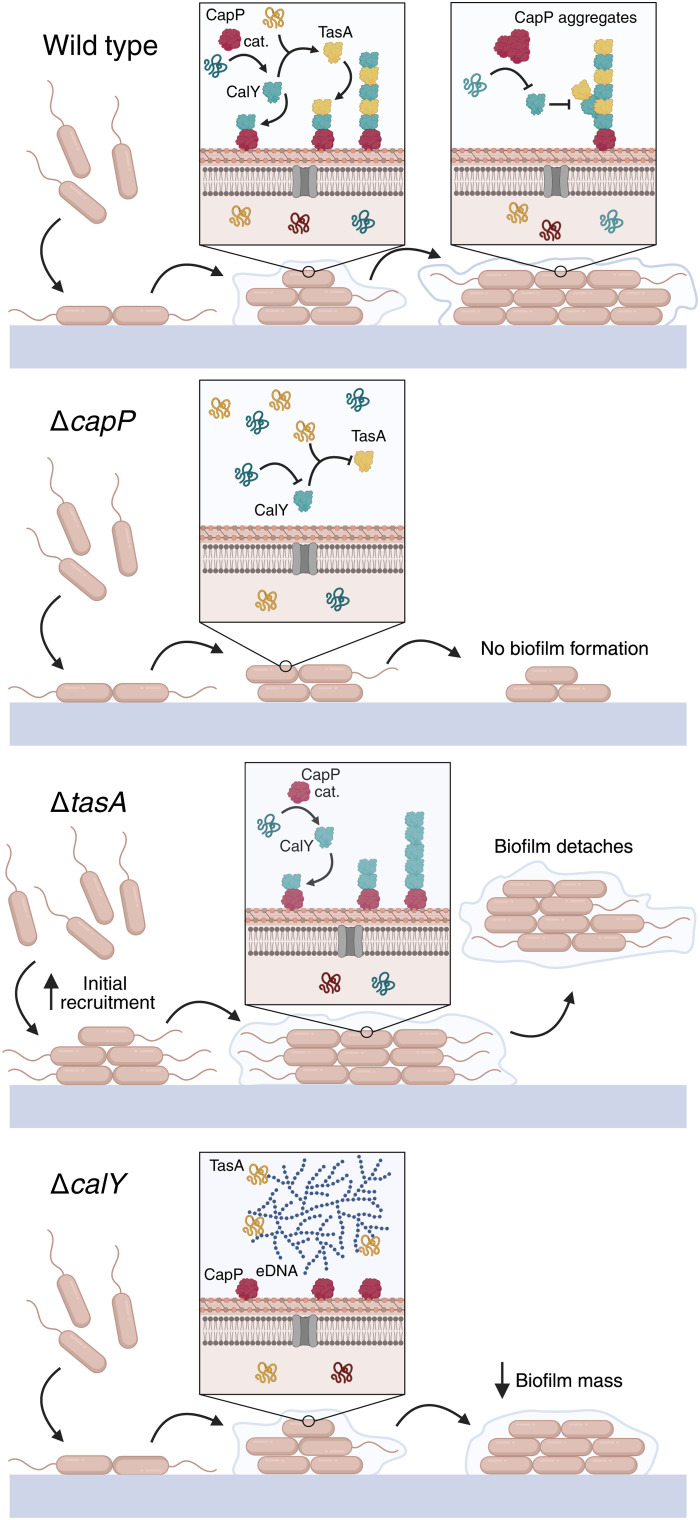
Model of CapP-regulated TasA-CalY filament assembly in the *B. cereus* ECM. Secretion of TasA and CalY precedes biofilm formation, while CapP is secreted at its onset; all three are processed by the signal peptidase SipW. At early stages (24 hours), CapP interacts with unfolded CalY to initiate fibril formation, facilitating the incorporation of TasA into growing filaments. As the biofilm matures (48 hours), CapP levels increase, and CapP-CalY complexes stabilize and arrest further filament elongation. In the absence of *capP*, both CalY and TasA fail to polymerize in the ECM, thereby preventing biofilm formation. In the *tasA* mutant, enhanced initial adhesion occurs due to flagellum overexpression, and CalY filaments partially compensate for the absence of TasA. However, biofilm detachment eventually occurs due to impaired adhesion to abiotic surfaces. In the absence of CalY, excess eDNA is produced, and TasA colocalizes with it instead of polymerizing, partially compensating for lost cell-cell interactions and resulting in diminished biofilm formation. cat., catalyzes. Created in BioRender. Lab, B. (2026) https://BioRender.com/u44gcun.

We propose a model wherein CapP initiates and controls the assembly of TasA-CalY filaments in response to changing protein levels during biofilm maturation. Before biofilm formation, during planktonic growth, *sipW-tasA* transcription begins in early stationary phase, approximately 1 hour earlier than *calY* transcription, indicating that TasA may become available before CalY and prime early assembly events (*31*). Upon transition from planktonic to sessile growth, low CapP concentrations at early stages promote polymerization via interaction with CalY. As CapP levels rise, stable CapP-CalY complexes limit filament growth, thereby modulating ECM architecture. In the absence of CapP, TasA and CalY fail to assemble, halting biofilm formation. Meanwhile, Δ*tasA* and Δ*calY* strains display distinct compensatory ECM profiles involving flagella and eDNA, respectively (Fig. 9).

Together, our findings support a model of ECM organization in *B. cereus* that is both regulated and structurally flexible. The compensatory behaviors observed in ∆*tasA* and *∆calY* mutants—manifested by changes in eDNA, flagellar expression, and filament rearrangement—highlight the capacity of the matrix to reorganize under genetic perturbation. Such modularity and adaptive potential are not unique to *B. cereus*; similar patterns of ECM flexibility have been documented in *S. aureus* (*63*), *B. subtilis* (*64*), and *Pseudomonas aeruginosa* (*65*), where matrix components dynamically shift in response to environmental or genetic cues. These shared properties suggest that ECM flexibility may represent an evolutionarily conserved strategy for maintaining biofilm integrity. Across diverse bacterial taxa, functional redundancy and component interchangeability likely provide resilience to fluctuating conditions, ensuring structural robustness and long-term colonization. Recognizing this flexibility not only broadens our understanding of bacterial multicellularity but also underscores the importance of studying ECM organization at the species level, even within closely related genera.

In conclusion, our findings uncover a previously uncharacterized, sortase-independent mechanism for ECM protein polymerization in Gram-positive bacteria, centered on CapP as a concentration- and domain-dependent chaperone-like protein. This work substantially enhances our understanding of *B. cereus* biofilms and provides a conceptual framework for targeting ECM assembly in clinical and industrial settings.

## MATERIALS AND METHODS

### Bacterial strains and culture conditions

The bacterial strains used in this study are listed in table S21. For experiments conducted using *B. cereus* wild-type or mutant strains, cultures were routinely grown at 28°C from frozen stocks on Luria-Bertani (LB) plates containing 1% tryptone (Oxoid), 0.5% yeast extract (Oxoid), 0.5% NaCl, and 1.5% agar before the respective experiments were performed. Biofilm assays were performed in tryptone-yeast extract (TY) medium supplemented with 1% tryptone (Oxoid), 0.5% yeast extract (Oxoid), 0.5% NaCl, 10 mM MgSO_4_, and 1 mM MnSO_4_.

For cloning and plasmid replication, the *E. coli* DH5α strain was used. For recombinant protein expression and purification, *E. coli* BL21 (DE3) pLysS (Merck, Kenilworth, NJ, USA) was used. All the strains were cultured in LB liquid broth at 28°C. For the agar-solid plates, LB medium was supplemented with 1.5% bacteriological agar (Oxoid). The final antibiotic concentrations were 100 μg/ml of ampicillin, 50 μg/ml of kanamycin, 100 μg/ml of spectinomycin, and 5 μg/ml of erythromycin. For the purification of ^13^C/^15^N-labeled proteins, the cultures were grown in M9 minimal medium (the final concentration was 48 mM Na_2_HPO_4_, 22 mM KH_2_PO_4_, 8.6 mM NaCl, 1 mM MgSO_4_, 0.01 mM ZnCl_2_, 0.001 mM FeCl_3_, 0.1 mM CaCl_2_ and 10 ml of 100× minimum essential medium vitamin solution) supplemented with ^15^NH_4_Cl (1 g/liter) as nitrogen and ^13^C_6_-d-glucose or 2-^13^C glycerol (2 g/liter) as the carbon source.

### Plasmid and strain construction

All the primers used to construct the strains used in this study are listed in table S22. *B. cereus* mutant strains were constructed via homologous recombination using the upstream and downstream regions of the gene of interest, which were subsequently cloned and inserted into the pMAD plasmid (*66*). The constructs were designed using NEB Builder HiFi DNA Assembly Master Mix (New England Biolabs, MA, USA) following the manufacturer’s instructions and using specific primers. The pMAD vector was linearized using the restriction enzyme SmaI (FastDigest, Thermo Fisher Scientific), and the digested vector was incubated at 50°C for 1 hour along with the respective upstream and downstream fragments. The combined total amount of fragments and plasmid was 0.2 pmol, and a proportion of 1:2 (vector:fragments) was maintained. The resulting reaction mixture was subsequently transformed into *E. coli* DH5α, and positive colonies were selected using ampicillin. Subsequently, the plasmids were purified, subjected to PCR amplification and digestion for verification, and sequenced.

For the overexpression experiments in *B. cereus*, the gene of interest was cloned and inserted into the plasmid pUTE657 (*26*) under a promoter that was inducible by IPTG using the primers listed in table S22. Following gene amplification and the inclusion of a specific ribosome binding site (RBS) for *Bacillus* (5′-agagaacaaggaggg-3′), the resulting PCR product was digested with SalI and SphI (FastDigest, Thermo Fisher Scientific) and subsequently cloned and inserted into the pUTE657 plasmid that was cut with the same restriction enzymes using T4 DNA ligase (Thermo Fisher Scientific). Subsequently, the resulting reaction was transformed into *E. coli* DH5α, and colonies were selected with ampicillin (100 μg/ml). After plasmid purification, all the vectors were subjected to PCR amplification, digestion with restriction enzymes, and sequencing.

The vectors were transformed into *B. cereus* by electroporation following previously described methods with some modifications. A single colony from a pure culture of *B. cereus* was inoculated in 5 ml of LB medium and incubated under shaking conditions at 180 rpm at 30°C for 16 hours. This culture was subsequently used to inoculate a flask containing 100 ml of LB, which was incubated at 30°C with at 180 rpm until an optical density at 600 nm (OD_600_) of 0.3 was reached. The cell culture mixture was centrifuged (1200*g*, 5 min, 4°C), and the resulting pellet was resuspended in 12 ml of electroporation buffer (0.5 mM K_2_HPO_4_, 0.5 mM KH_2_PO_4_, 0.5 mM MgCl_2_, and 272 mM saccharose). The suspension was centrifuged again and then resuspended in 250 μl of electroporation buffer. The electrocompetent cells were mixed with 0.5 to 1 μg of plasmid and incubated on ice for 10 min. Next, the mixture was electroporated in a 0.2-cm cuvette using a voltage of 1.4 kV, a capacitance of 25 μF, and a resistance of 200 Ω. After electroporation, the suspension was recovered and incubated with 1.5 ml of LB for 5 hours at 30°C at 180 rpm. The resulting culture was plated onto LB plates supplemented with the corresponding antibiotic to select the colonies that had been successfully transformed with the vector. For integration of the deletion, *B. cereus* transformants were incubated without antibiotics at 40°C, facilitating the integration of the mutation into the genome through homologous recombination. Last, the culture was plated on LB agar plates, and the colonies were picked onto LB agar plates supplemented with erythromycin. Colonies that did not grow on LB supplemented with the antibiotic were selected, and the mutation was confirmed through PCR amplification.

For heterologous expression in *E. coli*, CapP-His and the N-domain_39–190_-His, corresponding to amino acids 39 to 190 of the *bc1280* gene, were cloned and expressed. The PCR products and the pET24b vector were digested with NdeI and XhoI (FastDigest, Thermo Fisher Scientific) and subsequently cloned using T4 DNA ligase (Thermo Fisher Scientific) following the instructions supplied by the manufacturer. The reaction mixtures were subsequently transformed into *E. coli* DH5α, after which the colonies were successfully transformed with kanamycin (50 μg/ml).

### Phylogenetic tree

The amino acid sequence of BC1280 was obtained from the Kyoto Encyclopedia of Genes and Genomes (KEGG) database using *B. cereus* ATCC14579 as a reference. BLASTP (Basic Local Alignment Search Tool for Proteins) analysis was performed, and the top 50 hits were aligned using Clustal Omega (*67*). A phylogenetic tree was then constructed using neighbor-joining method in MEGA11 (*68*) with 1000 bootstrap replications, the Poisson substitution model with uniform rates among sites, complete deletion of gaps/missing data, and seven computational threads.

### Reverse transcription quantitative polymerase chain reaction

RT-qPCR was used to estimate the transcription levels of (i) *bc1280* in planktonic and biofilm cells of the wild-type strain; (ii) *tasA*, *calY*, *eps1*, and *eps2* in Δ*capP* compared to those in the wild type; and (iii) *tasA*, *calY*, *eps1*, and *eps2* in Δ*capP* overexpressing *bc2794* or *bc2793-2794* compared to those in the wild type. RT-qPCR was performed using an iCycler-iQ system (Bio-Rad) and Power SYBR Green Master Mix (Thermo Fisher Scientific). Primer3 software (https://primer3.ut.ee/) was used for primer design, maintaining the default parameters (*69*). First, 1 μg of total RNA was reverse transcribed into cDNA using SuperScript III reverse transcriptase (Invitrogen) following instructions provided by the manufacturer. The reactions were performed in triplicate in 96-well plates with a total volume of 20 μl. The RT-qPCR cycle started with an initial denaturation at 95°C for 3 min, followed by 40 amplification cycles (95°C for 20 s, 56°C for 20 s, and 72°C for 30 s), and the final step was 95°C for 30 s. The target genes, including *bc1280*, which encodes the protein BC1280 (now named as CapP); *bc1279*, which encodes TasA (also known as spore coat–associated protein N); *bc1281*, which encodes CalY (annotated as cell envelope–bound metalloprotease, camelysin); *bc5263*, which encodes UDP-glucose 4-epimerase; *bc5268*, which encodes secreted polysaccharide polymerase; *bc5277*, which encodes tyrosine protein kinase; *bc5279*, which encodes tyrosine protein kinase; and *bc1583*, which encodes *O*-acetyl transferase, were amplified using the primer pairs provided in table S23. Primer efficiency and amplification product specificity were assessed as previously described (*70*). The estimation of relative expression levels was calculated using the ΔΔCt threshold (Ct) method (*71*). The housekeeping gene *rpoA* was used as a reference for data normalization because it is constitutively expressed in *B. cereus* under the growth conditions tested, shows minimal variation across strains and time points, and has been previously validated as a reliable control in this organism (*72*). The relative expression value was calculated as the difference between the qPCR threshold cycle (Ct) of the gene of interest and the Ct obtained for *rpoA* (ΔCt = Ct_target gene_ − Ct*_rpoA_*). The results obtained for the genes of interest were normalized to the values obtained for the wild-type strain. The RT-qPCR analyses were conducted three times, each with three independent biological replicates.

### Determination of the transcriptional unit by PCR

To determine whether *capP* constitutes an operon with *tasA* or *calY*, primers were designed between the genes and inside each gene to amplify four specific fragments that were 974, 572, 1668, and 560 bp in size (primers listed in table S24). cDNA was used as the template, and PCR was performed using genomic DNA purified with the commercial JetFlex Genomic DNA Purification Kit (Thermo Fisher Scientific, Bremen, Germany) as a positive control. The fragments were amplified by PCR using Phusion High-Fidelity DNA Polymerase (Thermo Fisher Scientific) with the following program: initial denaturation at 98°C for 30 s, followed by a 30-cycle amplification program (98°C for 10 s, 56°C for 30 s, and 72°C for 1 min), and a final extension step at 72°C for 5 min. The samples were loaded onto an agarose gel and compared to the fragments amplified from genomic DNA.

### Biofilm formation and extracellular complementation assays

To study the biofilm formation phenotype for each strain or condition, bacterial cultures were grown in 24-well plates as previously described (*30*). The strains were then grown on LB agar plates at 28°C for 24 hours. The bacterial cells were collected from the plates and resuspended in 1 ml of TY medium, and the OD_600_ was adjusted to 1. Subsequently, 10 μl of the suspension was inoculated into each well containing 1 ml of TY medium. The plates were then incubated at 28°C without agitation for 72 hours.

For estimating live cell counts, biofilm cells were collected by pipetting to ensure complete detachment from the wells. After removing the supernatant from the 24-well plate, the wells were carefully washed three times with water. The biofilm ring was then fully resuspended by pipetting and subsequently passed 10 times through a syringe to disrupt potential bacterial clumps, ensuring proper homogenization and accurate quantification. The resulting suspensions were then serially diluted and plated onto TY agar plates for colony counting.

For the extracellular complementation assay involving the addition of TasA or CalY as monomers or in their fibrillated states, the proteins were previously purified in vitro from inclusion bodies through heterologous expression in *E. coli* following a protocol established by El Mammeri *et al.*, in 2019 (*37*). Subsequently, the proteins were dialyzed against a 20 mM tris and 50 mM NaCl solution at pH 7.4, after which the protein concentrations were measured. The assembly of filaments was promoted at 37°C under agitation at 200 rpm for 1 week. TasA or CalY protein, in both its monomeric and fibrillated forms, was added at a final concentration of 6 μM to 24-well plates inoculated with the Δ*capP* strain. These plates were then incubated without agitation at 28°C for 72 hours, after which the resulting biomass was stained with crystal violet.

### eDNA extraction

The isolation of eDNA from biofilm samples was performed following a previously established protocol, with a few modifications (*73*). Bacterial strains were first grown in 24-well plates containing TY medium for 72 hours. Subsequently, planktonic cells were removed, and the wells were washed with water at least three times. The remaining biofilm mass was resuspended in 1 ml of phosphate-buffered saline (PBS) by repeated pipetting and syringe passage to ensure homogenization before measuring the OD_600_. After centrifugation at 6800*g* for 4 min, the supernatant was transferred to a sterile tube. Then, 50 μl of a precipitation solution [1.5 M ammonium acetate (pH 5.3)] was added and mixed thoroughly. Next, 700 μl of the supernatant was combined with 70 μl of 2.5 M NaCl and 1400 μl of 96% ethanol, and the mixture was stored at −20°C for at least 24 hours. DNA precipitation was achieved by centrifugation (25 min, 4°C, 23,500*g*), followed by a washing step with 70% ice-cold ethanol. The resulting pellet was dried for less than 3 min at 43°C and then resuspended in TE (tris-EDTA) buffer [10 mM tris and 1 mM EDTA (pH 7.5)] by vortexing for 25 s. To determine the concentration of eDNA, each sample was measured using a NanoDrop spectrophotometer. The concentration was then normalized to the OD_600_ previously measured for the corresponding biofilm sample. A minimum of nine biological replicates per strain was used for eDNA extraction.

### Crystal violet biofilm assay

Biofilm formation on the well plate surface was quantitatively measured using crystal violet staining, as previously described (*74*). The biofilms were grown for 72 hours, after which the medium was removed. Then, 1 ml of 1% crystal violet was added to each well of the 24-well plate, and the cells were incubated for 15 min. The plates were washed multiple times with water and left to dry for 45 min. Next, the biomass that was formed in each well was resuspended in 1 ml of 50% acetic acid, and the absorbance at 575 nm was measured for each sample using a plate reader (Tecan Infinite M1000 Pro; Tecan, Männedorf, Switzerland).

For visualization, full-plate images were captured for each condition, and the central region of each well was cropped to generate the images shown in the main figures. The corresponding raw full-plate images, including scale bars, are provided in figs. S8 to S14, covering all biofilm images presented in the manuscript.

The statistical significance was evaluated using one-way analysis of variance (ANOVA) with Dunnett’s multiple comparisons test. *P* values less than 0.05 were considered to indicate statistical significance.

### Autoaggregation assay

The effect of mutant strains on the aggregation rate of *B. cereus* was estimated using a kinetic method following a previously described protocol (*75*). The strains were inoculated into a flask containing 20 ml of TY medium and incubated overnight at 28°C with agitation at 180 rpm. An aliquot of the overnight culture was centrifuged (3000*g*, 10 min, 20°C), and the resulting supernatant was used to dilute the remaining culture to a final volume of 10 ml at OD_600_ = 3. The samples were then incubated in vertical tubes under static conditions at room temperature for 24 hours, after which the OD_600_ was measured every hour at the air-liquid interface.

### RNA extraction

For RNA extraction, a previously described protocol was followed with several modifications (*32*). Bacterial strains were cultivated in 24-well plates to retrieve the desired population—either planktonic or biofilm cells—at a specific incubation time. For the biofilm cells, the supernatant was removed from each well, followed by three wash steps, and the biomass was subsequently resuspended in 1 ml of PBS. To minimize variability between replicates, each sample was prepared by extracting 500 μl from eight distinct wells. The bacterial suspensions were then centrifuged at 12,000*g* for 5 min, and the resulting pellets were frozen at −80°C for 30 min or until needed. The pellets were resuspended in 900 μl of TRI-Reagent (Merck), and the cells were disrupted with 0.1-mm beads using a TissueLyser (QIAGEN) for 3 min, followed by incubation at 55°C for 3 min. Then, 200 μl of chloroform was added to each tube, which was vortexed for 10 s and incubated at room temperature for 3 min. Last, the samples were centrifuged at 12,000*g* for 10 min at 4°C. The resulting aqueous phase was transferred to a clean tube containing 500 μl of ice-cold isopropyl alcohol. The tubes were inverted a few times, incubated for 10 min at room temperature, and then centrifuged at 12,000*g* for 10 min at 4°C. The supernatants were subsequently removed, and the pellets were washed with 1 ml of ice-cold 75% ethanol, followed by centrifugation (12,000*g*, 5 min, 4°C). Briefly, the RNA pellets were dried and then resuspended in 50 μl of diethyl pyrocarbonate–treated water. The residual DNA was eliminated through treatment with rDNase (recombinant deoxyribonuclease), which is included in the Nucleo-Spin RNA Plant Kit (Macherey-Nagel), following the instructions provided by the manufacturer. The quality and integrity of the total RNA were assessed using an Agilent 2100 bioanalyzer (Agilent Technologies) and gel electrophoresis.

### Whole-transcriptome analysis

RNA sequencing (RNA-seq) was performed by the Omics Unit of Supercomputing and Bioinnovation Center (SCBI, University of Malaga, Spain). Ribosomal RNA (rRNA) removal was achieved using the RiboZero rRNA Depletion Kit (Illumina, CA, USA), and subsequently, 100-bp single-end read libraries were prepared using the TruSeq Stranded Total RNA Kit (Illumina). Next, the libraries were sequenced using the NextSeq550 instrument (Illumina). To eliminate regions of low quality, ambiguity, and low complexity, as well as potential contamination, the raw reads were preprocessed using SeqTrimNext with the specific configuration parameters used for next-generation sequencing (NGS) technology (*76*). Subsequently, the clean reads were aligned and annotated using the *B. cereus* ATCC14579 genome (NC_004722.1) as the reference. This alignment was conducted with Bowtie2, resulting in BAM (Binary Alignment/Map) files that were subsequently sorted and indexed using the SAMtools v1.484 program (*77*, *78*). To calculate the read count for each gene, the script Sam2counts (https://github.com/vsbuffalo/sam2counts) was used to determine the number of uniquely mapped reads. The analysis of differentially expressed genes (DEGs) between mutant strains and the wild-type strain was conducted using the R script DEgenes Hunter (*79*). This script uses a combined *P* value, using the Fisher’s method to consider the nominal *P* values obtained from edgeR and DEseq2 (*80*, *81*). The combined *P* value was adjusted using the Benjamini-Hochberg test, which uses the false discovery rate approach. The adjusted *P* value was subsequently used to rank all the DEGs obtained. A *P* value < 0.05 and a log_2_FC < −1 or > 1 were considered to indicate statistical significance. RNA-seq data have been deposited in the Gene Expression Omnibus (GEO) database (accession numbers: GSE115528 for the Δ*tasA* and Δ*calY* mutants,and GSE259377 for the Δ*capP* mutant) and are publicly available.

### TEM and immunochemistry studies

To investigate the location of the proteins within the ECM using TEM, we followed a protocol that was previously established and described, with several modifications (*13*, *30*, *37*). For TasA and CalY immunodetection, the strains were cultured in multiwell plates at 28°C for 72 hours. Alternatively, for the localization of CapP or the N-domain within *B. cereus* cells, the strains corresponding to Δ*capP* containing the plasmids pUTE657-*capP-6xHis* or pUTE657-*N-domain-6xHis* were grown in multiwell plates with TY medium supplemented with a 10 μM IPTG solution. The biofilm mass was subsequently harvested in 1 ml of PBS after 48 hours.

Then, 20 μl of each sample was applied to a copper grid and incubated for 2 hours. The grids were subsequently washed with PBS and incubated for 5 min to remove any excess bacterial cells. The cells that adhered to the grid were fixed using a 2% paraformaldehyde solution diluted in PBS and incubated for 10 min. The grids were washed again with PBS for 5 min and then blocked for 30 min using Pierce Protein-Free T20 (TBS, tris-buffered saline) blocking buffer (Thermo Fisher Scientific). The samples were then incubated for 2 hours with the primary antibody diluted 1:100 in blocking buffer. Specific primary monoclonal antibodies against TasA (KGISAGKSDKFK) and CalY (QSEPVYTETTLAD) were used to detect TasA and CalY, respectively. For the immunolocation of CapP-His and N-domain_39–190_-His, we used the primary polyclonal antibody anti–6-His produced in rabbits (Merck, SAB4301134). Subsequently, the grids were washed twice with TBS-T (tris-buffered saline with 0.05% Tween 20), each time with a 5-min incubation. The samples were incubated for 1 hour with a goat anti-rabbit 10/20-nm gold-conjugated secondary antibody (Ted Pella, Redding, CA, USA, 17010 for 10 nm and 17020 for 20 nm) at a dilution of 1:100 in blocking buffer. Next, the grids were subjected to two additional washes with TBS-T, each lasting 5 min. The samples were fixed with 2.5% glutaraldehyde for 10 min. The samples were washed with Milli-Q water for 5 min and subsequently negatively stained with a 1% uranyl acetate solution. Last, the grids were rinsed with a single drop of water and dried under dark conditions. As a negative control to check the specificity of the 20-nm gold secondary antibody, we performed the same protocol, but instead of adding the primary antibody, we added blocking buffer at that step.

The samples were examined using a Thermo Fisher Scientific Tecnai G^2^ 20 TWIN transmission electron microscope at an accelerating voltage of 120 kV. The final images were taken using an Olympus Veleta side-mounted charge-coupled device (CCD) with a resolution of 2000 by 20000 megapixels (Mpx).

### Cellular fractionation

For protein immunodetection by Western blotting, the samples were fractionated into three components: the extracellular medium, cell wall–associated proteins, and cellular contents (membrane and cytosol). Preliminary studies of CapP and the N-domain were conducted through cellular fractionation using the Δ*capP* strain harboring the plasmid pUTE657-*capP*-6xHis or pUTE657-N*-domain*-6xHis, respectively. The strains were subsequently grown in TY media with agitation at 180 rpm at 28°C. When the cultures reached an OD_600_ of 0.4, 10 μM IPTG was added to each sample, and the cultures were incubated for 4 hours.

All the samples were subjected to the following protocol. The samples were centrifuged at 6000*g* for 5 min, after which the supernatant was passed through a 0.45-μm polyethersulfone (PES) filter and retained as the extracellular medium fraction. The pellets were resuspended in 10 ml of PBS containing lysozyme (100 μg/ml) and incubated for 2 hours at 37°C. Subsequently, the samples were centrifuged at 9000*g* for 20 min at 4°C, after which the resulting supernatant and the pellet were retained as the cell wall and cellular fractions, respectively. The cellular fraction was then resuspended in a small volume of PBS. Last, all the samples were precipitated with trichloroacetic acid to a final concentration of 10%. The mixture was then incubated on ice for 1 hour. Next, the precipitated proteins were centrifuged at 13,000*g* for 20 min at 4°C, and the resulting pellets were washed twice with 1 ml of acetone. Last, the samples were dried at 37°C for 5 min, resuspended in 1× Laemmli buffer (Bio-Rad), and loaded onto a 12% SDS-PAGE gel for the immunodetection assay.

### Polyacrylamide gels and Western blotting

The samples were diluted in 1× Laemmli buffer (Bio-Rad) and then heated at 100°C for 5 min. The proteins were separated by 12% SDS-PAGE with the Spectra molecular weight marker (Thermo Fisher Scientific) and subsequently transferred to a polyvinylidene difluoride (PVDF) membrane (Bio-Rad) using the Trans-Blot Turbo Transfer System (Bio-Rad) at 25 V for 30 min. Next, the membrane was blocked for 1 hour using 5% nonfat milk diluted in 50 mM tris-HCl [150 mM NaCl (pH 7.5)] containing 0.1% Tween 20 (TBS-T). The membrane was then incubated with the primary antibody in a solution of 3% nonfat milk in TBS-T. The membrane was washed three times with TBS-T, with 10 min of incubation between each wash. Next, the membrane was incubated with the secondary antibody against rabbit immunoglobulin G (IgG) conjugated to horseradish peroxidase (Bio-Rad, 1706515) for 2 hours at a concentration of 1:3000 and diluted in TBS-T. Following the incubation, the membrane was washed twice with TBS-T and once with TBS. Last, for immunodetection, the membranes were exposed to Pierce ECL Western blotting Substrate (Thermo Fisher Scientific).

For the immunodetection of CapP-His or N-domain_39–190_-His, an anti-6×His primary antibody (Merck, SAB4301134), produced in rabbits, was used at a 1:2500 dilution. For TasA and CalY, monoclonal primary antibodies were used (anti-TasA [KGISAGKSDKFK] and anti-CalY [QSEPVYETTLAD]; NovoPro, Vivitek, Hoofddorp, The Netherlands) at a 1:10,000 dilution.

To study the molecular weight of the oligomers formed by CapP-His and N-domain_39–190_-His under native conditions, the proteins were diluted in native sample buffer (Bio-Rad), loaded onto polyacrylamide gels (Any kD Mini-PROTEAN TGX Precast Protein Gels, Bio-Rad), and analyzed using the NativeMark (Invitrogen) marker as a reference. The native gels were run in buffer containing 25 mM tris and 192 mM glycine at a constant voltage of 200 V. To visualize the proteins, the gels were stained with Coomassie brilliant blue.

### Immunolocalization studies by fluorescence microscopy

For the immunofluorescence experiments, the samples were grown as previously described for TEM studies. Then, 150 μl of each sample was added to well slides coated with 0.1% poly-l-lysine (Sigma-Aldrich) and incubated for 2 hours to facilitate bacterial adhesion to the substrate. The sample was then removed, and the bacteria that had attached to the slide surface were fixed with fixation buffer (3% paraformaldehyde and 0.1% glutaraldehyde diluted in PBS) for 10 min. Briefly, the wells were washed twice with PBS, and the samples were incubated for 1 hour with blocking buffer [3% (m/v) BSA and 0.2% (v/v) Triton X-100 in PBS]. Then, the buffer was removed, and the wells were incubated for 3 hours with the primary antibody at a concentration of 1:100 diluted in blocking buffer. The primary antibodies used for visualization by TEM were also used for this experiment. The wells were then rinsed three times with washing buffer [0.2% (m/v) BSA and 0.05% (v/v) Triton X-100 in PBS], and each incubation lasted for 5 min. Next, the wells were incubated for 2 hours with the secondary antibody goat anti-rabbit IgG-Atto488 (manually labeled) at a dilution of 1:400 in blocking buffer. The samples were washed once with washing buffer and twice with PBS, for an incubation period of 5 min each. The immunostainings were fixed for 5 min using fixation buffer, and the wells were subsequently rinsed with PBS three times. To detect the cell wall, the slide was incubated with WGA labeled with Alexa Fluor 647 (Thermo Fisher Scientific) for 20 min at a dilution of 1:100 in PBS. Last, the bacterial DNA was stained for 20 min using Hoechst at a dilution of 1:1000. As a negative control, immunostaining was performed without incubation with the primary antibody.

The immunostaining was visualized using CLSM. For the fluorescence corresponding to Atto-488 visualization, we used an excitation wavelength of 488 nm and detected the emission between 497 and 572 nm. The signal corresponding to Alexa Fluor 647 was visualized with an excitation wavelength of 561 nm, and emission was detected between 576 and 686 nm. The Hoechst signal was visualized independently using a specific dichroic filter.

For colocalization studies of CapP-His-CalY/TasA, the same immunostaining protocol was followed with a few modifications. First, CapP-His was labeled using the anti–6-His antibody produced in rabbits (Merck, SAB4301134), followed by goat anti-rabbit antibody conjugated to Atto-488 as the primary and secondary antibodies, respectively. After three washes were performed with washing buffer, the samples were treated with quenching solution (0.1 M glycine in PBS) for 10 min. Next, TasA or CalY was detected using the following specific primary antibodies: anti-TasA and anti-CalY, which were produced in rabbit, followed by anti-rabbit conjugated to Alexa Fluor 647 produced in goat as the secondary antibody. Following the final steps, the samples were fixed, stained with Hoechst, and lastly visualized via CLSM, as described previously.

The colocalization images were analyzed using the software ImageJ and the colocalization threshold plugin. The region of interest was defined for the region corresponding to Alexa Fluor 647, and both Pearson’s and Mander’s coefficients were calculated (*82*). As a negative control, the band corresponding to Alexa Fluor 647 overlapped with itself, resulting in 100% colocalization.

### Protein expression and purification

CapP-His and N-domain_39–190_-His were purified under native conditions. For the heterologous expression of CapP, the plasmid pET24b-*capP-6xHis* was transformed into *E. coli* BL21 (DE3) (Merck, Kenilworth, NJ, USA), and colonies were selected with kanamycin (50 μg/ml). The plasmid pET24-*N-domain*_39–190_-6XHis was subsequently transformed into *E. coli* Lemo21 (DE3) pLysS (New England Biolabs, USA) for N-domain_39–190_-His expression, after which colonies were selected with kanamycin (50 μg/ml) and chloramphenicol (5 μg/ml). The following steps were performed for both proteins. A single fresh colony was picked and cultured in 10 ml of LB supplemented with the corresponding antibiotics mentioned previously. The preculture was incubated under shaking conditions at 200 rpm for 6 hours at 37°C. A total of 10% (v/v) of the precultures were inoculated into 1 liter of LB supplemented with the corresponding antibiotics. The culture was incubated at 37°C with agitation at 200 rpm until the OD_600_ reached 0.6. For CapP-His, the culture was induced with 400 μM IPTG and incubated for 16 hours at 28°C with agitation at 200 rpm. Otherwise, the culture corresponding to the overexpression of N-domain_39–190_-His was induced with 400 μM IPTG and 100 μM rhamnose and incubated for 3 hours at 28°C with agitation at 200 rpm. Then, the cells were harvested (6000*g*, 30 min at 4°C, JLA 8.1 rotor, Beckman Coulter, Brea, CA, USA), and the pellets were frozen at −80°C until use.

The pellets were resuspended in 18 ml of buffer A [20 mM Na_2_PO_4_, 500 mM NaCl, and 20 mM imidazole (pH 8)] with cOmplete EDTA-free Protease Inhibitor Cocktail (Roche) and CelLytic B Cell Lysis reagent (Merck). The lysates were incubated for 30 min with agitation at 100 rpm at room temperature. The samples were sonicated using a Branson 450 digital sonifier on ice with three pulses for 45 s at 40% amplitude and then centrifuged at 15,000*g* for 60 min at 4°C (F34-6-38 rotor; Eppendorf, Hamburg, Germany). The supernatant was loaded onto a HisTrap HP 5 ml column (GE Healthcare) and purified using an AKTA Start FPLC system (GE Healthcare). The column was equilibrated with buffer A, and after sample loading, it was washed with the same buffer. Next, the proteins were eluted using a linear gradient of buffer B [20 mM Na_2_PO_4_, 500 mM NaCl, and 500 mM imidazole (pH 8)]. Since the elution mixture was not completely pure, the sample was loaded onto a HiLoad 16/600 Superdex 75 pg column (Cytiva), and the pure protein was subsequently eluted in buffer containing 20 mM tris and 50 mM NaCl (pH 7.5). The elution fractions were loaded onto a 12% tris-tricine SDS-PAGE gel, and the samples containing the purified protein were concentrated using Amicon Ultra15 centrifugal filter units (Millipore) with a 3-kDa cutoff. Subsequently, the purified fractions were separated via 12% SDS-PAGE to assess their purity, which was determined through Coomassie blue staining. Last, the gel bands were analyzed using tandem mass spectrometry. CalY and TasA were purified via heterologous expression in *E. coli* BL21 (DE3) pLysS and from inclusion bodies under denaturing conditions following the protocol established by El Mammeri *et al.* (*37*).

### Far-Western blot

For the assay, TasA—purified in vitro via heterologous expression in *E. coli* and diluted in 20 mM tris and 50 mM NaCl (pH 7.5)—was used as the prey protein. Biofilm samples from Δ*tasA* and Δ*calY* strains grown for 72 hours were collected and resuspended in PBS. Cells were lysed with lysozyme (50 μg/ml) and a protease inhibitor cocktail (Roche), followed by incubation at 37°C for 30 min with agitation at 100 rpm. Cell lysates and purified TasA were loaded onto a 12% SDS-PAGE gel. BSA was used as a negative control in place of the prey protein. A total of 20 μg of each protein was loaded. Proteins were transferred to a PVDF membrane using the Trans-Blot Turbo transfer system (Bio-Rad).

To denature and renature the proteins, the membrane was incubated in AC buffer [100 mM NaCl, 20 mM tris (pH 7.5), 0.5 mM EDTA, 10% glycerol, 0.1% Tween 20, 2% skim milk powder, and 1 mM dithiothreitol (DTT)] containing decreasing concentrations of guanidine HCl (6, 3, 1, 0.1, and 0 M). The membrane was then blocked with 5% milk in PBST (phosphate-buffered saline with 0.05% Tween 20) for 1 hour at room temperature. Next, it was incubated overnight at 4°C with the purified bait protein (200 μg/ml) in AC buffer without guanidine HCl. After three 10-min washes with PBST, the membrane was incubated overnight at 4°C with a rabbit anti-CalY primary antibody (NovoPro; Vivitek, Hoofddorp, The Netherlands) diluted 1:3000 in 3% milk in PBST. Following another series of three 10-min PBST washes, the membrane was incubated for 2 hours at room temperature with a horseradish peroxidase–conjugated anti-rabbit IgG secondary antibody (Bio-Rad). Last, the blot was washed three times with PBST and once with PBS for 5 min. Chemiluminescent detection was performed using Pierce ECL Western Blotting Substrate (Thermo Fisher Scientific).

### Pull-down assay

To study the interaction of TasA with CalY, immunoprecipitation was performed in batch. A 2-ml tube containing 300 μl of Ni-NTA resin (Sigma-Aldrich) was centrifuged for 30 s at 5000*g*. The supernatant was discarded, and the resin was washed with 600 μl of equilibration buffer, followed by a 5-min incubation at room temperature. After centrifugation, the resin was incubated for 1 hour at 4°C with 50 μM polyhistidine-tagged TasA [diluted in 20 mM tris and 50 mM NaCl (pH 7.5)] under agitation at 100 rpm to allow binding. Biofilm from the Δ*tasA* mutant strain, grown for 72 hours in a 24-well plate, was carefully resuspended in PBS by pipetting, passed 10 times through a syringe to disrupt potential bacterial clumps, and then centrifuged at 12,000*g* for 3 min. The resulting pellet was frozen at −20°C for at least 30 min and then resuspended in 1 ml of PBS containing a protease inhibitor cocktail (Roche) and lysozyme (50 μg/ml). The lysate was incubated at 37°C for 30 min and then added to the resin containing immobilized TasA. The mixture was incubated for 3 hours with agitation at 100 rpm, followed by centrifugation at 5000*g* for 1 min. The supernatant was collected as the flow-through. The resin was washed once with 600 μl of equilibration buffer [20 mM tris and 50 mM NaCl (pH 7.5)] and incubated for 5 min with agitation at 100 rpm. After centrifugation, it was washed three times with 600 μl of washing buffer [20 mM tris, 50 mM NaCl, and 50 mM imidazole (pH 7.5)], with 5-min incubations between each wash. Samples from each wash step were collected for SDS-PAGE analysis. To elute bound proteins, 200 μl of elution buffer [20 mM tris, 50 mM NaCl, and 500 mM imidazole (pH 7.5)] was added to the resin and incubated for 10 min. After centrifugation, the eluted fraction and all previously collected samples were analyzed by SDS-PAGE followed by Coomassie staining and Western blotting. For immunodetection of TasA and CalY, the same membrane was reused following a stripping step to remove previously bound antibodies. The membrane was incubated with stripping buffer [0.1 M tris-HCl (pH 6.8), 2% SDS, and 0.1 M β-mercaptoethanol] at 50°C for 45 min with agitation at 100 rpm. It was then washed with water at least three times, with 10-min incubations and agitation at 100 rpm between washes, followed by incubation in TBS-T for 15 min and reblocking before the second round of immunodetection.

### Tandem mass spectrometry analysis of protein bands

The SDS-PAGE gel bands that corresponded to the protein purification and proteinase K digestion experiments were subjected to analysis via tandem mass spectrometry using a nanoion trap system [high-performance liquid chromatography (HPLC)–electrospray ionization–tandem mass spectrometry]. The bands were cut and destained using a mixture of 50% acetonitrile (ACN) and 25 mM ammonium bicarbonate. Then, the samples were dehydrated and dried using ACN. Disulfide bridges were reduced with 10 mM DTT diluted in 50 mM ammonium bicarbonate, followed by incubation at 56°C for 30 min. Subsequently, the excess DTT was removed, and the cysteine residues were carbamidomethylated using 55 mM iodoacetamide diluted in 50 mM ammonium bicarbonate for 20 min at room temperature in the dark. The gel bands were then dehydrated again, and the proteins were digested using trypsin (10 ng/μl; Promega) at 30°C overnight. To extract the peptides from the gel, the samples were incubated with a solution of 0.1% ACN/formic acid (FA) for 30 min at room temperature. To eliminate ACN and ammonium bicarbonate, the samples were dried using a SpeedVac. Next, the samples were resuspended in a solution containing 0.1% FA, ultrasonicated for 3 min, and centrifuged at 13,000*g* for 5 min. Subsequently, the samples were purified and concentrated using C18 ZipTip (Merck) following the instructions supplied by the manufacturer. Last, the samples were injected into an Easy nLC 1200 UHPLC (Ultra-High-Performance Liquid Chromatography) system, which was coupled to a Q Exactive HF-X Hybrid Quadrupole-Orbitrap mass spectrometer (Thermo Fisher Scientific). The software versions used for acquisition and analysis were Tune 2.9 and Xcalibur 4.1.31.9. The mobile phases used in the HPLC consisted of (i) buffer A, which contained 0.1% FA dissolved in water, and (ii) buffer B, which contained 0.1% FA dissolved in 80% ACN. Peptides were loaded onto a precolumn (Acclaim PepMap 100, 75 μm by 2 cm; C18, 3 μm; 100 A; Thermo Fisher Scientific) at a flow rate of 20 μl/min, while elution was performed using a 50-cm analytical column (PepMap RSLC C18, 2 μm; 100 A, 75 μm by 50 cm; Thermo Fisher Scientific). Elution was carried out through a gradient concentration over 60 min, transitioning from 5 to 20% buffer B. This was followed by a 5-min gradient from 20 to 32% buffer B, resulting in a 10-min elution with 95% buffer B. Then, the column was equilibrated with 5% buffer B using a constant flow rate of 300 μl/min. Before sample analysis, an external calibration was performed using LTQ Velos ESI Positive Ion Calibration Solution (Pierce, IL, USA), along with internal calibration using the polysiloxane ion signal at mass/charge ratio (*m/z*) 445.120024 obtained from ambient air. The MS1 scans were conducted within a *m/z* range of 375 to 1.600 at a resolution of 120.000. Using a data-dependent acquisition strategy, the 15 most intense precursor ions with a charge ranging from +2 to +5 within a window of 1.2 *m/z* were selected from fragmentation to generate the corresponding MS2 spectra. The fragmentation ions were generated through high-energy collision-induced dissociation with an initial mass set at 110 *m/z* and subsequently detected using a mass-analyzed Orbitrap at a resolution of 30.000. The dynamic exclusion for the selected ions was set to 30 s, and the maximum accumulation times for MS1 and MS2 were 50 and 70 ms, respectively. Last, for protein sequence identification, the raw data were analyzed using Proteome Discoverer 2.4 (Thermo Fisher Scientific) with the Sequest HT search tool with mass tolerance parameters of 10 ppm and 0.02 Da for precursor and fragment ions, respectively.

### Dynamic light scattering experiments

The samples corresponding to the elution peaks obtained by size exclusion chromatography for CapP-His and N-domain_39–190_-His were filtered through a 0.46-μm syringe filter. The size measurements were performed using a Malvern Zetasizer Nano ZS (Malvern Panalytical, Malvern, United Kingdom) with a laser wavelength of 632.8 nm as the excitation source and a 1-cm pathway polystyrene cuvette as the sample holder. A total volume of 1 ml of the protein solution was transferred to the cuvette, and the dynamic light scattering signal was measured with a count rate ranging from 100 to 230 kilo counts per second (kcps), depending on the sample. Data collection and subsequent analysis were conducted using Zetasizer software v.6.34 (Malvern Panalytical).

### Transmission electron microscopy

To study the morphology of CapP-His, N-domain_39–190_-His, and CalY, the samples were subjected to twofold serial dilutions and incubated on copper grids for 2 min. Then, the samples were stained with a 2% uranyl acetate solution for 1 min and dried under dark conditions.

The samples were examined using a Thermo Fisher Scientific Tecnai G^2^ 20 TWIN transmission electron microscope at an accelerating voltage of 120 kV. The final images were captured using an Olympus Veleta side-mounted CCD with a resolution of 2000 by 2000 Mpx.

### ThT assays

The polymerization kinetics were studied using ThT staining. The experiment was conducted in a 96-well plate, and 40 μM ThT was added to each well at a specific concentration, after which the mixture was diluted in buffer containing 20 mM tris and 50 mM NaCl at pH 7.4. The polymerization kinetics of CalY were assayed at 40 μM using low concentrations of CapP-His and N-domain_39–190_-His (2 and 10 μM).

The plates were incubated at 37°C in a plate reader and shaken at 100 rpm before each measurement (Tecan Infinite M1000 Pro; Tecan, Männedorf, Switzerland). The fluorescence intensity was monitored every 30 min through the bottom of the plate using an excitation wavelength of 440 nm and an emission wavelength of 480 nm. All measurements were recorded in triplicate.

### Sequence analysis by different bioinformatic tools

The primary sequence of CapP (BC1280) was obtained from UniProt (https://uniprot.org/) with the accession number Q81GC7. SignalP5.0 was used to predict the cleavage site for the signal peptidase (*83*). The hydrophobicity was analyzed using the ProtScale tool provided by the ExPASy server, and the prediction of disordered regions was conducted using FoldUnfold with default parameters (*42*, *50*). Putative aggregation-prone regions were predicted using the following bioinformatic tools: AmylPred2, FoldAmyloid, MetAmyl, PASTA2.0, and TANGO (*45*–*49*).

### In vitro assembly of oligomers

CapP-His and N-domain_39–190_-His were diluted to a final concentration of 50 μM in buffer containing 20 mM tris and 50 mM NaCl at pH 7.5. The oligomers self-assembled under agitation at 200 rpm for 2 weeks at 37°C and a speed of 200 rpm. The aggregates were then ultracentrifuged (40,000 rpm speed, 5 hours, 18°C, TLA 120.1 rotor; Beckman Coulter), and the resulting pellets were analyzed using x-ray diffraction, ATR-FTIR, and SSNMR.

### X-ray diffraction measurements

Filament diffraction patterns were obtained at 4°C using a Rigaku FR-X rotating anode x-ray generator (Rigaku, Tokyo, Japan) equipped with an EIGER 1 M hybrid pixel detector (Dectris, Baden, Switzerland) at the copper wavelength. The concentrated hydrated samples were mounted in MicroLoops from Mitegen (Ithaca, NY, USA) on a goniometer head under cold nitrogen flow. Each diffraction pattern represents a 360° rotation along the φ axis, with an exposure time of 720 s. No correction, such as smooth filtering or baseline correction, was applied to the data. WinPLOTR (https://cdifx.univ-rennes1.fr/winplotr/winplotr.htm) was used for converting *q* values to reticular distance (*d*) values before plotting.

### Proteinase K digestion

A 22.5-μg solution of aggregated N-domain_39–190_-His was treated for 45 min at 37°C with proteinase K (3 μg/ml) in 20 mM tris and 50 mM NaCl at pH 7.5. The proteinase activity was stopped using 5 mM phenylmethylsulfonyl fluoride. The samples were mixed with an equal volume of Laemmli buffer (Bio-Rad) and heated at 100°C for 5 min. Subsequently, the samples were separated using 12% SDS-PAGE, and the bands were visualized by Coomassie blue staining. The bands of interest were excised and subjected to analysis by tandem mass spectrometry.

### ATR-FTIR spectroscopy

The samples were treated under the same conditions as those used for the x-ray studies. Infrared spectra for CapP-His and N-domain_39–190_-His were recorded with an FTIR spectrophotometer (model Vertex70; Bruker) in ATR mode using a Golden Gate Single Reflection Diamond ATR System accessory (Specac). No sample preparation was needed, and spectra were recorded with an air background and 64 scans for both the background and sample measurements. The spectral resolution was set at 4 cm^–1^″. The raw data within the spectral range of the amide I region were used for estimating the total amount of secondary structure. To increase the resolution of the minimal absorbance peaks, a second derivative was calculated. Next, a deconvolution approach was used using PeakFit software to determine the contribution percentage for each type of secondary structure: parallel β sheet (1634 cm^−1^), antiparallel β sheet (1620 and 1690 cm^−1^), α helix (1658 cm^−1^), turns (1675 cm^−1^), and random coil (1646 cm^−1^), in accordance with the well-established assignments (*84*).

### Proteomic analysis by the iTRAQ method

To estimate the relative levels of TasA, CapP, and CalY during biofilm formation in the wild-type strain *B. cereus* ATCC14579, we conducted sample preprocessing and subsequent analysis following the protocols described by Caro-Astorga *et al.*, in 2020 (*32*). The MS proteomic data have been deposited to the ProteomeXchange Consortium via the Proteomics Identification (PRIDE) partner repository with the dataset identifier PXD010211.

### NMR spectroscopy

Solid-state NMR experiments were performed on a 600-MHz spectrometer (Bruker Biospin, Germany) equipped with a triple resonance 4-mm probe. Experiments were performed at 11-kHz MAS frequency at a sample temperature of 283 K. Chemical shifts were referenced with dextran sulfate sodium. For 2D ^13^C-^13^C PDSD spectra (mixing time of 50 and 200 ms), an initial cross-polarization step of 0.5 ms was used, and high-power SPINAL-64 decoupling was applied during acquisition times. 2D ^13^C-^13^C PDSD (50 ms) and ^1^H-^13^C INEPT spectra were recorded for 1 to 3 days and 6 days for the ^13^C-^13^C PDSD spectrum (200 ms). The NMR data were processed using the TOPSPIN 4.0.6 software and analyzed by CCPNMR (Collaborative Computational Project for NMR) (*85*).

### Circular dichroism

CalY (40 μM) alone or in combination with CapP-His (at 2 or 10 μM) was incubated in 10 mM sodium phosphate buffer at pH 8 for 16 hours at 37°C under agitation at 200 rpm. Circular dichroism spectra were recorded using a JASCO J-815 spectrometer, using a 0.1-cm path length cuvette in the range of 180 to 260 nm, using a 0.5-nm step and a 1-s collection time per step. The scan rate was 50 nm/min. Final spectra were obtained as the average of six scans after blank correction. The spectrometer was continuously purged with dry N_2_ gas. Circular dichroism spectra were buffer-subtracted, and the results were expressed as mean residue ellipticity. To estimate the CalY structural rearrangement, the contribution of CapP-His at each concentration was removed from the experimental spectra, and the corrected spectra were analyzed using the Dichroweb (*52*) software with the CDSSTR and CONTIN algorithms, reference set 7, and a wavelength range of 190 to 240 nm. The results from both algorithms were averaged and are shown in table S20.
