# Supplementary material for: Matrix plasticity and the molecular basis of extracellular filament assembly in Bacillus cereus
Source: Sci Adv. 2026 Apr 15;12(16):eaea1826. doi: 10.1126/sciadv.aea1826 (PMC13082335; doi:10.1126/sciadv.aea1826)
Supplement: Supplementary file 1 — Figs. S1 to S14 Tables S1 to S24 [file sciadv.aea1826_sm.pdf]

Science Advances
AAAS

Supplementary Materials for

Matrix plasticity and the molecular basis of extracellular filament assembly in Bacillus cereus

Ana Álvarez-Mena *et al.*

Corresponding author: Diego Romero, diego_romero@uma.es; Antoine Loquet, a.loquet@iecb.u-bordeaux.fr

Sci. Adv. **12**, eaea1826 (2026)
DOI: 10.1126/sciadv.aea1826

This PDF file includes:

Figs. S1 to S14
Tables S1 to S24

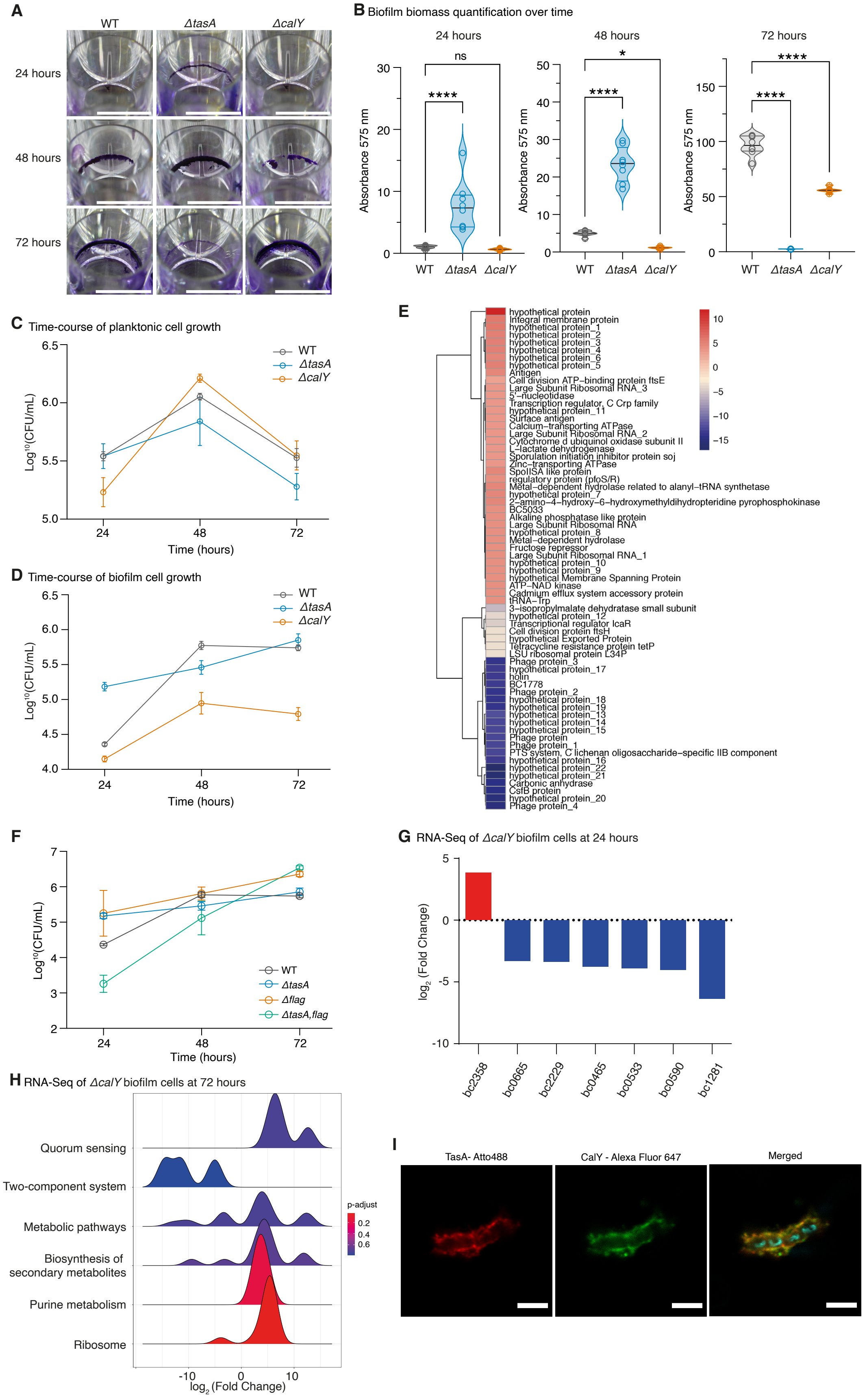

A
WT
ΔtasA
ΔcalY
24 hours
48 hours
72 hours
B Biofilm biomass quantification over time
24 hours
48 hours
72 hours
ns
*
****
Absorbance 575 nm
WT
ΔtasA
ΔcalY
C Time-course of planktonic cell growth
Log10(CFU/mL)
Time (hours)
D Time-course of biofilm cell growth
Log10(CFU/mL)
Time (hours)
E
hypothetical protein
Integral membrane protein
hypothetical protein_1
hypothetical protein_2
hypothetical protein_3
hypothetical protein_4
hypothetical protein_6
hypothetical protein_5
Antigen
Cell division ATP-binding protein ftsE
Large Subunit Ribosomal RNA_3
5'-nucleotidase
Transcription regulator, C Crp family
hypothetical protein_11
Surface antigen
Calcium-transporting ATPase
Large Subunit Ribosomal RNA_2
Cytochrome d ubiquinol oxidase subunit II
L-lactate dehydrogenase
Sporulation initiation inhibitor protein soj
Zinc-transporting ATPase
SpoIISA like protein
regulatory protein (pfoS/R)
Metal-dependent hydrolase related to alanyl-tRNA synthetase
hypothetical protein_7
2-amino-4-hydroxy-6-hydroxymethyldihydropteridine pyrophosphokinase
BC5033
Alkaline phosphatase like protein
Large Subunit Ribosomal RNA
hypothetical protein_8
Metal-dependent hydrolase
Fructose repressor
Large Subunit Ribosomal RNA_1
hypothetical protein_10
hypothetical protein_9
hypothetical Membrane Spanning Protein
ATP-NAD kinase
Cadmium efflux system accessory protein
tRNA-Trp
3-isopropylmalate dehydratase small subunit
hypothetical protein_12
Transcriptional regulator IcaR
Cell division protein ftsH
hypothetical Exported Protein
Tetracycline resistance protein tetP
LSU ribosomal protein L34P
Phage protein_3
hypothetical protein_17
holin
BC1778
Phage protein_2
hypothetical protein_18
hypothetical protein_19
hypothetical protein_13
hypothetical protein_14
hypothetical protein_15
Phage protein
Phage protein_1
PTS system, C lichenan oligosaccharide-specific IIB component
hypothetical protein_16
hypothetical protein_22
hypothetical protein_21
Carbonic anhydrase
CsfB protein
hypothetical protein_20
Phage protein_4
F
Log10(CFU/mL)
Time (hours)
WT
ΔtasA
Δflag
ΔtasA,flag
G RNA-Seq of ΔcalY biofilm cells at 24 hours
log2 (Fold Change)
bc2358
bc0665
bc2229
bc0465
bc0533
bc0590
bc1281
H RNA-Seq of ΔcalY biofilm cells at 72 hours
Quorum sensing
Two-component system
Metabolic pathways
Biosynthesis of secondary metabolites
Purine metabolism
Ribosome
p-adjust
log2 (Fold Change)
I
TasA- Atto488
CalY - Alexa Fluor 647
Merged

Fig. S1.

Deletion of *tasA* or *calY* alters biofilm formation, growth and gene expression. (A) Biofilm formation dynamics of wild-type, *ΔtasA* and *ΔcalY* strains over time, assessed by crystal violet staining of surface-adhered biomass. Scale bars: 1 cm. **(B)** Quantification of biofilm biomass for the indicated strains at each time point, measured by absorbance at 575 nm. Data represent three biological replicates, each with at least two technical replicates. Statistical significance was determined by one-way ANOVA with Dunnett's multiple comparisons test using the wild-type strain as control: 24 hours (ns, not significant; ****, $p < 0.0001$), 48 hours (*, $p = 0.0184$; ****, $p < 0.0001$), 72 hours (****, $p < 0.0001$). **(C)** Growth curves of planktonic cells over 72 hours for wild-type (grey), *ΔtasA* (blue) and *ΔcalY* (orange). Data are represented as mean ± s.d. from three biological replicates, each with three technical replicates. **(D)** Growth of biofilm-associated cells over 72 hours for the same strains. Data are represented as mean ± s.d. from three biological replicates, each with three technical replicates. **(E)** Differential gene expression in *ΔtasA* versus wild-type at 24 hours, determined by RNA-Seq. A total of 66 genes were deregulated, with 39 upregulated and 27 downregulated. **(F)** CFU counts during biofilm development for wild-type, *ΔtasA, Δflag* and *ΔtasA,flag* strains. Data represent mean ± s.d. from three biological replicates, each with three technical replicates. **(G)** RNA-Seq analysis of *ΔcalY* biofilm cells at 24 hours, showing transcriptional changes relative to wild-type. **(H)** RNA-Seq analysis of *ΔcalY* biofilm cells at 72 hours, showing transcriptional changes relative to wild-type. **(I)** CLSM images showing TasA and CalY localization by immunocytochemistry using specific primary antibodies. TasA and CalY were detected with secondary antibodies conjugated to Atto 488 and Alexa Fluor 647, respectively. These single-channel images correspond to the same field of view shown as the merged image in Fig. 2A. Scale bar, 4 µm.

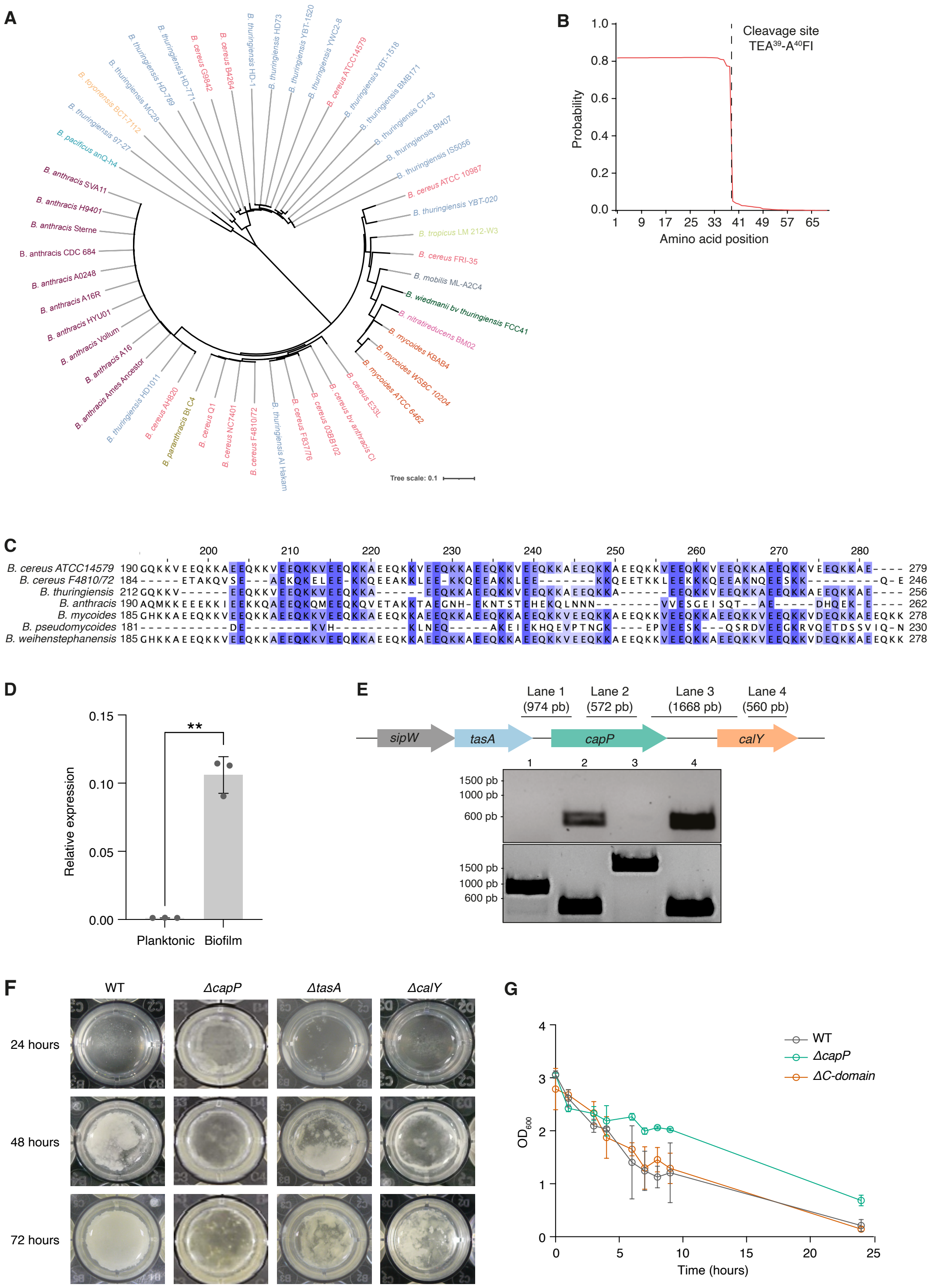

Fig. S2.

CapP is conserved in the *B. cereus* group and required for biofilm formation. **(A)** Phylogenetic conservation of CapP (BC1280) across *B. cereus* group strains, using CapP from *B. cereus* ATCC14579 as reference. Homologous sequences were retrieved by BLASTP, aligned with Clustal Omega, and the phylogenetic tree was constructed in MEGA11 using the Neighbor-

Joining method with 1000 bootstrap replicates and the Poisson substitution model. Evolutionary distances were calculated based on this alignment. **(B)** Signal peptide prediction (SignalP5.0 (84)) for CapP, showing a potential cleavage site between residues A39 and A40. **(C)** Amino acid alignment of the CapP C-domain across strains, with conservation levels color-coded using Jalview's (87) default Percentage Identity scheme, where higher identity values are represented by darker tones and lower identity by progressively lighter tones: > 80% identity in the darkest tone, > 60% in an intermediate tone, > 40% in a pale tone and ≤ 40% in white. **(D)** Relative expression of *capP* in planktonic and biofilm cells of the wild-type strain at 48 hours. Data are mean ± s.d. from three biological replicates (**, p = 0.0055, two-tailed t-test with Welch's correction). **(E)** RT-PCR products using cDNA from wild-type cells grown for 48 hours (top) and genomic DNA as control (bottom) to verify primer functionality. The experiment was repeated three times independently with consistent results. **(F)** Biofilm formation by *B. cereus* AH187 (F4810/72) wild-type and *ΔtasA*, *ΔcalY* and *ΔcapP* mutant strains at 24, 48 and 72 hours at 28 °C. The experiment was repeated three times independently with consistent results. **(G)** Auto-aggregation kinetics of wild-type, *ΔcapP* and *ΔC-domain* strains. OD_{600} of the air-liquid interface was monitored each hour. Data represent mean ± s.d. from three biological replicates.

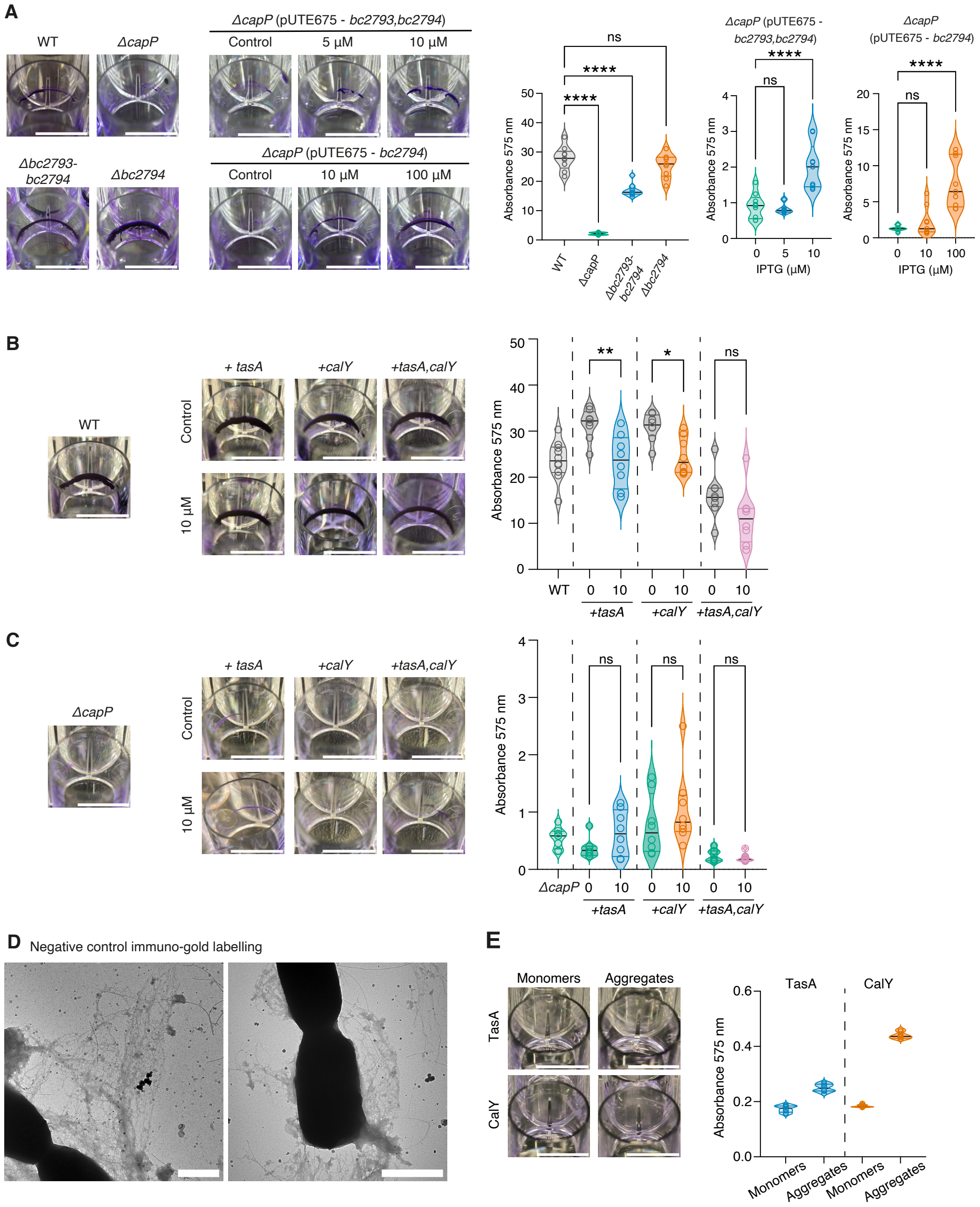

Fig. S3.

Deregulation of *tasA*, *calY*, and an uncharacterized ECF sigma factor in the *ΔcapP* mutant. **(A)** Biofilm formation after 72 hours by *ΔcapP* overexpressing *bc2793-bc2794* or *bc2794* at different IPTG concentrations, and by *Δbc2793-bc2794* or *Δbc2794* deletion mutants, compared to the wild-type and *ΔcapP* strains. Scale bars: 1 cm. Biofilms were stained with crystal violet, and biomass was quantified by absorbance at 575 nm. Individual measurements from three biological replicates, each with three technical replicates are shown. Statistical analysis was performed using one-way ANOVA with Dunnett's multiple comparison test. For the deletion mutants, wild-type strain was used as control (ns, not significant; ****, $p < 0.0001$). For *ΔcapP* overexpression, the

non-induced condition was used as control (ns, not significant; ****, $p < 0.0001$). **(B)** Biofilm phenotypes of the wild-type strain after overexpression of *tasA*, *calY*, or both genes using the pUTE657 plasmid and induction with 10 µM IPTG. As a negative control, strains carrying the plasmid were grown without IPTG. Scale bars: 1 cm. Biofilm formation was visualized by crystal violet staining, and biomass was quantified by absorbance at 575 nm. Individual measurements from three biological experiments, each with at least two technical replicates, are shown. Statistical analysis was performed using one-way ANOVA with Šídák's multiple comparisons test, using the non-induced condition as control (ns, not significant; *, $p = 0.0271$; **, $p = 0.0035$). **(C)** Same as in (B) but for the *ΔcapP* strain. No significant differences were detected. **(D)** Specificity control for nanogold immunolabeling. The wild-type strain was processed without primary antibody and stained using a goat anti-rabbit secondary antibody conjugated with 20 nm nanogold particles (1:100). Scale bar, 1 µm. **(E)** Negative control for extracellular complementation experiments. TasA and CalY were incubated in TY medium at 6 µM, in monomeric or polymerized form, without bacterial inoculation. Biofilm formation was assessed by crystal violet staining and absorbance at 575 nm. Scale bars, 1 cm. Values represent three independent experiments, each comprising three technical replicates.

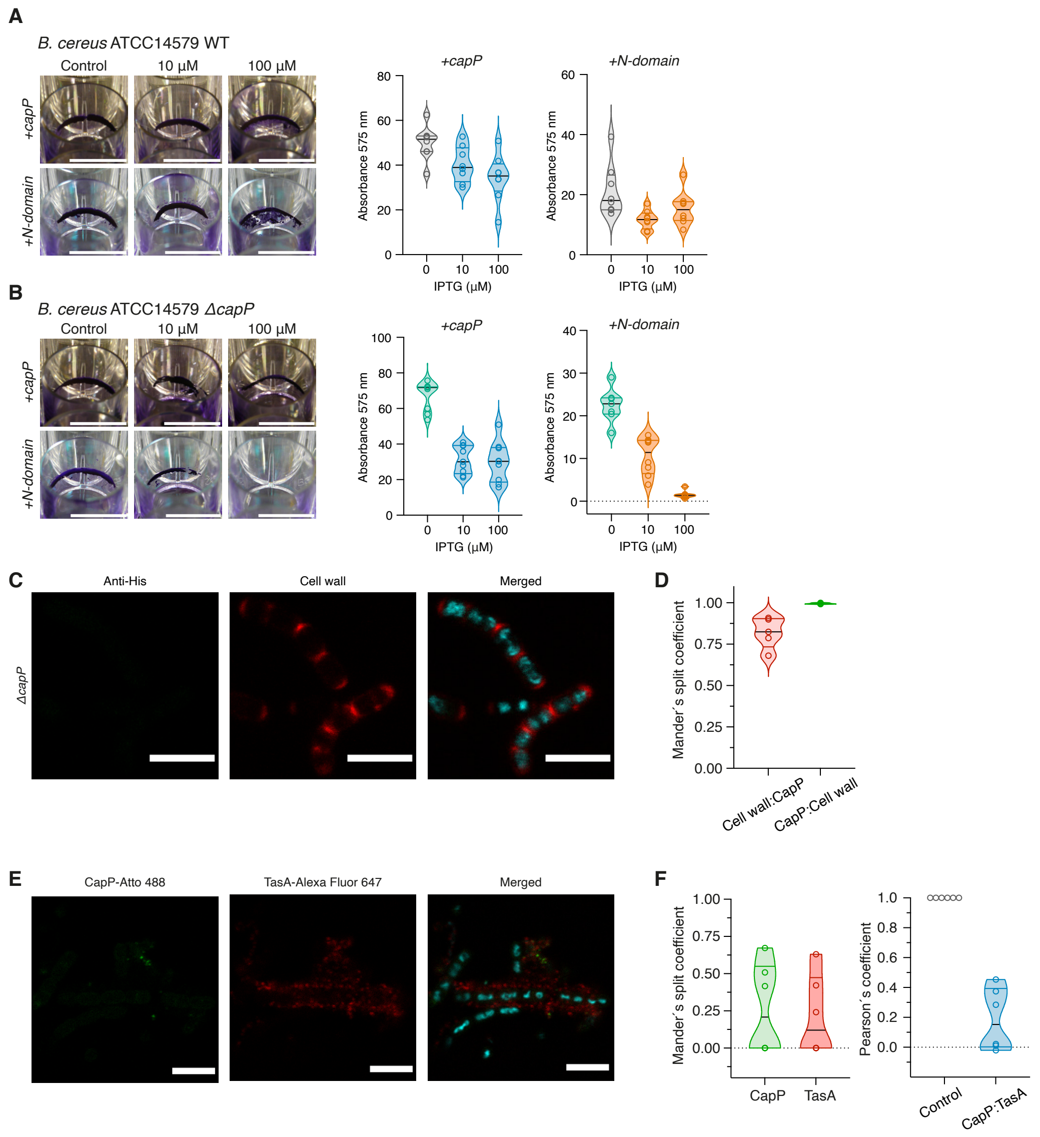

Fig. S4.

CapP overexpression restores biofilm formation in *ΔcapP* and colocalizes with CalY but not TasA. (A, B) Overexpression of full-length *capP*-His or the N-domain$_{39\text{-}190}$-His in wild-type and *ΔcapP* strains, induced with 10 or 100 µM IPTG. Biofilm biomass adhered to abiotic surfaces was quantified by crystal violet staining and absorbance measurement at 575 nm after 72 hours. Individual values from three biological experiments, each with at least two technical replicates, are shown. Scale bars: 1 cm. **(C)** Negative control for CapP-His and N-domain$_{39\text{-}190}$-His immunolocalization in *ΔcapP* biofilm samples lacking overexpression plasmids. CLSM images show staining with anti-His primary and Atto-488 conjugated secondary antibodies (green), the cell wall was labeled with WGA (red), and DNA with Hoechst (blue). Scale bar, 5 µm. **(D)** Quantification of CapP colocalization with the cell wall in biofilms using Mander's split coefficient across five CLSM fields of view. **(E)** CLSM immunolocalization of CapP (green) and TasA (red) in biofilms. CapP was detected with an anti-His primary antibody and Atto 488-conjugated secondary antibody, and TasA with a specific anti-TasA primary antibody and Alexa

Fluor 657-conjugated secondary antibody. DNA was stained with Hoechst (blue). Images were collected from three independent experiments, analyzing at least 3 fields per sample. Scale bar, 5 µm. **(F)** Colocalization analysis of CapP and TasA using Pearson's coefficient and Mander's split coefficient calculated from at least 6 fields of view across three independent experiments.

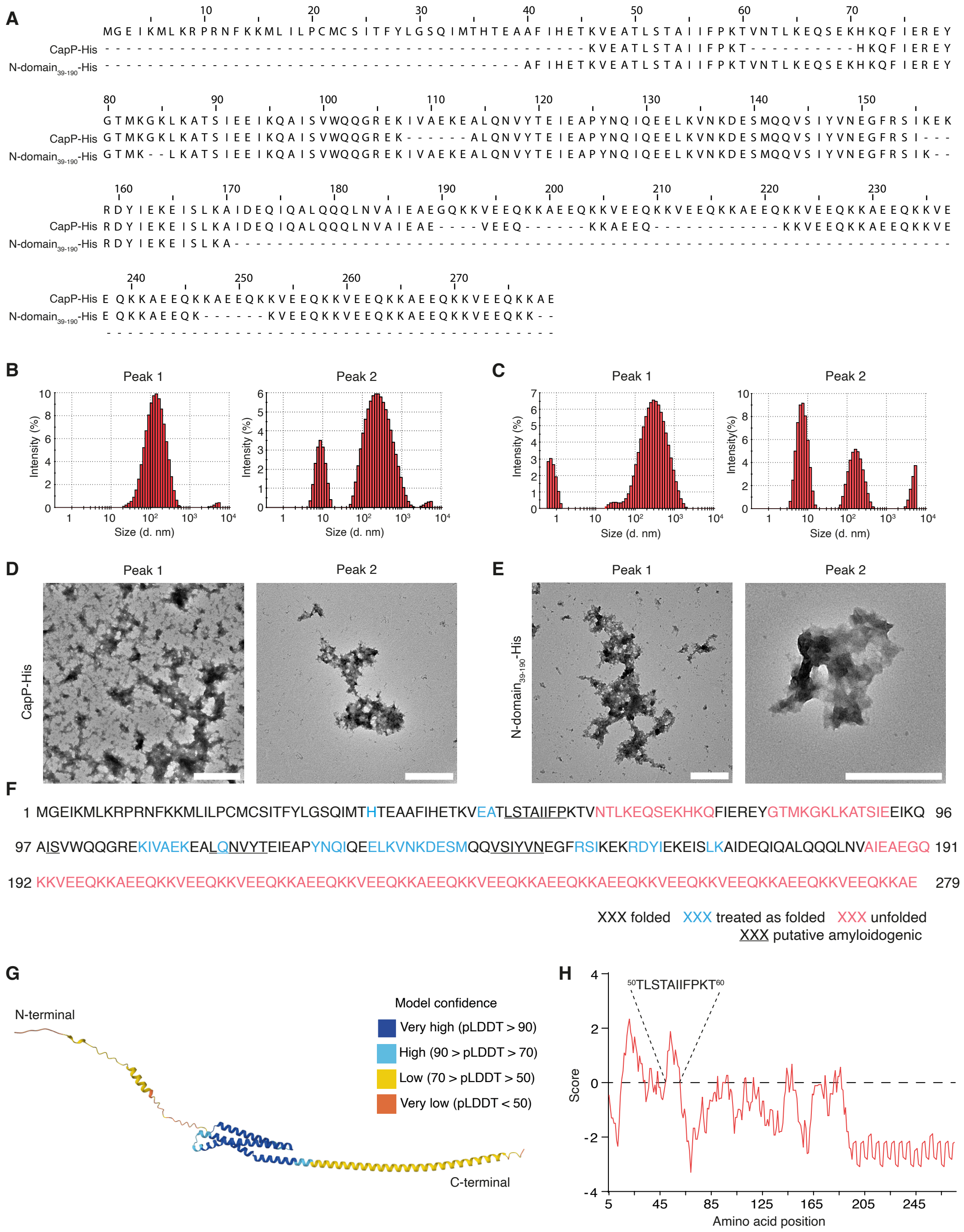

Fig. S5.

CapP-His exhibits strong oligomerization propensity in vitro, and folding is restricted to the N-terminal$_{39\text{-}190}$ domain. (A) Mass spectrometry analysis of elution fractions corresponding to peaks of CapP-His and N-domain$_{39\text{-}190}$-His. **(B, C)** Size distribution histograms from dynamic light scattering of CapP-His and N-domain$_{39\text{-}190}$-His aggregates corresponding to SEC peak 1 and 2, respectively. **(D, E)** Transmission electron micrographs of the first and second SEC peaks for CapP-His and N-domain$_{39\text{-}190}$-His. Scale bars: 1 µm for both CapP-His peaks; 1 µm for N-domain$_{39\text{-}190}$-His peak 1 and 500 nm for N-domain$_{39\text{-}190}$-His peak 2. **(F)** Predicted folding state of

CapP using FoldUnfold. Residues classified as folded (black), treated as folded (blue), or unfolded (red). Aggregation-prone regions identified by at least two independent algorithms are underlined. **(G)** AlphaFold structural model of CapP colored by pLDDT confidence score. **(H)** Hydrophobicity profile of CapP residues predicted with ProtScale (ExPASy).

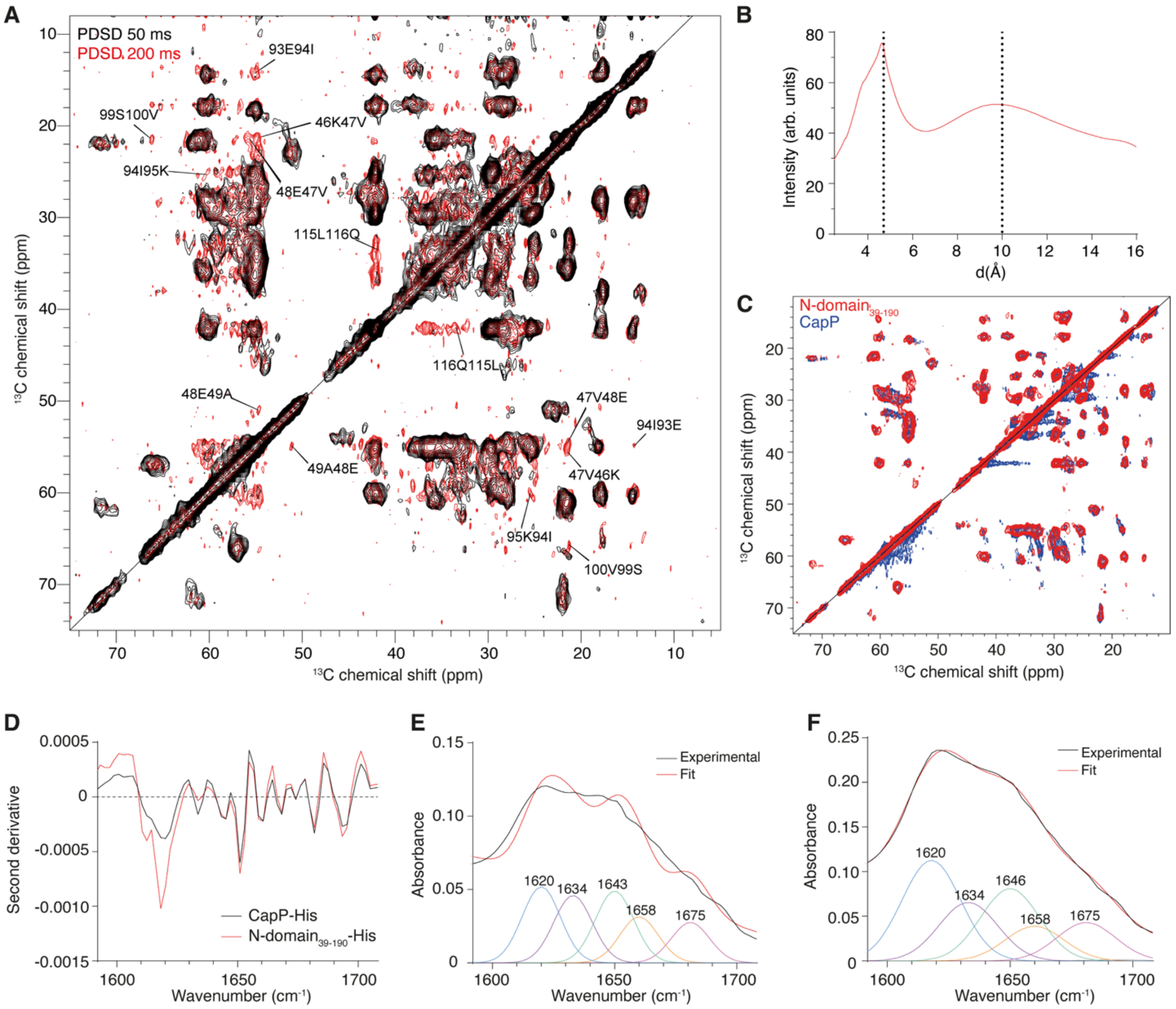

Fig. S6.

Structural features of the N-domain are conserved in full-length CapP. (A) Cross-peak assignment for inter-residue contacts based on the overlay of 2D ^{13}C-^{13}C PDSD spectra of N-domain$_{39\text{-}190}$-His recorded at mixing times of 50 ms (black) and 200 ms (red); cross-peaks corresponding to inter-residue chemical shifts are indicated. **(B)** X-ray diffraction pattern of CapP-His recorded in the 0-15 Å range. **(C)** Superimposition of 2D ^{13}C-^{13}C PDSD spectra of CapP-His (blue) and N-domain$_{39\text{-}190}$-His (red) in the amide I region. **(D)** Second derivative analysis of ATR-FTIR spectra for CapP-His (black) and N-domain$_{39\text{-}190}$-His (red) in the amide I region. **(E, F)** ATR-FTIR spectra of CapP-His (E) and N-domain$_{39\text{-}190}$-His (F) in the amide I band; contributions of individual components were determined by secondary derivative analysis followed by peak deconvolution.

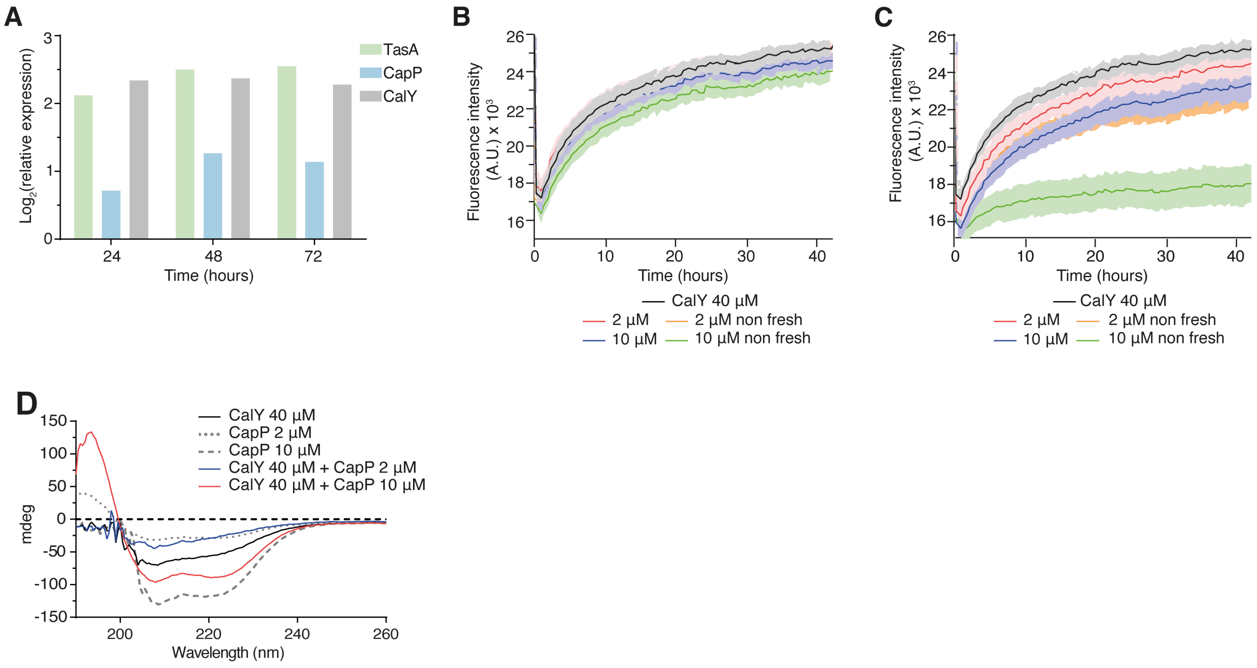

Fig. S7.

CapP levels influence CalY polymerization dynamics. **(A)** Relative levels of TasA, CapP and CalY in biofilm cells over 72 hours determined by iTRAQ analysis. **(B, C)** Thioflavin T fluorescence kinetics of CalY aggregation in the presence of freshly purified or non-freshly purified CapP-His (B) or N-domain$_{39\text{-}190}$-His (C). Results are representative of three independent experiments with three technical replicates each; error bars represent ± s.d. **(D)** Circular dichroism spectra of CalY incubated alone or with CapP-His at 2 and 10 µM for 16 hours under agitation; spectra include CapP contribution. Representative spectra are shown; experiments were repeated three times with similar results.

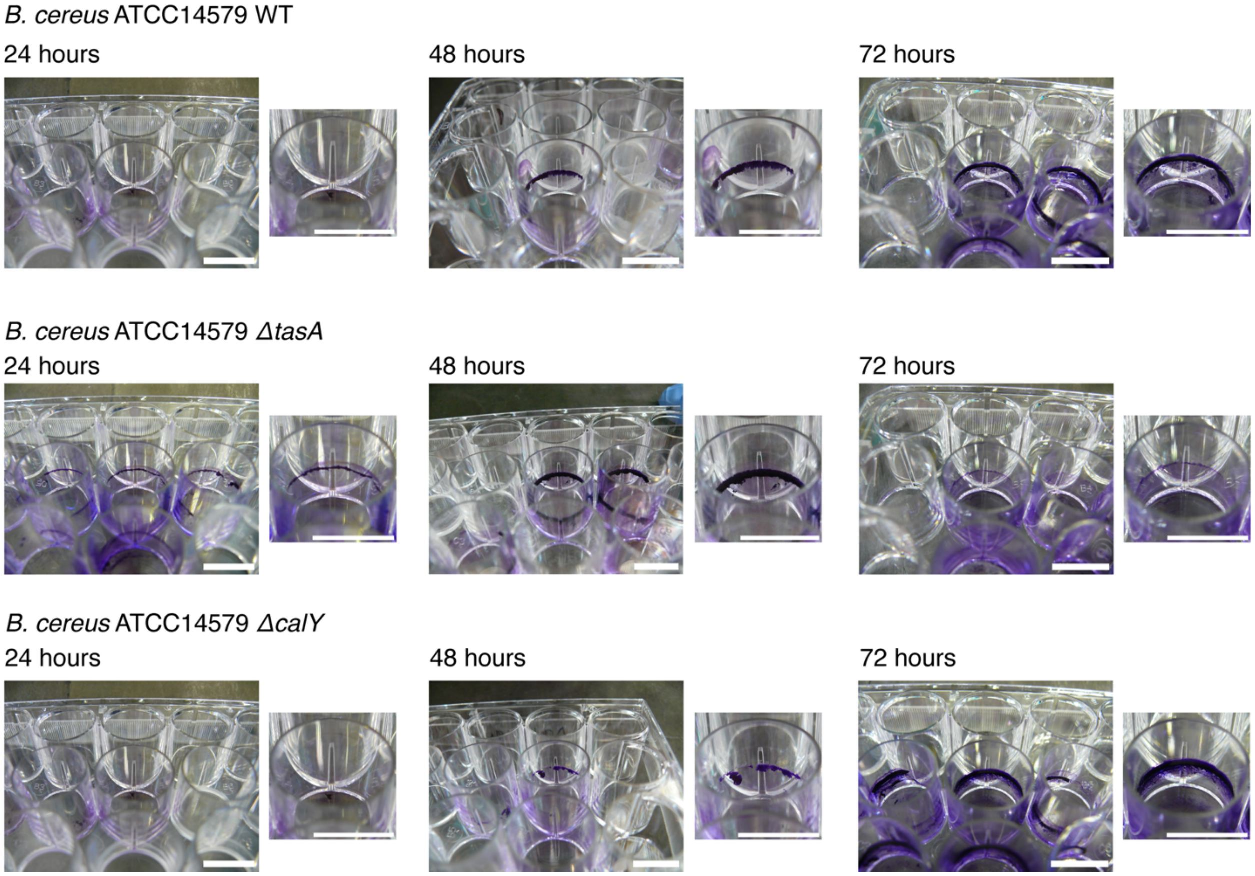

Fig. S8.

Full-plate raw data for biofilm formation dynamics of *B. cereus* wild-type, *ΔtasA* and *ΔcalY* strains. Full-plate images and corresponding cropped views used for quantification of biofilm formation dynamics in wild-type, *ΔtasA* and *ΔcalY* strains over time, assessed by crystal violet staining of surface-adhered biomass. Scale bars, 1 cm. Cropped images correspond to those shown in fig. S1A.

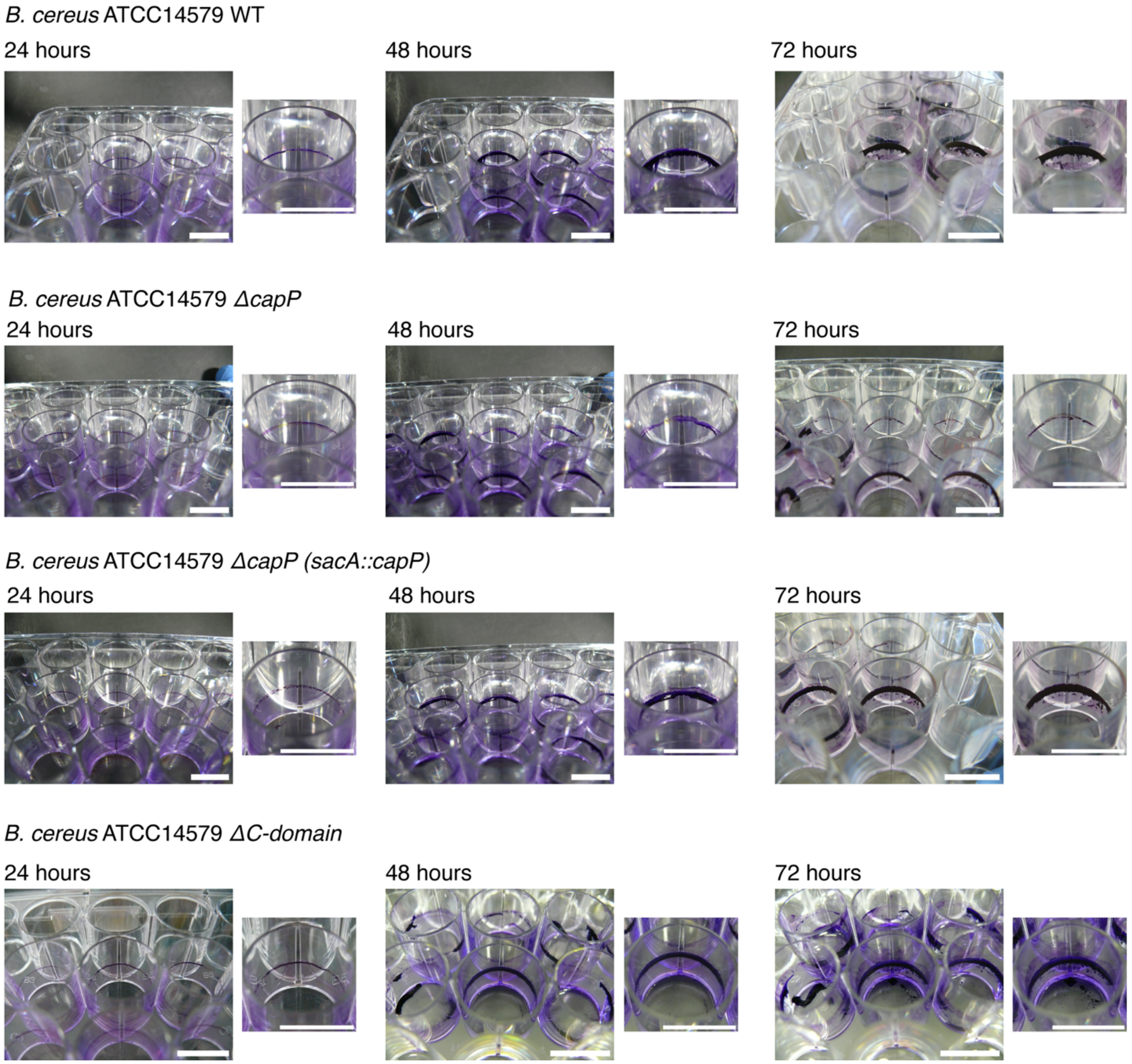

Fig. S9.

Full-plate raw data for biofilm formation dynamics of *B. cereus* wild-type, *ΔcapP*, *ΔcapP* (*sacA::capP*) and *ΔC-domain* strains. Raw full-plate images and corresponding cropped views used for the biofilm formation shown in Fig. 3B. Biofilm formation was evaluated for wild-type, *ΔcapP*, *ΔcapP* (*sacA::capP; ΔcapP* complemented by chromosomal integration of *capP* at the *sacA* neutral locus under its native promoter) and *ΔC-domain* strains at different time points using crystal violet staining of surface-adhered biomass. Scale bars, 1 cm.

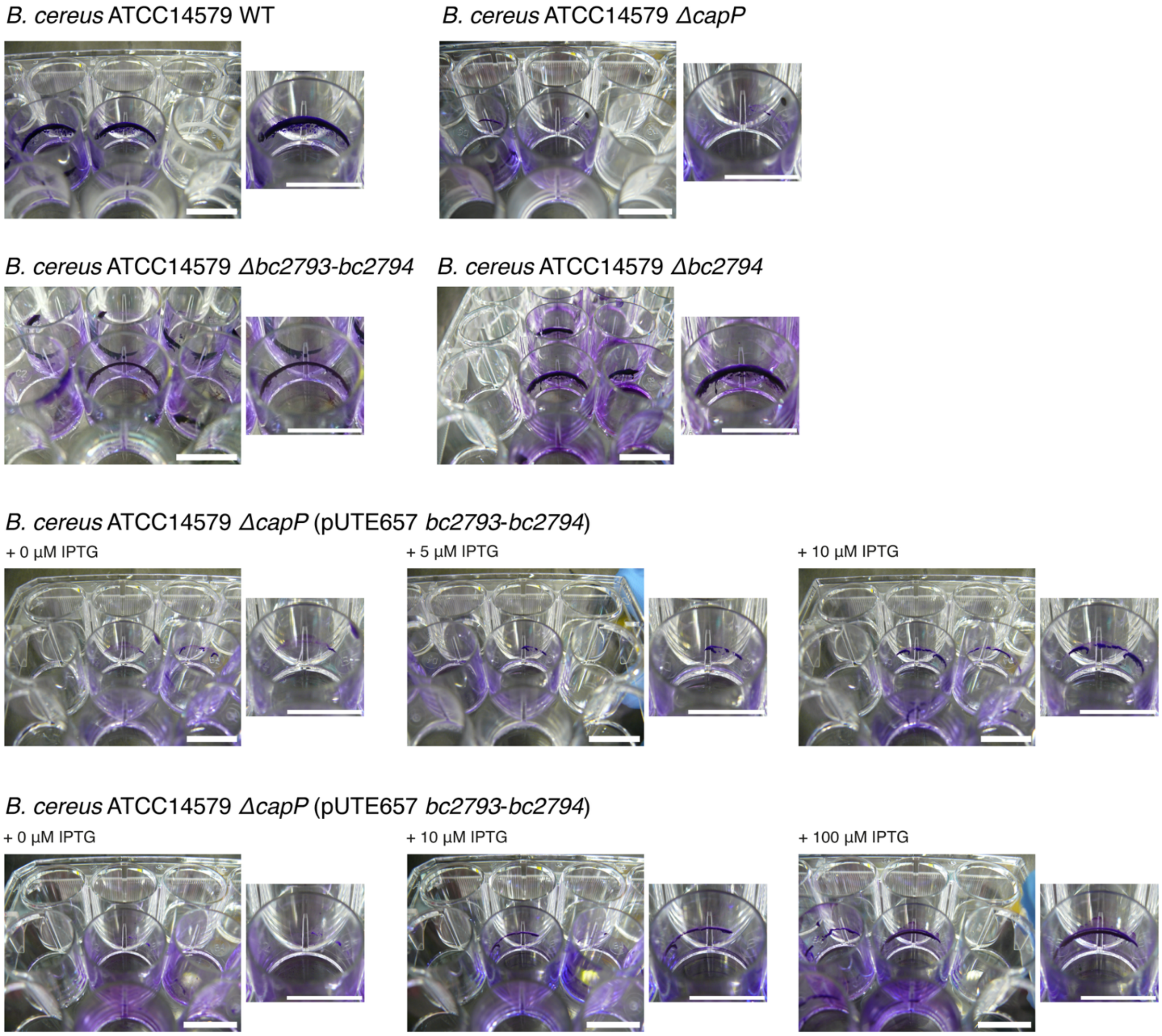

Fig. S10.

Full-plate raw data for biofilm formation of *ΔcapP* overexpression *bc2793-bc2794* or *bc2794* and corresponding deletion mutants. Raw full-plate images and corresponding cropped views used for the biofilm formation assay shown in fig. S3A. Biofilm formation after 72 hours was assessed for *ΔcapP* strains overexpressing either *bc2793-bc2794* or *bc2794* at different IPTG concentrations, as well as for the *Δbc2793-bc2794* and *Δbc2794* deletion mutants, compared to the wild-type and *ΔcapP* strains. Biofilms were stained with crystal violet. Scale bars, 1 cm.

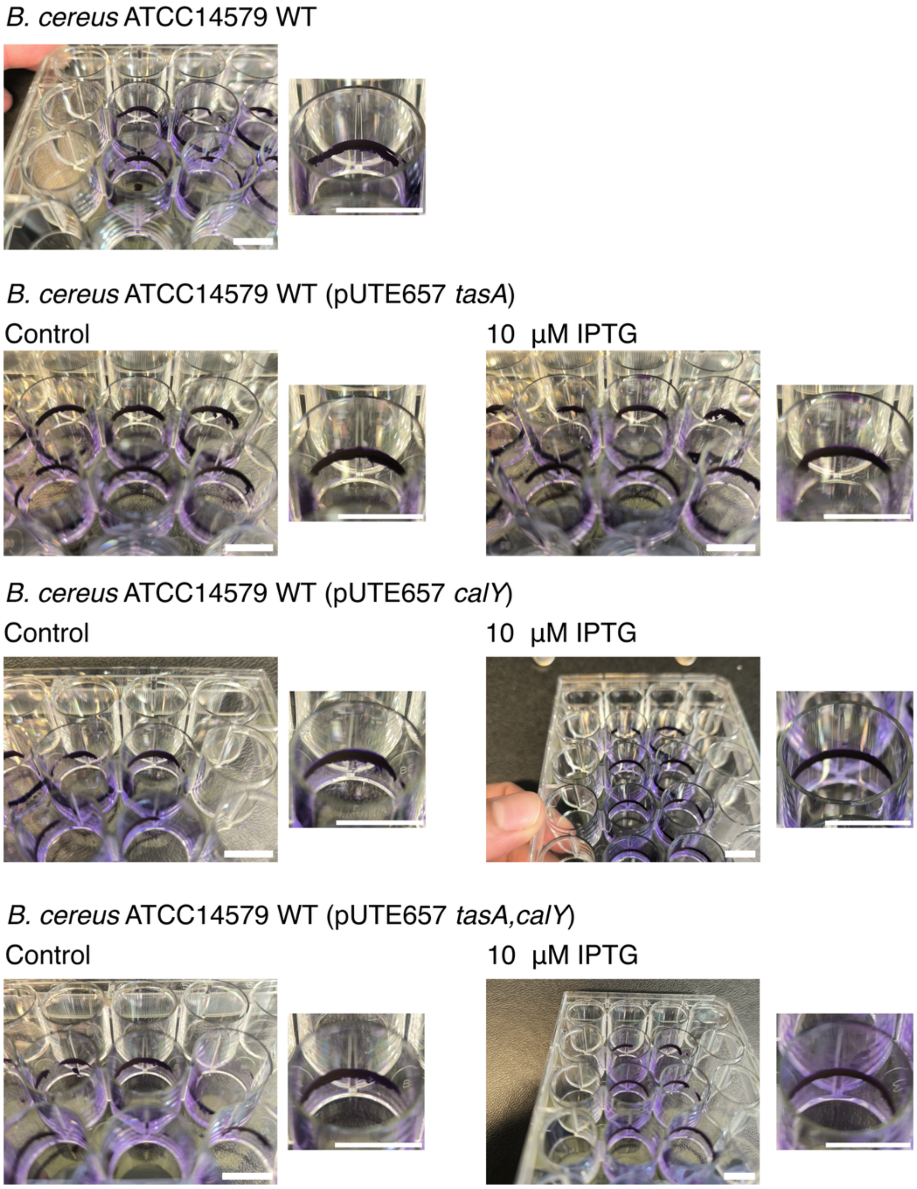

Fig. S11.

Full-plate raw data for biofilm formation of wild-type strains overexpressing *tasA*, *calY* or both genes. Raw full-plate images and cropped views used for the biofilm formation assay shown in fig. S3B. Biofilm phenotypes were assessed for the wild-type strains overexpressing *tasA*, *calY* or both genes using the pUTE657 plasmid and induction with 10 µM IPTG. As a negative control, strains carrying the plasmid were grown without IPTG. Biofilms were visualized by crystal violet staining. Scale bars, 1 cm.

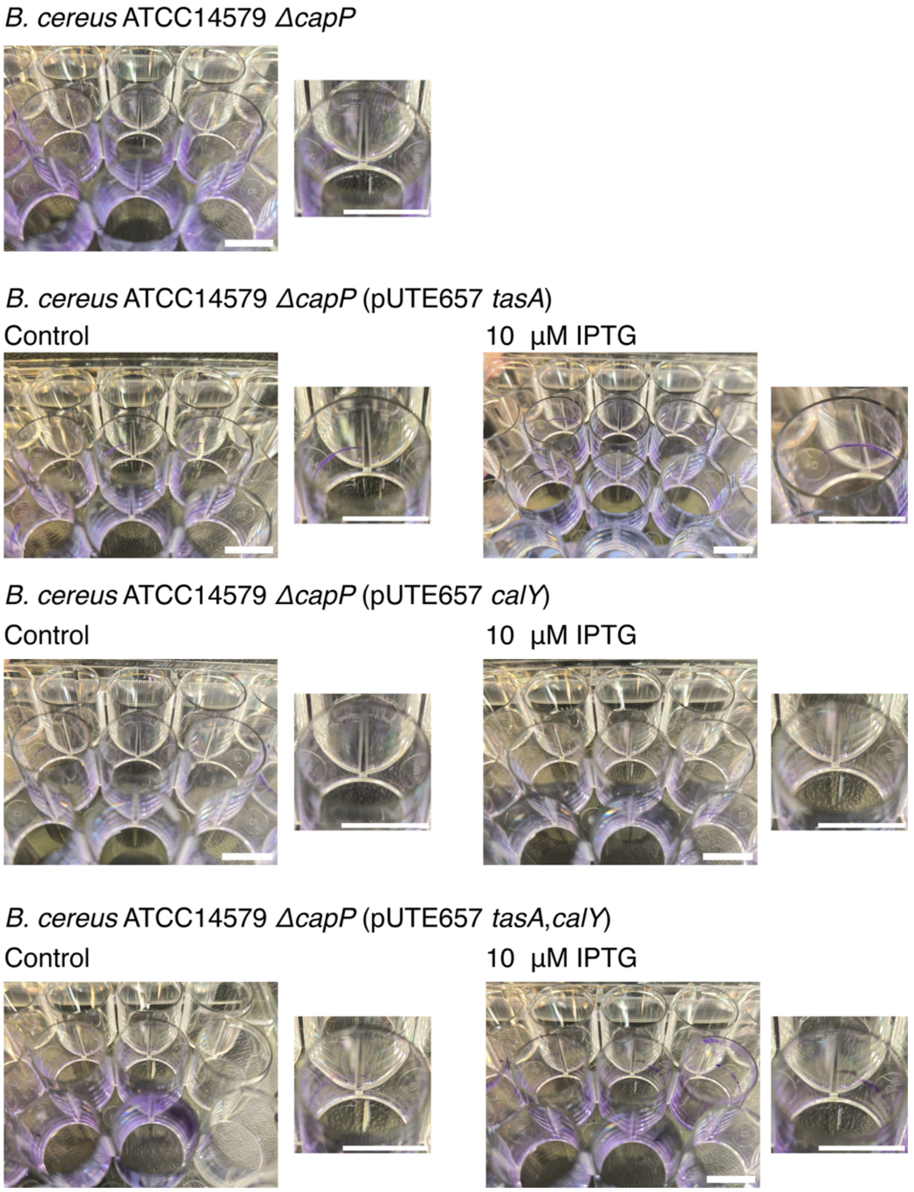

Fig. S12

Full-plate raw data for biofilm formation of *ΔcapP* strains overexpressing *tasA*, *calY* or both genes. Raw full-plate images and cropped views used for the biofilm formation assay shown in fig. S3C. Biofilm phenotypes were assessed for the *ΔcapP* strains overexpressing *tasA*, *calY* or both genes using the pUTE657 plasmid and induction with 10 µM IPTG. As a negative control, strains carrying the plasmid were grown without IPTG. Biofilms were visualized by crystal violet staining. Scale bars, 1 cm.

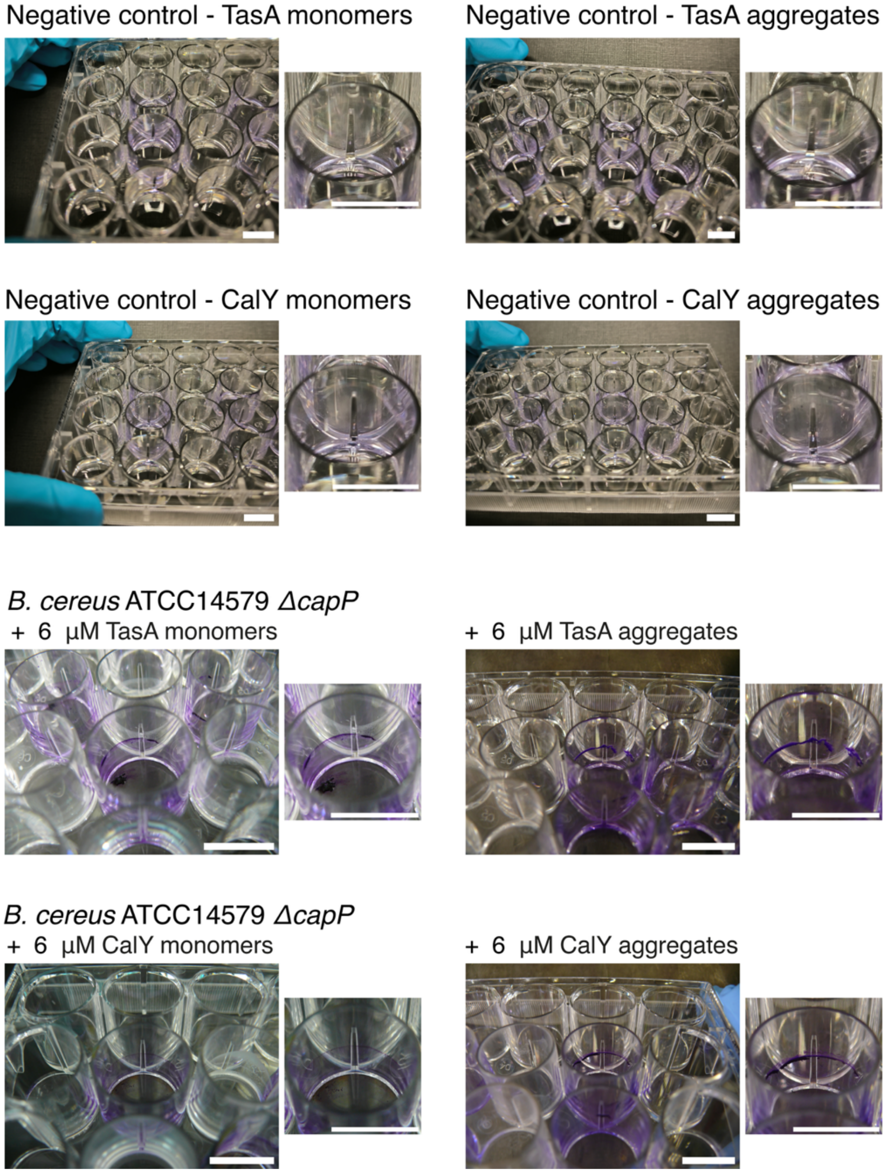

Fig. S13

Full-plate raw data for extracellular complementation of *ΔcapP* biofilms with TasA or CalY. Raw full-plate images and corresponding cropped views used for the biofilm formation assay shown in Fig. 4E. Biofilm phenotypes of the *ΔcapP* mutant were assessed after addition of 6 µM TasA or CalY (monomeric or polymerized). Negative controls without bacterial inoculation are also shown, with cropped views presented in fig. S3E. Biofilm biomass was stained with crystal violet. Scale bars, 1 cm.

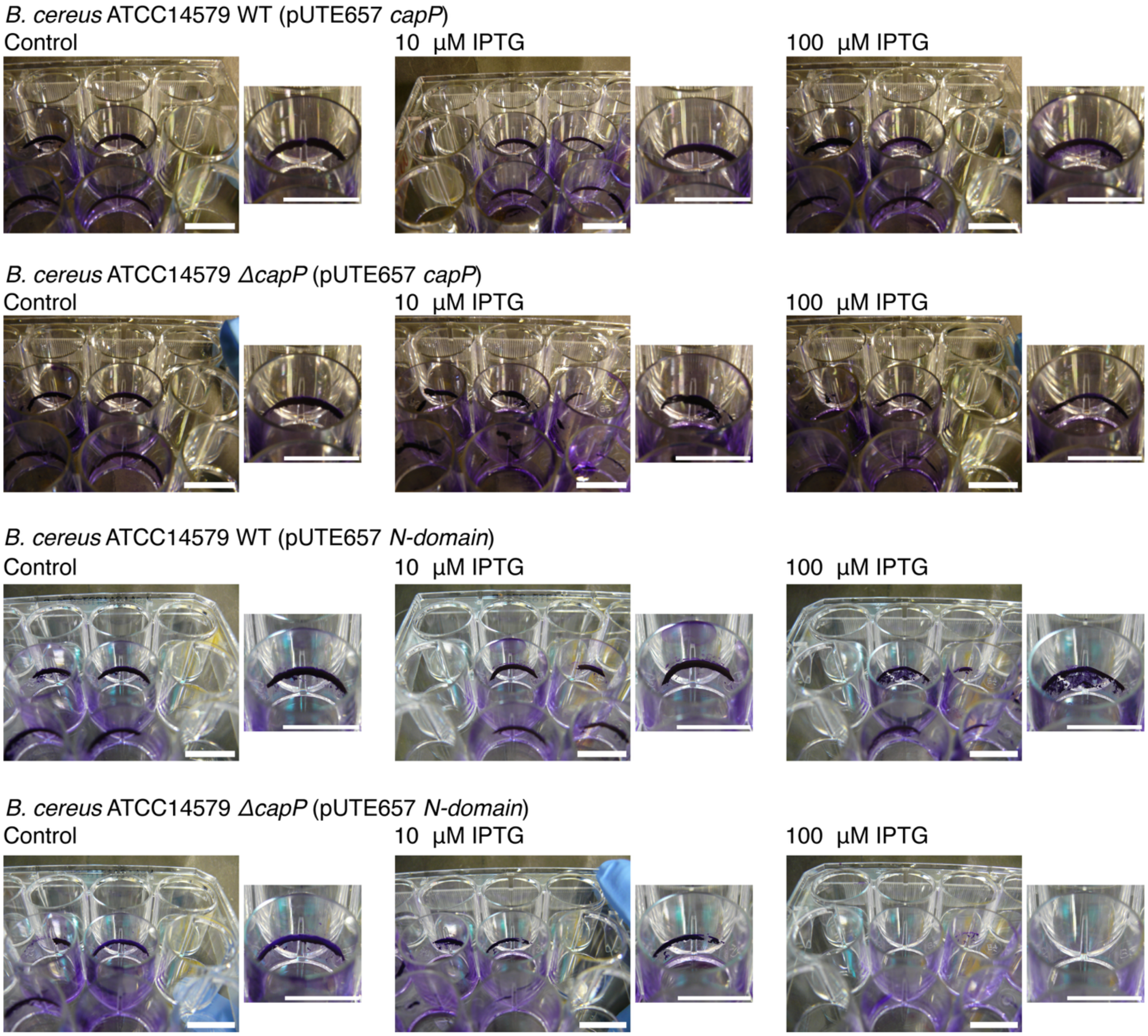

Fig. S14

Full-plate raw data for biofilm formation of wild-type and *ΔcapP* strains overexpressing full-length Cap-His or N-domain$_{39\text{-}190}$-His. Raw full-plate images and corresponding cropped views used for the biofilm formation assay shown in fig. S4A and B. Biofilm formation was assessed in wild-type and *ΔcapP* strains overexpressing either full-length CapP-His or N-domain$_{39\text{-}190}$-His, induced with 10 or 100 µM IPTG. Biofilm biomass adhered to abiotic surfaces was stained with crystal violet. Scale bars, 1 cm.

Table S1.

Biofilm formation dynamics of wild-type, *ΔtasA* and *ΔcalY* strains over time. Surface-adhered biomass was quantified by crystal violet staining. Individual values from three biological replicates, each with at least two technical replicates, are shown.

24 hours	Experiment 1			Experiment 2			Experiment 3	
WT	1.30	0.54	1.22	0.80	1.10	1.32	0.90	1.03
ΔtasA	9.62	3.86	4.22	8.80	16.22	6.94	4.42	7.73
ΔcalY	0.59	0.72	0.53	0.68	0.61	0.79	0.42	0.87
48 hours	Experiment 1			Experiment 2			Experiment 3	
WT	3.56	4.84	5.76	5.68	4.92	4.60	5.10	5.15
ΔtasA	29.72	29.09	16.80	18.00	21.81	23.20	24.05	24.52
ΔcalY	0.62	1.63	0.95	1.10	1.25	1.05	1.18	1.27
72 hours	Experiment 1			Experiment 2			Experiment 3	
WT	105.60	105.32	90.72	104.48	93.20	93.68	80.04	99.36
ΔtasA	2.11	2.54	2.45	2.61	1.97	2.41	2.61	2.58
ΔcalY	55.28	60.36	52.50	54.80	55.60	56.20	55.90	56.68

Table S2.

Individual measurements of planktonic growth (log_{10}CFU/ml) over 72 hours for wild-type, *ΔtasA* and *ΔcalY* strains. Values represent three biological replicates, each with three technical replicates.

24 hours	Experiment 1			Experiment 2			Experiment 3		
WT	5.54	5.54	5.51	5.60	5.47	5.59	5.54	5.55	5.52
ΔtasA	5.45	5.42	5.45	5.67	5.66	5.64	5.52	5.44	5.63
ΔcalY	5.08	5.05	5.12	5.32	5.41	5.34	5.30	5.24	5.22
48 hours	Experiment 1			Experiment 2			Experiment 3		
WT	6.03	6.08	6.05	6.01	6.07	6.09	6.04	6.03	6.08
ΔtasA	6.10	6.12	6.03	5.76	5.74	5.93	5.67	5.56	5.65
ΔcalY	6.17	6.20	6.22	6.23	6.18	6.16	6.24	6.24	6.26
72 hours	Experiment 1			Experiment 2			Experiment 3		
WT	5.67	5.38	5.53	5.50	5.48	5.52	5.60	5.55	5.51
ΔtasA	5.38	5.39	5.35	5.32	5.42	5.20	5.15	5.14	5.17
ΔcalY	5.70	5.66	5.71	5.40	5.38	5.44	5.55	5.57	5.53

Table S3.

Individual measurements of biofilm growth (log_{10}CFU/ml) over 72 hours for wild-type, *ΔtasA* and *ΔcalY* strains. Values represent three biological replicates, each with three technical replicates.

24 hours	Experiment 1			Experiment 2			Experiment 3		
WT	4.39	4.38	4.35	4.33	4.34	4.33	4.36	4.39	4.37
ΔtasA	5.26	5.26	5.18	5.21	5.18	5.23	5.11	5.10	5.13
ΔcalY	4.13	4.09	4.12	4.21	4.21	4.18	4.11	4.15	4.14
48 hours	Experiment 1			Experiment 2			Experiment 3		
WT	5.85	5.80	5.86	5.70	5.70	5.76	5.78	5.78	5.74
ΔtasA	5.32	5.33	5.38	5.51	5.43	5.49	5.56	5.59	5.54
ΔcalY	4.86	5.00	4.75	5.18	5.15	5.07	4.82	4.88	4.82
72 hours	Experiment 1			Experiment 2			Experiment 3		
WT	5.69	5.68	5.73	5.79	5.80	5.78	5.74	5.71	5.75
ΔtasA	5.88	5.87	5.83	5.95	5.92	5.97	5.73	5.73	5.78
ΔcalY	4.68	4.71	4.67	4.90	4.87	4.91	4.79	4.79	4.81

Table S4.

Significantly differentially expressed genes in biofilm cells of the *ΔtasA* mutant compared to the wild-type strain at 24 hours, identified by RNA-Seq analysis. Genes with a log_2(Fold Change) greater than 2 or less than -2, and a p-value < 0.05, were considered differentially expressed.

Gene ID	Annotation	Log_2FC	p-value
BC1967	no KO assigned | (GenBank) hypothetical protein	11.92	3.10E-04
BC3089	no KO assigned | (GenBank) Integral membrane protein	5.92	4.20E-06
BC0251	no KO assigned | (GenBank) hypothetical protein	5.67	2.00E-06
BC2012	no KO assigned | (GenBank) hypothetical protein	5.25	1.20E-06
BC1692	no KO assigned | (GenBank) hypothetical protein	5.22	1.90E-05
BC0249	no KO assigned | (GenBank) hypothetical protein	5.21	1.60E-04
BC0250	no KO assigned | (GenBank) hypothetical protein	4.96	3.90E-06
BC3699	no KO assigned | (GenBank) Antigen	4.93	2.10E-04
BC0247	no KO assigned | (GenBank) hypothetical protein	4.82	2.40E-04
BC1672	K07050 misacylated tRNA(Ala) deacylase [EC:3.1.1.-] | (GenBank) Metal-dependent hydrolase related to alanyl-tRNA synthetase	4.53	3.60E-04
BC4557	no KO assigned | (GenBank) hypothetical protein	4.46	4.00E-04
BC0081	K00950 2-amino-4-hydroxy-6-hydroxymethyldihydropteridine diphosphokinase [EC:2.7.6.3] | (GenBank) 2--amino-4-hydroxy-6-hydroxymethyldihydropteridine diphosphokinase	4.46	2.00E-04
BC2436	no KO assigned | (GenBank) SpoIISA like protein	4.4	1.30E-04
BC4995	K07035 uncharacterized protein | (GenBank) regulatory protein (pfoS/R)	4.37	4.50E-05
BC5033	no KO assigned | (GenBank) BC_5033	4.24	1.30E-06
BC5025	no KO assigned | (GenBank) Alkaline phosphatase like protein	4.2	5.90E-05
BC4893	K01980 23S ribosomal RNA | (GenBank) Large Subunit Ribosomal RNA	4.18	3.80E-04
BC0248	no KO assigned | (GenBank) hypothetical protein	4.14	4.20E-04
BC3088	no KO assigned | (GenBank) Metal-dependent hydrolase	4.1	1.20E-04
BC3720	K03436 DeoR family transcriptional regulator, fructose operon transcriptional repressor | (GenBank) Fructose repressor	4.07	7.80E-05
BC2186	no KO assigned | (GenBank) hypothetical protein	3.99	4.20E-04
BC0194	K16937 thiosulfate dehydrogenase (quinone) large subunit [EC:1.8.5.2] | (GenBank) hypothetical Membrane protein	3.97	5.70E-05
BC0522	K01980 23S ribosomal RNA | (GenBank) Large Subunit Ribosomal RNA	3.95	5.80E-04
BC2003	no KO assigned | (GenBank) hypothetical protein	3.94	4.90E-06

BC0595	K21903 ArsR family transcriptional regulator, lead/cadmium/zinc/bismuth-responsive transcriptional regulator	3.91	5.40E-04
BC0532	K14235 tRNA Trp | (GenBank) tRNA-Trp	3.88	8.90E-06
BC4642	K00858 NAD+ kinase [EC:2.7.1.23] | (GenBank) ATP-NAD kinase	3.81	1.40E-04
BC0410	K21828 CRP/FNR family transcriptional regulator, arginine deiminase pathway regulator | (GenBank) Transcription regulator, Crp family	3.68	3.70E-05
BC0997	no KO assigned | (GenBank) hypothetical protein	3.66	2.80E-04
BC1299	no KO assigned | (GenBank) Surface antigen	3.65	5.90E-04
BC4793	K00426 cytochrome bd ubiquinol oxidase subunit II [EC:7.1.1.7] | (GenBank) Cytochrome d ubiquinol oxidase	3.53	5.40E-04
BC5482	K03496 chromosome partitioning protein | (GenBank) Sporulation initiation inhibitor protein soj	3.51	1.40E-05
BC0596	K01534 Zn2+/Cd2+-exporting ATPase [EC:7.2.2.12 7.2.2.21] | (GenBank) Zinc-transporting ATPase	3.5	2.10E-04
BC4870	K00016 L-lactate dehydrogenase [EC:1.1.1.27] | (GenBank) L-lactate dehydrogenase	3.49	5.10E-04
BC0010	K01980 23S ribosomal RNA | (GenBank) Large Subunit Ribosomal RNA	3.47	5.40E-04
BC0448	K01537 P-type Ca2+ transporter type 2C [EC:7.2.2.10] | (GenBank) Calcium-transporting ATPase	3.38	5.60E-04
BC0301	K01980 23S ribosomal RNA | (GenBank) Large Subunit Ribosomal RNA	3.26	1.10E-04
BC4100	K01081 5'-nucleotidase [EC:3.1.3.5] | (GenBank) 5'-nucleotidase	3.25	3.20E-04
BC5186	K09812 cell division transport system ATP-binding protein | (GenBank) Cell division ATP-binding protein	3.15	1.20E-04
BC0072	K03798 cell division protease FtsH [EC:3.4.24.-] | (GenBank) Cell division protein ftsH	-3.32	5.00E-04
BC3203	no KO assigned | (GenBank) hypothetical Exported Protein	-3.59	9.30E-05
BC3026	K18220 ribosomal protection tetracycline resistance protein | (GenBank) Tetracycline resistance protein	-3.66	2.20E-04
BC5490	K02914 large subunit ribosomal protein L34 | (GenBank) LSU ribosomal protein L34P	-3.72	2.20E-05
BC5181	no KO assigned | (GenBank) hypothetical protein	-4.22	5.00E-04
BC4652	no KO assigned | (GenBank) Transcriptional regulator IcaR	-4.34	8.80E-06
BC1403	K01704 3-isopropylmalate/(R)-2-methylmalate dehydratase small subunit [EC:4.2.1.33 4.2.1.35] | (GenBank) 3-isopropylmalate dehydratase small subunit	-5.69	4.60E-04
BC2477	no KO assigned | (GenBank) hypothetical protein	-12.28	4.10E-07
BC1858	no KO assigned | (GenBank) Phage protein	-12.63	1.00E-05
BC1873	no KO assigned | (GenBank) Phage protein	-12.69	1.40E-04
BC3330	no KO assigned | (GenBank) hypothetical protein	-12.73	1.60E-04

BC0485	no KO assigned | (GenBank) hypothetical protein	-12.77	8.40E-05
BC5217	K02760 cellobiose PTS system EIIB component [EC:2.7.1.196 2.7.1.205] | (GenBank) PTS system, lichenan oligosaccharide-specific IIB component	-12.92	5.00E-04
BC3002	no KO assigned | (GenBank) hypothetical protein	-13.08	4.50E-05
BC2599	no KO assigned | (GenBank) holin	-13.45	3.50E-04
BC1778	no KO assigned | (GenBank) BC_1778	-13.46	1.50E-04
BC5039	no KO assigned | (GenBank) hypothetical protein	-13.55	6.40E-05
BC1874	no KO assigned | (GenBank) Phage protein	-13.67	6.20E-07
BC2139	no KO assigned | (GenBank) hypothetical protein	-13.77	2.30E-05
BC1418	no KO assigned | (GenBank) hypothetical protein	-13.79	2.50E-04
BC1895	no KO assigned | (GenBank) Phage protein	-14.11	1.00E-05
BC3140	no KO assigned | (GenBank) Carbonic anhydrase	-14.66	9.60E-05
BC0034	no KO assigned | (GenBank) CsfB protein	-14.68	1.00E-05
BC2754	no KO assigned | (GenBank) hypothetical protein	-14.88	2.50E-08
BC1896	no KO assigned | (GenBank) Phage protein	-14.99	6.40E-05
BC2138	no KO assigned | (GenBank) hypothetical protein	-15.54	4.70E-14
BC3610	no KO assigned | (GenBank) hypothetical protein	-17.09	2.50E-07

Table S5.

Differentially expressed proteins in *ΔtasA* biofilm cells compared to the wild-type strain at 24 hours, identified by iTRAQ analysis. Proteins with a p-value < 0.05 were considered differentially expressed.

Gene ID	Annotation	Log_2FC
BC1659	K02406 flagellin | (GenBank) Flagellin	2.579
BC1657	K02406 flagellin | (GenBank) Flagellin	2.399
BC1658	K02406 flagellin | (GenBank) Flagellin	2.231
BC3482	K01176 alpha-amylase [EC:3.2.1.1] | (GenBank) Glucan 1,4-alpha-maltohexaosidase precursor	2.139
BC0407	K00611 ornithine carbamoyltransferase [EC:2.1.3.3] | (GenBank) Ornithine carbamoyltransferase	1.831
BC5118	K01990 ABC-2 type transport system ATP-binding protein | (GenBank) ABC transporter ATP-binding protein	1.706
BC4588	K07636 two-component system, OmpR family, phosphate regulon sensor histidine kinase PhoR [EC:2.7.13.	1.561
BC2006	K03406 methyl-accepting chemotaxis protein | (GenBank) Methyl-accepting chemotaxis protein	1.525
BC5120	no KO assigned | (GenBank) hypothetical Cytosolic Protein	1.487
BC5121	no KO assigned | (GenBank) hypothetical protein	1.421
BC3101	K11035 hemolysin BL binding component | (GenBank) Hemolysin BL binding component precursor	1.364
BC1008	K03407 two-component system, chemotaxis family, sensor kinase CheA [EC:2.7.13.3] | (GenBank) Two component system histidine kinase	1.345
BC4996	K00016 L-lactate dehydrogenase [EC:1.1.1.27] | (GenBank) L-lactate dehydrogenase	1.319
BC1654	K03415 two-component system, chemotaxis family, chemotaxis protein CheV | (GenBank) Chemotaxis protein cheV	1.303
BC2273	no KO assigned | (GenBank) hypothetical protein	1.255
BC1651	K02390 flagellar hook protein FlgE | (GenBank) Flagellar hook protein flgE	1.217
BC1185	K02035 peptide/nickel transport system substrate-binding protein | (GenBank) Oligopeptide-binding protein oppA	1.198
BC5330	K00857 thymidine kinase [EC:2.7.1.21] | (GenBank) Thymidine kinase	1.192
BC5119	no KO assigned | (GenBank) hypothetical protein	1.169
BC0422	K03406 methyl-accepting chemotaxis protein | (GenBank) Methyl-accepting chemotaxis protein	1.139
BC4792	K00425 cytochrome bd ubiquinol oxidase subunit I [EC:7.1.1.7] | (GenBank) Cytochrome d ubiquinol oxidase	1.115
BC5267	no KO assigned | (GenBank) Glycosyltransferase	1.111
BC0406	K01478 arginine deiminase [EC:3.5.3.6] | (GenBank) Arginine deiminase	1.11
BC0404	K03406 methyl-accepting chemotaxis protein | (GenBank) Methyl-accepting chemotaxis protein	1.104

BC1639	K02422 flagellar secretion chaperone FliS | (GenBank) Flagellar protein fliS	0.988
BC5117	no KO assigned | (GenBank) ABC transporter permease protein	0.986
BC0366	no KO assigned | (GenBank) hypothetical protein	0.985
BC0615	K03305 proton-dependent oligopeptide transporter, POT family | (GenBank) Di-/tripeptide transporter	0.978
BC4995	K07035 uncharacterized protein | (GenBank) regulatory protein (pfoS/R)	0.968
BC3722	K08976 putative membrane protein | (GenBank) hypothetical Membrane Spanning Protein	0.923
BC1020	no KO assigned | (GenBank) hypothetical Membrane Associated Protein	0.891
BC2660	K13955 zinc-binding alcohol dehydrogenase/oxidoreductase | (GenBank) Alcohol dehydrogenase	0.885
BC2658	K08884 serine/threonine protein kinase, bacterial [EC:2.7.11.1] | (GenBank) Serine/threonine protein	0.878
BC2918	no KO assigned | (GenBank) Phosphopantothenoylcysteine decarboxylase	0.877
BC5124	no KO assigned | (GenBank) hypothetical protein	0.876
BC4513	K02556 chemotaxis protein MotA | (GenBank) Chemotaxis motA protein	0.873
BC1636	K02396 flagellar hook-associated protein 1 | (GenBank) Flagellar hook-associated protein 1	0.846
BC1179	K15580 oligopeptide transport system substrate-binding protein | (GenBank) Oligopeptide-binding protein oppA	0.827
BC5431	K25619 lipoteichoic acid glycosylation protein | (GenBank) Multidrug resistance protein B	0.804
BC0491	K00656 formate C-acetyltransferase [EC:2.3.1.54] | (GenBank) Formate acetyltransferase	0.797
BC1662	K02416 flagellar motor switch protein FliM | (GenBank) Flagellar motor switch protein fliM	0.76
BC1278	K13280 signal peptidase I [EC:3.4.21.89] | (GenBank) Signal peptidase I	0.757
BC3897	no KO assigned | (GenBank) RNA binding protein	0.739
BC5285	no KO assigned | (GenBank) Bacitracin transport ATP-binding protein bcrA	0.723
BC0081	K00950 2-amino-4-hydroxy-6-hydroxymethyldihydropteridine diphosphokinase [EC:2.7.6.3] | (GenBank) 2-2-amino-4-hydroxy-6-hydroxymethyldihydropteridine diphosphokinase	0.713
BC3102	K11035 hemolysin BL binding component | (GenBank) Hemolysin BL binding component precursor	0.709
BC5284	K01992 ABC-2 type transport system permease protein | (GenBank) ABC transporter permease protein	0.705
BC1198	K07816 GTP pyrophosphokinase [EC:2.7.6.5] | (GenBank) GTP pyrophosphokinase	0.701
BC4509	K01992 ABC-2 type transport system permease protein | (GenBank) Sodium export permease protein	0.701
BC2960	K10555 AI-2 transport system substrate-binding protein | (GenBank) Sugar-binding protein	-0.703

BC4467	K06417 stage VI sporulation protein D | (GenBank) Stage VI sporulation protein D	-0.704
BC0334	K01945 phosphoribosylamine---glycine ligase [EC:6.3.4.13] | (GenBank) Phosphoribosylamine--glycine l	-0.706
BC0898	no KO assigned | (GenBank) 3-hydroxybutyryl-CoA dehydratase	-0.708
BC4288	K13300 cytochrome c550 | (GenBank) Membrane-attached cytochrome c550	-0.708
BC0396	no KO assigned | (GenBank) ATPase	-0.712
BC3533	no KO assigned | (GenBank) Vancomycin B-type resistance protein vanW	-0.714
BC3976	no KO assigned | (GenBank) putative transcriptional regulator	-0.716
BC3322	no KO assigned | (GenBank) hypothetical protein	-0.718
BC2285	K01647 citrate synthase [EC:2.3.3.1] | (GenBank) 2-methylcitrate synthase	-0.721
BC4008	no KO assigned | (GenBank) hypothetical protein	-0.722
BC1466	no KO assigned | (GenBank) hypothetical protein	-0.722
BC0191	no KO assigned | (GenBank) hypothetical Membrane Spanning Protein	-0.727
BC3635	no KO assigned | (GenBank) hypothetical protein	-0.727
BC2484	K01961 acetyl-CoA carboxylase, biotin carboxylase subunit [EC:6.4.1.2 6.3.4.14] | (GenBank) Propiony	-0.729
BC5299	K00332 NADH-quinone oxidoreductase subunit C [EC:7.1.1.2] | (GenBank) NADH-quinone oxidoreductase ch	-0.731
BC1524	K03888 menaquinol-cytochrome c reductase cytochrome b/c subunit | (GenBank) Menaquinol-cytochrome c reductase cytochrome c subunit	-0.739
BC2235	no KO assigned | (GenBank) hypothetical protein	-0.744
BC4526	K01897 long-chain acyl-CoA synthetase [EC:6.2.1.3] | (GenBank) Long-chain-fatty-acid--CoA ligase	-0.751
BC3630	no KO assigned | (GenBank) Medium-chain-fatty-acid--CoA ligase	-0.754
BC1460	no KO assigned | (GenBank) hypothetical protein	-0.754
BC2891	no KO assigned | (GenBank) SAM-dependent methyltransferase	-0.757
BC0816	K24950 membrane fusion protein, antimicrobial resistance system | (GenBank) periplasmic component of efflux system	-0.758
BC1537	K03524 BirA family transcriptional regulator, biotin operon repressor / biotin---[acetyl-CoA-carboxylase] ligase [EC:6.3.4.15] | (GenBank) Biotin operon repressor / Biotin--[acetyl-CoA-carboxylase] synthetase	-0.761
BC1794	K15580 oligopeptide transport system substrate-binding protein | (GenBank) Oligopeptide-binding protein oppA	-0.761
BC3728	K03530 DNA-binding protein HU-beta | (GenBank) DNA-binding protein HU	-0.763
BC0328	K23265 phosphoribosylformylglycinamidine synthase subunit PurQ / glutaminase [EC:6.3.5.3 3.5.1.2] | (GenBank) Phosphoribosylformylglycinamidine synthase	-0.77
BC3353	no KO assigned | (GenBank) hypothetical protein	-0.773
BC2114	K00432 glutathione peroxidase [EC:1.11.1.9] | (GenBank) Glutathione peroxidase	-0.774

BC4867	K00700 1,4-alpha-glucan branching enzyme [EC:2.4.1.18] | (GenBank) 1,4-alpha-glucan branching enzyme	-0.779
BC1131	K03704 cold shock protein | (GenBank) Cold shock protein	-0.781
BC4659	K01895 acetyl-CoA synthetase [EC:6.2.1.1] | (GenBank) Acetyl-coenzyme A synthetase	-0.783
BC1345	no KO assigned | (GenBank) hypothetical protein	-0.783
BC3300	no KO assigned | (GenBank) hypothetical protein	-0.789
BC1610	K03851 taurine-pyruvate aminotransferase [EC:2.6.1.77] | (GenBank) Adenosylmethionine-8-amino-7-oxononanoate aminotransferase	-0.793
BC3939	K01455 formamidase [EC:3.5.1.49] | (GenBank) Amidase	-0.793
BC4789	K07173 S-ribosylhomocysteine lyase [EC:4.4.1.21] | (GenBank) Autoinducer-2 production protein luxS / Ribosylhomocysteinase	-0.795
BC3272	no KO assigned | (GenBank) hypothetical Cytosolic Protein	-0.802
BC0403	K02028 polar amino acid transport system ATP-binding protein [EC:7.4.2.1] | (GenBank) Glutamine transport ATP-binding protein glnQ	-0.804
BC2848	K15580 oligopeptide transport system substrate-binding protein | (GenBank) Oligopeptide-binding protein oppA	-0.805
BC1443	no KO assigned | (GenBank) SAM-dependent methyltransferase	-0.805
BC1927	K01999 branched-chain amino acid transport system substrate-binding protein | (GenBank) Leucine-, isoleucine-, valine-, threonine-, and alanine-binding protein	-0.807
BC4100	K01081 5'-nucleotidase [EC:3.1.3.5] | (GenBank) 5'-nucleotidase	-0.809
BC3395	no KO assigned | (GenBank) hypothetical protein	-0.81
BC4256	no KO assigned | (GenBank) Transcriptional regulator, ArsR family	-0.811
BC1940	K16013 ATP-binding cassette, subfamily C, bacterial CydD | (GenBank) Transport ATP-binding protein c	-0.812
BC4334	K06371 developmental checkpoint coupling sporulation initiation to replication initiation | (GenBank) hypothetical protein	-0.821
BC1399	K00053 ketol-acid reductoisomerase [EC:1.1.1.86] | (GenBank) Ketol-acid reductoisomerase	-0.822
BC3631	no KO assigned | (GenBank) Medium-chain-fatty-acid--CoA ligase	-0.822
BC1712	K01679 fumarate hydratase, class II [EC:4.2.1.2] | (GenBank) Fumarate hydratase	-0.827
BC4334	K06371 developmental checkpoint coupling sporulation initiation to replication initiation | (GenBank) hypothetical protein	-0.827
BC0519	no KO assigned | (GenBank) Ribosomal-protein-alanine acetyltransferase	-0.828
BC2005	K06949 ribosome biogenesis GTPase / thiamine phosphate phosphatase [EC:3.6.1.- 3.1.3.100] | (GenBank) GTPase	-0.83
BC0625	K04748 nitric oxide reductase NorQ protein | (GenBank) NorQ protein	-0.841
BC3133	K06889 uncharacterized protein | (GenBank) putative hydrolase	-0.852
BC4089	K04763 integrase/recombinase XerD | (GenBank) DNA integration/recombination/invertion protein	-0.852
BC2313	K03530 DNA-binding protein HU-beta | (GenBank) DNA-binding protein HU	-0.864

BC4251	K24042 methionine synthase / methylenetetrahydrofolate reductase (NADH) [EC:2.1.1.13 1.5.1.54] | (GenBank) 5-methyltetrahydrofolate--homocysteine methyltransferase	-0.873
BC2605	no KO assigned | (GenBank) hypothetical protein	-0.881
BC2849	K20742 gamma-D-glutamyl-L-lysine dipeptidyl-peptidase [EC:3.4.14.13] | (GenBank) Cell wall-associated hydrolase	-0.888
BC2194	K01118 FMN-dependent NADH-azoreductase [EC:1.7.1.17] | (GenBank) FMN-dependent NADH-azoreductase	-0.89
BC1708	no KO assigned | (GenBank) hypothetical protein	-0.894
BC0329	K23269 phosphoribosylformylglycinamidine synthase subunit PurL [EC:6.3.5.3] | (GenBank) Phosphoribosylformylglycinamidine synthase	-0.899
BC1410	K02500 imidazole glycerol-phosphate synthase subunit HisF [EC:4.3.2.10] | (GenBank) HisF protein	-0.901
BC5106	K25282 iron-siderophore transport system substrate-binding protein | (GenBank) Ferric anguibactin-binding protein	-0.902
BC3562	no KO assigned | (GenBank) hypothetical protein	-0.906
BC2942	K13853 3-deoxy-7-phosphoheptulonate synthase / chorismate mutase [EC:2.5.1.54 5.4.99.5] | (GenBank) Chorismate mutase	-0.911
BC5359	K19701 aminopeptidase YwaD [EC:3.4.11.6 3.4.11.10] | (GenBank) Aminopeptidase Y	-0.914
BC1412	K01523 phosphoribosyl-ATP pyrophosphohydrolase [EC:3.6.1.31] | (GenBank) Phosphoribosyl-ATP pyrophosphatase	-0.917
BC0402	K02030 polar amino acid transport system substrate-binding protein | (GenBank) Cystine-binding protein	-0.918
BC0640	K10039 aspartate/glutamate/glutamine transport system substrate-binding protein | (GenBank) Glutamine-binding protein	-0.919
BC3776	K06416 stage V sporulation protein S | (GenBank) Stage V sporulation protein S	-0.921
BC4645	K01895 acetyl-CoA synthetase [EC:6.2.1.1] | (GenBank) Acetyl-coenzyme A synthetase	-0.922
BC2051	no KO assigned | (GenBank) Ribosomal-protein-alanine acetyltransferase	-0.934
BC0813	no KO assigned | (GenBank) enterotoxin / cell-wall binding protein	-0.938
BC1209	no KO assigned | (GenBank) O-antigen biosynthesis protein rfbC	-0.938
BC2967	no KO assigned | (GenBank) Fructose-bisphosphate aldolase	-0.939
BC5101	K11031 thiol-activated cytolysin | (GenBank) Perfringolysin O precursor	-0.94
BC1912	no KO assigned | (GenBank) Phage protein	-0.943
BC3607	K06306 spore germination protein | (GenBank) spore peptidoglycan hydrolase (N-acetylglucosaminidase)	-0.95
BC1025	K15975 glyoxalase family protein | (GenBank) Glyoxalase family protein	-0.957
BC0456	K03100 signal peptidase I [EC:3.4.21.89] | (GenBank) Signal peptidase I	-0.96
BC0821	K06334 spore coat protein JC | (GenBank) CotJC protein	-0.969
BC0059	K04769 AbrB family transcriptional regulator, stage V sporulation protein T | (GenBank) Stage V sporulation protein T	-0.969
BC2102	no KO assigned | (GenBank) hypothetical protein	-0.969

BC5297	K00337 NADH-quinone oxidoreductase subunit H [EC:7.1.1.2] | (GenBank) NADH-quinone oxidoreductase chain H	-0.973
BC0062	no KO assigned | (GenBank) Heat shock protein 15	-0.989
BC0385	K21567 ferredoxin/flavodoxin---NADP+ reductase [EC:1.18.1.2 1.19.1.1] | (GenBank) Thioredoxin reductase	-1.004
BC4501	K01994 LuxR family transcriptional regulator, transcriptional regulator of spore coat protein | (GenBank) Germination protein gerE	-1.006
BC5145	no KO assigned | (GenBank) hypothetical protein	-1.016
BC0326	K01923 phosphoribosylaminoimidazole-succinocarboxamide synthase [EC:6.3.2.6] | (GenBank) Phosphoribosylamidoimidazole-succinocarboxamide synthase	-1.033
BC3068	K06351 inhibitor of KinA | (GenBank) Kinase autophosphorylation inhibitor kipI	-1.038
BC3930	K08316 16S rRNA (guanine966-N2)-methyltransferase [EC:2.1.1.171] | (GenBank) Methyltransferase	-1.04
BC0787	K06415 stage V sporulation protein R | (GenBank) Stage V sporulation protein R	-1.044
BC5406	K01740 O-acetylhomoserine (thiol)-lyase [EC:2.5.1.49] | (GenBank) O-acetylhomoserine sulfhydrylase	-1.045
BC0333	K00602 phosphoribosylaminoimidazolecarboxamide formyltransferase / IMP cyclohydrolase [EC:2.1.2.3 3.5.4.10] | (GenBank) IMP cyclohydrolase	-1.049
BC2969	K07145 heme oxygenase (staphylobilin-producing) [EC:1.14.99.48] | (GenBank) hypothetical protein	-1.075
BC0331	K01933 phosphoribosylformylglycinamidine cyclo-ligase [EC:6.3.3.1] | (GenBank) Phosphoribosylformylglycinamidine cyclo-ligase	-1.078
BC0160	K16786 energy-coupling factor transport system ATP-binding protein [EC:7.-.-.-] | (GenBank) Cobalt transport ATP-binding protein cbiO	-1.085
BC0786	no KO assigned | (GenBank) Nitroreductase family	-1.104
BC2861	K01281 X-Pro dipeptidyl-peptidase [EC:3.4.14.11] | (GenBank) Xaa-Pro dipeptidyl-peptidase	-1.126
BC0327	K23264 phosphoribosylformylglycinamidine synthase subunit PurS [EC:6.3.5.3] | (GenBank) Phosphorybosylformylglycinamidine synthetase, PurS component	-1.127
BC1781	K01754 threonine dehydratase [EC:4.3.1.19] | (GenBank) Threonine dehydratase	-1.13
BC2392	no KO assigned | (GenBank) FenI	-1.14
BC1984	no KO assigned | (GenBank) Small acid-soluble spore protein	-1.14
BC5209	K01222 6-phospho-beta-glucosidase [EC:3.2.1.86] | (GenBank) 6-phospho-beta-glucosidase	-1.148
BC3585	K15580 oligopeptide transport system substrate-binding protein | (GenBank) Oligopeptide-binding protein oppA	-1.165
BC1269	no KO assigned | (GenBank) hypothetical protein	-1.166
BC3584	K15580 oligopeptide transport system substrate-binding protein | (GenBank) Oligopeptide-binding protein oppA	-1.175
BC2252	no KO assigned | (GenBank) hypothetical Cytosolic Protein	-1.179

BC1127	K01638 malate synthase [EC:2.3.3.9] | (GenBank) Malate synthase	-1.179
BC3800	K06411 dipicolinate synthase subunit B | (GenBank) Dipicolinate synthase, B chain	-1.189
BC0638	K03310 alanine or glycine:cation symporter, AGCS family | (GenBank) Sodium/proton-dependent alanine carrier protein	-1.215
BC4074	K06378 stage II sporulation protein AA (anti-sigma F factor antagonist) | (GenBank) Anti-sigma F factor	-1.242
BC4187	K06396 stage III sporulation protein AG | (GenBank) Stage III sporulation protein AG	-1.244
BC2745	K18640 plasmid segregation protein ParM | (GenBank) hypothetical protein	-1.253
BC1284	K09607 immune inhibitor A [EC:3.4.24.-] | (GenBank) Immune inhibitor A precursor	-1.255
BC4281	no KO assigned | (GenBank) IG hypothetical 17696	-1.293
BC1506	no KO assigned | (GenBank) hypothetical protein	-1.31
BC0451	no KO assigned | (GenBank) hypothetical protein	-1.31
BC2524	K08602 oligoendopeptidase F [EC:3.4.24.-] | (GenBank) Oligoendopeptidase F	-1.311
BC4073	K06379 stage II sporulation protein AB (anti-sigma F factor) [EC:2.7.11.1] | (GenBank) Anti-sigma F factor	-1.319
BC5391	K06305 spore germination protein Q | (GenBank) hypothetical protein	-1.349
BC2026	K15580 oligopeptide transport system substrate-binding protein | (GenBank) Oligopeptide-binding protein oppA	-1.374
BC5035	no KO assigned | (GenBank) Phenylacetic acid degradation protein paaD	-1.378
BC3636	no KO assigned | (GenBank) hypothetical Cytosolic Protein	-1.406
BC1206	K05303 O-methyltransferase [EC:2.1.1.-] | (GenBank) Macrocin O-methyltransferase	-1.421
BC2608	no KO assigned | (GenBank) hypothetical Cytosolic Protein	-1.439
BC3158	no KO assigned | (GenBank) Pyrimidine Reductase	-1.455
BC2244	no KO assigned | (GenBank) hypothetical protein	-1.462
BC3738	K02016 iron complex transport system substrate-binding protein | (GenBank) Iron(III) dicitrate-binding protein	-1.48
BC4071	K03308 neurotransmitter:Na+ symporter, NSS family | (GenBank) Sodium/proline symporter	-1.484
BC1091	no KO assigned | (GenBank) Sulfate-binding protein	-1.533
BC5390	K01449 N-acetylmuramoyl-L-alanine amidase [EC:3.5.1.28] | (GenBank) Cell wall hydrolase cwlJ	-1.538
BC4419	K06345 spore cortex protein | (GenBank) hypothetical protein	-1.655
BC3770	K06328 spore coat protein E | (GenBank) Spore coat protein E	-1.681
BC3091	K06418 small acid-soluble spore protein A (major alpha-type SASP) | (GenBank) Small acid-soluble spore protein	-1.706
BC3541	K03839 flavodoxin I | (GenBank) Flavodoxin	-1.713
BC3390	K07078 uncharacterized protein | (GenBank) Nitroreductase family protein	-1.841

BC5282	K06283 putative DeoR family transcriptional regulator, stage III sporulation protein D | (GenBank) S	-1.849
BC0548	K07180 serine protein kinase | (GenBank) Serine protein kinase	-1.878
BC0063	no KO assigned | (GenBank) hypothetical protein	-1.977
BC1128	K01637 isocitrate lyase [EC:4.1.3.1] | (GenBank) Isocitrate lyase	-1.981
BC1509	K06398 stage IV sporulation protein A | (GenBank) Stage IV sporulation protein A	-2.039
BC3784	no KO assigned | (GenBank) IG hypothetical 16623	-2.265
BC3922	no KO assigned | (GenBank) Prespore specific transcriptional activator rsfA	-2.455
BC3326	no KO assigned | (GenBank) hypothetical protein	-2.457
BC0875	K06419 small acid-soluble spore protein B (major beta-type SASP) | (GenBank) Small acid-soluble spore protein	-2.954
BC4646	no KO assigned | (GenBank) Small acid-soluble spore protein	-3.19
BC4420	K06370 morphogenetic protein associated with SpoVID | (GenBank) SpoVID-dependent spore coat assembly	-3.392
BC1279	K06336 spore coat-associated protein N | (GenBank) Spore coat-associated protein N	-3.49
BC0506	K06422 small acid-soluble spore protein E (minor gamma-type SASP) | (GenBank) Small, acid-soluble spore protein gamma-type	-3.724
BC2943	no KO assigned | (GenBank) hypothetical protein	-4.505

Table S6.

Colony-forming unit (CFU) counts during biofilm development for *B. cereus* ATCC14579 wild-type, *ΔtasA*, *Δflag* and *ΔtasA,flag* strains. Data are from three biological replicates, each with three technical replicates.

24 hours	Experiment 1			Experiment 2			Experiment 3		
WT	4.38	4.33	4.37	4.32	4.39	4.34	4.39	4.32	4.36
ΔtasA	5.26	5.10	5.12	5.13	5.29	5.07	5.10	5.18	5.23
Δflag	5.67	4.76	5.00	6.10	6.05	5.85	4.45	4.40	4.30
ΔtasA,flag	3.00	3.30	3.48	3.46	3.33	3.40	3.06	3.12	3.19
48 hours	Experiment 1			Experiment 2			Experiment 3		
WT	5.85	5.70	5.77	5.92	5.87	5.72	5.82	5.67	5.62
ΔtasA	5.32	5.56	5.51	5.58	5.50	5.39	5.53	5.34	5.42
Δflag	5.88	5.95	5.60	5.91	5.86	5.76	5.71	5.66	5.96
ΔtasA,flag	5.54	5.20	5.47	4.60	4.76	5.37	4.99	5.22	4.84
72 hours	Experiment 1			Experiment 2			Experiment 3		
WT	5.69	5.79	5.73	5.66	5.69	5.72	5.74	5.77	5.81
ΔtasA	5.88	5.95	5.73	5.94	5.90	5.86	5.84	5.80	5.76
Δflag	6.40	6.40	6.28	6.30	6.33	6.35	6.42	6.37	6.39
ΔtasA,flag	6.48	6.59	6.54	6.53	6.57	6.55	6.53	6.51	6.49

Table S7.

Differentially expressed genes in *ΔcalY* biofilm cells compared to the wild-type strain at 24 hours, identified by RNA-Seq. Genes with a log_2(Fold Change) value >2 or <-2, and a p-value < 0.05, were considered differentially expressed.

Gene ID	Annotation	Log_2FC	p-value
BC2358	K03704 cold shock protein | (GenBank) Cold shock protein	3.83	1.20E-05
BC0665	K00616 transaldolase [EC:2.2.1.2] | (GenBank) Transaldolase	-3.29	5.60E-05
BC2229	K03206 azobenzene reductase [EC:1.7.1.6] | (GenBank) Azoreductase	-3.37	4.60E-05
BC0465	no KO assigned | (GenBank) hypothetical protein	-3.76	5.00E-05
BC0533	K14226 tRNA His | (GenBank) tRNA-His	-3.89	6.00E-05
BC0590	no KO assigned | (GenBank) hypothetical protein	-4.03	2.00E-06
BC1281	no KO assigned | (GenBank) Cell envelope-bound metalloprotease (camelysin)	-6.36	6.50E-07

Table S8.

Significant transcriptional changes in biofilm cells of *ΔcalY* compared to the wild-type strain at 72 hours, identified by RNA-Seq. Genes with a $\log_2$(Fold Change) value >2 or <-2, and a p-value < 0.05, were considered differentially expressed.

Gene ID	Annotation	Log_2FC	p-value
BC1322	no KO assigned | (GenBank) hypothetical protein	18.04	7.10E-09
BC3458	no KO assigned | (GenBank) XpaF1 protein	17.55	3.50E-08
BC4711	no KO assigned | (GenBank) hypothetical protein	17.22	1.50E-05
BC0485	no KO assigned | (GenBank) hypothetical protein	16.69	4.60E-06
BC1253	K07729 putative transcriptional regulator | (GenBank) Transcriptional regulator, PBSX family	16.66	1.10E-09
BC4713	no KO assigned | (GenBank) hypothetical protein	16.31	4.90E-11
BC4191	K06392 stage III sporulation protein AC | (GenBank) Stage III sporulation protein AC	16.27	1.20E-10
BC4858	no KO assigned | (GenBank) DnaK suppressor protein	16.09	1.00E-10
BC4772	no KO assigned | (GenBank) BC_4772	16.03	4.90E-09
BC5147	K06405 stage V sporulation protein AC | (GenBank) Stage V sporulation protein AC	15.99	2.20E-06
BC1882	no KO assigned | (GenBank) Phage protein	15.93	1.90E-06
BC4500	no KO assigned | (GenBank) ThiJ/PfpI family protein	15.9	5.40E-07
BC1219	no KO assigned | (GenBank) Integral membrane protein	15.83	2.60E-05
BC0904	no KO assigned | (GenBank) hypothetical protein	15.78	4.90E-11
BC2845	no KO assigned | (GenBank) hypothetical protein	15.74	3.90E-05
BC0853	K18925 paired small multidrug resistance pump | (GenBank) Quaternary ammonium compound-resistance protein	15.41	4.60E-04
BC0822	K06333 spore coat protein JB | (GenBank) CotJB protein	15.28	1.20E-11
BC5024	K07729 putative transcriptional regulator | (GenBank) Transcriptional regulator, PBSX family	15.22	5.10E-04
BC4997	no KO assigned | (GenBank) hypothetical protein	15.17	2.20E-10
BC5068	K06199 fluoride exporter | (GenBank) CrcB family protein	15.13	3.50E-12
BC2149	no KO assigned | (GenBank) hypothetical protein	15.07	1.90E-09
BC1867	no KO assigned | (GenBank) Phage protein	15.06	1.20E-03
BC3381	no KO assigned | (GenBank) hypothetical protein	15.05	1.50E-03
BC0479	no KO assigned | (GenBank) hypothetical protein	15.03	4.00E-06
BC5287	K06381 stage II sporulation protein D | (GenBank) Stage II sporulation protein D	15.02	5.10E-08
BC1171	no KO assigned | (GenBank) Glucose epimerase	14.99	2.60E-05
BC2697	no KO assigned | (GenBank) Translation initiation inhibitor	14.95	3.40E-10
BC3179	no KO assigned | (GenBank) hypothetical protein	14.88	1.10E-03
BC3514	K00978 glucose-1-phosphate cytidylyltransferase [EC:2.7.7.33] | (GenBank) Glucose-1-phosphate cytidylyltransferase	14.8	7.80E-07

BC3396	K08987 putative membrane protein | (GenBank) hypothetical Membrane Spanning Protein	14.73	2.80E-06
BC4355	no KO assigned | (GenBank) hypothetical Membrane Spanning Protein	14.72	1.20E-04
BC3041	no KO assigned | (GenBank) hypothetical protein	14.53	6.10E-09
BC4641	no KO assigned | (GenBank) hypothetical protein	14.53	3.20E-08
BC1981	K24111 aryl carrier protein AsbD | (GenBank) Acyl carrier protein	14.46	5.60E-05
BC3715	no KO assigned | (GenBank) hypothetical protein	14.41	1.30E-06
BC0614	K06425 small acid-soluble spore protein H (minor) | (GenBank) Small, acid-soluble spore protein	14.26	1.30E-03
BC3191	no KO assigned | (GenBank) Transporter, Drug/Metabolite Exporter family	14.23	7.60E-04
BC2516	no KO assigned | (GenBank) Short chain dehydrogenase	14.19	1.40E-03
BC0891	no KO assigned | (GenBank) hypothetical protein	14.03	9.50E-04
BC5056	no KO assigned | (GenBank) Collagen adhesion protein	14.02	4.00E-10
BC3677	no KO assigned | (GenBank) N-acetylmuramoyl-L-alanine amidase	14.02	4.90E-05
BC2383	no KO assigned | (GenBank) Oxalate decarboxylase	13.8	7.70E-06
BC4190	K06393 stage III sporulation protein AD | (GenBank) Stage III sporulation protein AD	13.78	1.40E-05
BC2575	no KO assigned | (GenBank) DNA integration/recombination/invertion protein	13.75	1.40E-03
BC5027	no KO assigned | (GenBank) Protein erfK/srfK precursor	13.71	3.80E-11
BC2946	no KO assigned | (GenBank) Integral membrane protein	13.62	3.80E-07
BC4577	no KO assigned | (GenBank) hypothetical protein	13.57	9.60E-08
BC5217	K02760 cellobiose PTS system EIIB component [EC:2.7.1.196 2.7.1.205] | (GenBank) PTS system, lichenan oligosaccharide-specific IIB component	13.54	4.00E-04
BC5243	no KO assigned | (GenBank) hypothetical protein	13.51	2.30E-04
BC0477	no KO assigned | (GenBank) Transcriptional regulator	13.5	8.10E-04
BC2465	no KO assigned | (GenBank) hypothetical protein	13.45	1.30E-06
BC3987	no KO assigned | (GenBank) NRDH-redoxin	13.42	6.80E-06
BC2098	no KO assigned | (GenBank) hypothetical protein	13.31	7.40E-05
BC4651	no KO assigned | (GenBank) Transporter, Drug/Metabolite Exporter family	13.29	1.30E-03
BC2599	no KO assigned | (GenBank) holin	13.26	4.50E-04
BC5218	no KO assigned | (GenBank) Proton/sodium-glutamate symport protein	13.24	3.50E-09
BC2752	K06313 spore germination protein | (GenBank) hypothetical Membrane Spanning Protein	13.14	9.30E-05
BC1839	no KO assigned | (GenBank) hypothetical Membrane Associated Protein	13.08	3.80E-05
BC0171	no KO assigned | (GenBank) Chitooligosaccharide deacetylase	13.04	1.40E-09

BC2651	no KO assigned | (GenBank) hypothetical protein	13.03	1.70E-03
BC1234	K00766 anthranilate phosphoribosyltransferase [EC:2.4.2.18] | (GenBank) Anthranilate phosphoribosyltransferase	13.02	1.00E-03
BC0422	K03406 methyl-accepting chemotaxis protein | (GenBank) Methyl-accepting chemotaxis protein	12.91	1.60E-09
BC5208	no KO assigned | (GenBank) cellobiose phosphotransferase system celC	12.87	7.30E-05
BC5149	K06407 stage V sporulation protein AE | (GenBank) Stage V sporulation protein AE	12.87	6.00E-07
BC5150	no KO assigned | (GenBank) hypothetical protein	12.84	4.10E-04
BC4417	K06318 forespore regulator of the sigma-K checkpoint | (GenBank) BofC protein	12.69	3.50E-07
BC0210	K02032 peptide/nickel transport system ATP-binding protein | (GenBank) Oligopeptide transport ATP-binding protein oppF	12.64	8.00E-04
BC1742	K07717 two-component system, sensor histidine kinase GlnK [EC:2.7.13.3] | (GenBank) Two component system histidine kinase	12.64	1.30E-03
BC5346	K06131 cardiolipin synthase A/B [EC:2.7.8.-] | (GenBank) Cardiolipin synthase	12.62	1.30E-03
BC0167	K01448 N-acetylmuramoyl-L-alanine amidase [EC:3.5.1.28] | (GenBank) Spore-specific N-acetylmuramoyl-L-alanine amidase	12.56	1.10E-06
BC2463	no KO assigned | (GenBank) hypothetical protein	12.51	5.60E-07
BC1842	no KO assigned | (GenBank) RRF2 family protein	12.51	4.20E-04
BC2994	no KO assigned | (GenBank) hypothetical protein	12.49	3.30E-04
BC2552	no KO assigned | (GenBank) hypothetical protein	12.49	4.80E-07
BC4897	no KO assigned | (GenBank) IG hypothetical 16740	12.32	5.40E-04
BC2746	no KO assigned | (GenBank) hypothetical protein	12.26	3.90E-04
BC3417	no KO assigned | (GenBank) hypothetical protein	12.26	2.60E-05
BC3142	K05577 NAD(P)H-quinone oxidoreductase subunit 5 [EC:7.1.1.2] | (GenBank) NADH-quinone oxidoreductase	12.13	1.10E-03
BC4362	K02015 iron complex transport system permease protein | (GenBank) Ferrichrome transport system permease protein fhuB	12.03	1.40E-04
BC2302	K00216 2,3-dihydro-2,3-dihydroxybenzoate dehydrogenase [EC:1.3.1.28] | (GenBank) 2,3-dihydro-2,3-dihydroxybenzoate dehydrogenase	12.02	2.20E-06
BC0225	no KO assigned | (GenBank) Transporter, Drug/Metabolite Exporter family	11.95	1.90E-03
BC1743	K07719 two-component system, response regulator GlnL | (GenBank) Two-component response regulator	11.92	4.20E-05
BC2350	no KO assigned | (GenBank) Transporter, Drug/Metabolite Exporter family	11.88	2.30E-04
BC1162	no KO assigned | (GenBank) hypothetical protein	11.87	3.10E-04

BC2303	K02361 isochorismate synthase [EC:5.4.4.2] | (GenBank) Isochorismate synthase	11.67	3.00E-05
BC2712	no KO assigned | (GenBank) hypothetical protein	11.64	7.30E-04
BC1503	no KO assigned | (GenBank) hypothetical protein	11.42	5.30E-04
BC4192	K06391 stage III sporulation protein AB | (GenBank) Stage III sporulation protein AB	11.24	2.70E-05
BC3384	no KO assigned | (GenBank) Enhancin	11.19	1.30E-03
BC3928	no KO assigned | (GenBank) hypothetical Membrane Spanning Protein	11.15	1.20E-04
BC3140	no KO assigned | (GenBank) Carbonic anhydrase	10.89	1.30E-03
BC1835	K01118 FMN-dependent NADH-azoreductase [EC:1.7.1.17] | (GenBank) FMN-dependent NADH-azoreductase	10.64	1.80E-03
BC1032	no KO assigned | (GenBank) Transcriptional repressor	10.47	1.70E-04
BC0421	K07791 anaerobic C4-dicarboxylate transporter DcuA | (GenBank) Anaerobic C4-dicarboxylate transporter	10.43	2.60E-04
BC0319	no KO assigned | (GenBank) hypothetical protein	10.31	5.40E-04
BC3468	K25288 ferric hydroxamate/heme transport system permease protein | (GenBank) Ferrichrome transport system permease protein fhuB	10.3	1.70E-04
BC1081	no KO assigned | (GenBank) PlcR-regulated protein PRP2	10.19	4.30E-04
BC0480	no KO assigned | (GenBank) hypothetical protein	9.42	5.90E-04
BC1620	no KO assigned | (GenBank) hypothetical membrane protein	9.35	2.10E-03
BC1851	no KO assigned | (GenBank) Transcriptional regulator	9.23	4.90E-04
BC4208	no KO assigned | (GenBank) hypothetical protein	9.14	3.60E-08
BC5326	no KO assigned | (GenBank) hypothetical protein	8.59	1.70E-03
BC0758	no KO assigned | (GenBank) Transcriptional regulator, MarR family	7.9	8.60E-07
BC1376	K03839 flavodoxin I | (GenBank) Flavodoxin	7.61	5.50E-05
BC2965	K11216 autoinducer-2 kinase [EC:2.7.1.189] | (GenBank) Sugar (pentulose and hexulose) kinases	7.19	2.70E-05
BC4360	no KO assigned | (GenBank) hypothetical Cytosolic Protein	7.07	6.90E-04
BC0640	K10039 aspartate/glutamate/glutamine transport system substrate-binding protein | (GenBank) Glutamine-binding protein	6.93	3.40E-04
BC3373	no KO assigned | (GenBank) BC_3373	6.77	5.20E-04
BC2553	no KO assigned | (GenBank) hypothetical protein	6.57	5.70E-12
BC2964	K11531 lsr operon transcriptional repressor | (GenBank) Deoxyribonucleoside regulator	6.47	7.20E-05
BC0315	K01977 16S ribosomal RNA | (GenBank) Small Subunit Ribosomal RNA	6.36	6.30E-06
BC0715	K01977 16S ribosomal RNA | (GenBank) Small Subunit Ribosomal RNA	6.11	2.30E-04

BC1140	K06304 spore germination protein PF | (GenBank) Spore germination protein PF	6.1	1.80E-05
BC4894	K01977 16S ribosomal RNA | (GenBank) Small Subunit Ribosomal RNA	6.02	3.40E-06
BC2141b	no KO assigned | (GenBank) Lipase	5.93	2.00E-03
BC5390	K01449 N-acetylmuramoyl-L-alanine amidase [EC:3.5.1.28] | (GenBank) Cell wall hydrolase cwlJ	5.78	5.30E-04
BC0281	K01977 16S ribosomal RNA | (GenBank) Small Subunit Ribosomal RNA	5.64	6.40E-06
BC0670	K01114 phospholipase C [EC:3.1.4.3] | (GenBank) Phospholipase C	5.52	1.60E-04
BC1010	no KO assigned | (GenBank) hypothetical protein	5.51	9.80E-06
BC1213	K01790 dTDP-4-dehydrorhamnose 3,5-epimerase [EC:5.1.3.13] | (GenBank) dTDP-4-dehydrorhamnose 3,5-epimerase	5.48	5.60E-05
BC0320	K01977 16S ribosomal RNA | (GenBank) Small Subunit Ribosomal RNA	5.38	3.80E-06
BC0173	K01977 16S ribosomal RNA | (GenBank) Small Subunit Ribosomal RNA	5.36	1.40E-05
BC0300	K01977 16S ribosomal RNA | (GenBank) Small Subunit Ribosomal RNA	5.34	3.60E-05
BC0305	K01977 16S ribosomal RNA | (GenBank) Small Subunit Ribosomal RNA	5.33	7.50E-04
BC0085	K01977 16S ribosomal RNA | (GenBank) Small Subunit Ribosomal RNA	5.31	5.60E-05
BC0390	K06325 spore coat protein B | (GenBank) Spore coat protein B	5.29	4.20E-04
BC0521	K01977 16S ribosomal RNA | (GenBank) Small Subunit Ribosomal RNA	5.28	3.40E-06
BC0551	K09786 uncharacterized protein | (GenBank) Glycosyltransferase	5.26	5.40E-04
BC0029	K01977 16S ribosomal RNA | (GenBank) Small Subunit Ribosomal RNA	5.24	1.10E-04
BC0954	no KO assigned | (GenBank) tcdA-E operon negative regulator	5.05	4.30E-04
BC0332	K11175 phosphoribosylglycinamide formyltransferase 1 [EC:2.1.2.2] | (GenBank) Phosphoribosylglycinamide formyltransferase	4.82	1.30E-04
BC0096	K01977 16S ribosomal RNA | (GenBank) Small Subunit Ribosomal RNA	4.8	1.50E-03
BC0325	K01756 adenylosuccinate lyase [EC:4.3.2.2] | (GenBank) Adenylosuccinate lyase	4.75	8.50E-04
BC4207	no KO assigned | (GenBank) hypothetical protein	4.74	7.50E-04
BC0875	K06419 small acid-soluble spore protein B (major beta-type SASP) | (GenBank) Small acid-soluble spore protein	4.74	6.90E-04

BC5391	K06305 spore germination protein Q | (GenBank) hypothetical protein	4.53	4.30E-04
BC4495	K06298 germination protein M | (GenBank) Germination protein germ	4.51	1.80E-03
BC5052	no KO assigned | (GenBank) BC_5052	4.44	8.50E-04
BC5282	K06283 putative DeoR family transcriptional regulator, stage III sporulation protein D | (GenBank) Stage III sporulation protein D	4.36	4.50E-05
BC1212	K00973 glucose-1-phosphate thymidylyltransferase [EC:2.7.7.24] | (GenBank) Glucose-1-phosphate thymidylyltransferase	4.34	1.00E-03
BC0331	K01933 phosphoribosylformylglycinamidine cyclo-ligase [EC:6.3.3.1] | (GenBank) Phosphoribosylformylglycinamidine cyclo-ligase	4.17	4.50E-05
BC1383	K03327 multidrug resistance protein, MATE family | (GenBank) Na+ driven multidrug efflux pump	4.13	1.10E-03
BC3441	K07273 lysozyme | (GenBank) Lysozyme M1 precursor	4.11	2.00E-03
BC1862	no KO assigned | (GenBank) Phage protein	4.09	1.10E-03
BC0625	K04748 nitric oxide reductase NorQ protein | (GenBank) NorQ protein	3.76	4.60E-04
BC5232	K19005 lipoteichoic acid synthase [EC:2.7.8.20] | (GenBank) Phosphoglycerol transferase	3.71	1.30E-04
BC0288	K03789 [ribosomal protein S18]-alanine N-acetyltransferase [EC:2.3.1.266] | (GenBank) Ribosomal-protein-alanine acetyltransferase	3.7	1.50E-03
BC1043	K07533 foldase protein PrsA [EC:5.2.1.8] | (GenBank) Protein export protein prsA precursor	3.63	1.90E-05
BC5404	K00003 homoserine dehydrogenase [EC:1.1.1.3] | (GenBank) Homoserine dehydrogenase	3.46	3.50E-04
BC3867	K13038 phosphopantothenoylcysteine decarboxylase / phosphopantothenate---cysteine ligase [EC:4.1.1.3	3.46	4.80E-04
BC1671	K02392 flagellar basal-body rod protein FlgG | (GenBank) Flagellar basal-body rod protein flgG	3.46	1.20E-04
BC0593	K03294 basic amino acid/polyamine antiporter, APA family | (GenBank) Alanine permease	3.39	7.50E-04
BC1606	no KO assigned | (GenBank) hypothetical Cytosolic Protein	3.37	1.10E-03
BC3586	K15580 oligopeptide transport system substrate-binding protein | (GenBank) Oligopeptide-binding protein oppA	3.37	2.90E-04
BC0379	K08963 methylthioribose-1-phosphate isomerase [EC:5.3.1.23] | (GenBank) Methylthioribose salvage protein	3.19	5.80E-04
BC4212	no KO assigned | (GenBank) Transcriptional regulator, TetR family	3.16	1.70E-03
BC0323	K01588 5-(carboxyamino)imidazole ribonucleotide mutase [EC:5.4.99.18] | (GenBank)	3.15	1.30E-03

	Phosphoribosylaminoimidazole carboxylase catalytic subunit		
BC0380	K01628 L-fuculose-phosphate aldolase [EC:4.1.2.17] | (GenBank) L-fuculose phosphate aldolase	2.88	7.80E-04
BC1569	K03816 xanthine phosphoribosyltransferase [EC:2.4.2.22] | (GenBank) Xanthine phosphoribosyltransferase	2.8	8.20E-04
BC3896	K04074 cell division initiation protein | (GenBank) Cell division protein DIVIVA	2.8	4.20E-04
BC0301	K01980 23S ribosomal RNA | (GenBank) Large Subunit Ribosomal RNA	2.64	1.30E-03
BC3651	K01712 urocanate hydratase [EC:4.2.1.49] | (GenBank) Urocanate hydratase	-3.04	2.00E-03
BC3981	K05822 tetrahydrodipicolinate N-acetyltransferase [EC:2.3.1.89] | (GenBank) Tetrahydrodipicolinate N-acetyltransferase	-3.18	7.80E-05
BC4886	K14219 tRNA Arg | (GenBank) tRNA-Arg	-3.2	2.30E-04
BC0898	no KO assigned | (GenBank) 3-hydroxybutyryl-CoA dehydratase	-3.32	3.40E-04
BC2932	K19005 lipoteichoic acid synthase [EC:2.7.8.20] | (GenBank) Phosphoglycerol transferase	-3.47	1.50E-04
BC2527	K07052 CAAX protease family protein | (GenBank) CAAX amino terminal protease family	-3.51	6.70E-04
BC3264	no KO assigned | (GenBank) hypothetical protein	-3.56	4.00E-04
BC2699	no KO assigned | (GenBank) BC_2699	-3.56	9.50E-04
BC4020	no KO assigned | (GenBank) hypothetical protein	-3.61	1.00E-04
BC2725	K00675 N-hydroxyarylamine O-acetyltransferase [EC:2.3.1.118] | (GenBank) N-hydroxyarylamine O-acetyltransferase	-3.67	7.20E-04
BC4298	K01489 cytidine deaminase [EC:3.5.4.5] | (GenBank) Cytidine deaminase	-3.71	1.80E-05
BC5471	K02939 large subunit ribosomal protein L9 | (GenBank) LSU ribosomal protein L9P	-3.87	9.90E-05
BC1975	no KO assigned | (GenBank) hypothetical Exported Protein	-4.16	3.00E-04
BC5034	K03406 methyl-accepting chemotaxis protein | (GenBank) Methyl-accepting chemotaxis protein	-5.03	7.50E-04
BC5054	K07118 uncharacterized protein | (GenBank) putative NAD-dependent dehydrogenase	-6	1.40E-03
BC0820	K03311 branched-chain amino acid:cation transporter, LIVCS family | (GenBank) Branched-chain amino acid transport system carrier protein	-6.29	1.20E-03
BC3155	K03892 ArsR family transcriptional regulator, arsenate/arsenite/antimonite-responsive transcriptional regulator	-6.47	1.60E-03
BC1979	K24109 3,4-dihydroxybenzoyl-citryl-spermidine/N-citryl-spermidine--spermidine ligase [EC:6.3.2.-] | (GenBank) Siderophore biosynthesis protein	-7.31	4.40E-04

BC5241	no KO assigned | (GenBank) IG hypothetical 16680	-7.86	5.80E-07
BC4532	no KO assigned | (GenBank) hypothetical protein	-8.94	1.40E-04
BC4821	no KO assigned | (GenBank) hypothetical protein	-8.96	1.90E-03
BC0774	K01193 beta-fructofuranosidase [EC:3.2.1.26] | (GenBank) Sucrose-6-phosphate hydrolase	-9.41	8.10E-04
BC4361	K02013 iron complex transport system ATP-binding protein [EC:7.2.2.-] | (GenBank) Ferrichrome transport ATP-binding protein fhuC	-9.63	1.10E-04
BC5242	no KO assigned | (GenBank) Membrane protein with C2C2 zinc finger	-10.61	1.20E-06
BC3595	K16044 scyllo-inositol 2-dehydrogenase (NADP+) [EC:1.1.1.371] | (GenBank) Oxidoreductase	-11.03	5.20E-04
BC1898	no KO assigned | (GenBank) Phage protein	-11.28	3.00E-04
BC0579	K11616 malate:Na+ symporter | (GenBank) Malate-sodium symport	-11.37	6.80E-04
BC5066	no KO assigned | (GenBank) Endonuclease/Exonuclease/phosphatase family protein	-11.49	3.70E-07
BC2323	K02003 putative ABC transport system ATP-binding protein | (GenBank) ABC transporter ATP-binding protein	-11.65	1.20E-03
BC4999	K07052 CAAX protease family protein | (GenBank) CAAX amino terminal protease family	-11.83	1.20E-09
BC2584	no KO assigned | (GenBank) Phage protein	-11.92	3.30E-05
BC0428	no KO assigned | (GenBank) ABC transporter ATP-binding protein uup	-11.98	8.70E-07
BC0858	K03923 NADPH dehydrogenase (quinone) [EC:1.6.5.10] | (GenBank) Modulator of drug activity B	-12.05	1.30E-03
BC1856	no KO assigned | (GenBank) Phage protein	-12.11	3.60E-04
BC3527	no KO assigned | (GenBank) hypothetical protein	-12.12	3.00E-11
BC2413	no KO assigned | (GenBank) Phage protein	-12.12	1.50E-03
BC4744	no KO assigned | (GenBank) hypothetical Membrane Spanning Protein	-12.28	1.60E-04
BC0514	K25155 viologen exporter family transport system permease protein | (GenBank) Daunorubicin resistance transmembrane protein	-12.34	4.40E-04
BC1256	no KO assigned | (GenBank) hypothetical protein	-12.39	9.00E-04
BC2585	no KO assigned | (GenBank) Portal protein	-12.41	3.90E-04
BC0817	no KO assigned | (GenBank) hypothetical Membrane Spanning Protein	-12.48	1.70E-04
BC3423	no KO assigned | (GenBank) Transcriptional regulator, ArsR family	-12.6	1.80E-05
BC2690	no KO assigned | (GenBank) Quaternary ammonium compound-resistance protein	-12.71	5.10E-04
BC3436	K01990 ABC-2 type transport system ATP-binding protein | (GenBank) Daunorubicin resistance ATP-binding protein drrA	-12.8	1.50E-03

BC1663	K02417 flagellar motor switch protein FliN | (GenBank) Flagellar motor switch protein fliN	-12.85	2.10E-06
BC1104	no KO assigned | (GenBank) hypothetical protein	-12.86	5.40E-05
BC2093	no KO assigned | (GenBank) hypothetical protein	-12.99	6.60E-04
BC1860	no KO assigned | (GenBank) Phage protein	-13.27	1.10E-04
BC2135	K26138 nitrite reductase [NAD(P)H] small subunit [EC:1.7.1.4] | (GenBank) Nitrite reductase [NAD(P)H	-13.3	1.00E-04
BC3331	no KO assigned | (GenBank) hypothetical protein	-13.39	2.50E-04
BC1377	no KO assigned | (GenBank) hypothetical protein	-13.4	9.80E-09
BC1100	no KO assigned | (GenBank) hypothetical protein	-13.41	4.80E-05
BC3673	K07090 uncharacterized protein | (GenBank) hypothetical Membrane Spanning Protein	-13.69	1.10E-03
BC1857	K03547 DNA repair protein SbcD/Mre11 | (GenBank) Exonuclease SbcD	-13.85	4.90E-04
BC1901	no KO assigned | (GenBank) Phage protein	-13.87	3.50E-04
BC1874	no KO assigned | (GenBank) Phage protein	-13.89	7.90E-07
BC1321	no KO assigned | (GenBank) hypothetical protein	-14.01	1.20E-03
BC3225	no KO assigned | (GenBank) Macrolide-efflux protein	-14.01	5.70E-06
BC2085	no KO assigned | (GenBank) hypothetical protein	-14.13	4.40E-05
BC1103	no KO assigned | (GenBank) hypothetical protein	-14.18	1.90E-04
BC0587	no KO assigned | (GenBank) Acetyltransferase	-14.33	2.50E-05
BC0975	no KO assigned | (GenBank) Transcriptional regulator, MarR family	-14.47	7.70E-08
BC3877	no KO assigned | (GenBank) hypothetical Membrane Spanning Protein	-14.5	1.50E-04
BC1656	K02406 flagellin | (GenBank) Flagellin	-14.54	1.90E-07
BC3475	no KO assigned | (GenBank) hypothetical protein	-15.26	4.30E-04
BC1873	no KO assigned | (GenBank) Phage protein	-15.5	2.40E-05
BC2956	no KO assigned | (GenBank) hypothetical protein	-16.27	1.10E-10

Table S9.

Proteins differentially expressed in *ΔcalY* biofilm cells at 72 hours, identified by iTRAQ analysis. Proteins with a p-value < 0.05 were considered significantly differentially expressed.

Gene ID	Annotation	Log_2FC
BC4501	K01994 LuxR family transcriptional regulator, transcriptional regulator of spore coat protein | (GenBank) Germination protein gerE	1.227
BC0327	K23264 phosphoribosylformylglycinamidine synthase subunit PurS [EC:6.3.5.3] | (GenBank) Phosphorybosylformylglycinamidine synthetase, PurS component	1.177
BC1280	no KO assigned | (GenBank) hypothetical protein	1.095
BC0326	K01923 phosphoribosylaminoimidazole-succinocarboxamide synthase [EC:6.3.2.6] | (GenBank) Phosphoribosylamidoimidazole-succinocarboxamide synthase	1.071
BC0325	K01756 adenylosuccinate lyase [EC:4.3.2.2] | (GenBank) Adenylosuccinate lyase	1.014
BC0328	K23265 phosphoribosylformylglycinamidine synthase subunit PurQ / glutaminase [EC:6.3.5.3 3.5.1.2] | (GenBank) Phosphoribosylformylglycinamidine synthase	0.981
BC0331	K01933 phosphoribosylformylglycinamidine cyclo-ligase [EC:6.3.3.1] | (GenBank) Phosphoribosylformylglycinamidine cyclo-ligase	0.937
BC1569	K03816 xanthine phosphoribosyltransferase [EC:2.4.2.22] | (GenBank) Xanthine phosphoribosyltransferase	0.911
BC0333	K00602 phosphoribosylaminoimidazolecarboxamide formyltransferase / IMP cyclohydrolase [EC:2.1.2.3 3.5.4.10] | (GenBank) IMP cyclohydrolase	0.907
BC0324	K01589 5-(carboxyamino)imidazole ribonucleotide synthase [EC:6.3.4.18] | (GenBank) Phosphoribosylaminoimidazole carboxylase ATPase subunit	0.876
BC1779	K00053 ketol-acid reductoisomerase [EC:1.1.1.86] | (GenBank) Ketol-acid reductoisomerase	0.875
BC1780	K01687 dihydroxy-acid dehydratase [EC:4.2.1.9] | (GenBank) Dihydroxy-acid dehydratase	0.823
BC4074	K06378 stage II sporulation protein AA (anti-sigma F factor antagonist) | (GenBank) Anti-sigma F factor antagonist	0.807
BC0329	K23269 phosphoribosylformylglycinamidine synthase subunit PurL [EC:6.3.5.3] | (GenBank) Phosphoribosylformylglycinamidine synthase	0.807
BC2114	K00432 glutathione peroxidase [EC:1.11.1.9] | (GenBank) Glutathione peroxidase	0.776
BC3539	K03704 cold shock protein | (GenBank) Cold shock protein	0.757
BC0323	K01588 5-(carboxyamino)imidazole ribonucleotide mutase [EC:5.4.99.18] | (GenBank) Phosphoribosylaminoimidazole carboxylase catalytic subunit	0.715
BC0449	no KO assigned | (GenBank) hypothetical protein	0.701

BC4159	K00166 2-oxoisovalerate dehydrogenase E1 component subunit alpha [EC:1.2.4.4] | (GenBank) 2-oxoisovalerate dehydrogenase alpha subunit	-0.707
BC5081	no KO assigned | (GenBank) NADH oxidase (NOXASE)	-0.728
BC1651	K02390 flagellar hook protein FlgE | (GenBank) Flagellar hook protein flgE	-0.729
BC0666	K09607 immune inhibitor A [EC:3.4.24.-] | (GenBank) Immune inhibitor A precursor	-0.753
BC0668	K00004 (R,R)-butanediol dehydrogenase / meso-butanediol dehydrogenase / diacetyl reductase [EC:1.1.1.4 1.1.1.- 1.1.1.303] | (GenBank) (R,R)-butanediol dehydrogenase	-0.771
BC2392	no KO assigned | (GenBank) FenI	-0.818
BC4162	K00263 leucine dehydrogenase [EC:1.4.1.9] | (GenBank) Leucine dehydrogenase	-0.831
BC4025	no KO assigned | (GenBank) hypothetical protein	-0.844
BC0387	no KO assigned | (GenBank) hypothetical protein	-0.872
BC2849	K20742 gamma-D-glutamyl-L-lysine dipeptidyl-peptidase [EC:3.4.14.13] | (GenBank) Cell wall-associated hydrolase	-1.112
BC2984	K09607 immune inhibitor A [EC:3.4.24.-] | (GenBank) Immune inhibitor A precursor	-1.126
BC4696	K03439 tRNA (guanine-N7-)-methyltransferase [EC:2.1.1.33] | (GenBank) SAM-dependent methyltransferase	-1.2
BC3121	K11751 5'-nucleotidase / UDP-sugar diphosphatase [EC:3.1.3.5 3.6.1.45] | (GenBank) 5'-nucleotidase	-1.402
BC0615	K03305 proton-dependent oligopeptide transporter, POT family | (GenBank) Di-/tripeptide transporter	-1.556
BC2273	no KO assigned | (GenBank) hypothetical protein	-1.987
BC2861	K01281 X-Pro dipeptidyl-peptidase [EC:3.4.14.11] | (GenBank) Xaa-Pro dipeptidyl-peptidase	-2
BC4100	K01081 5'-nucleotidase [EC:3.1.3.5] | (GenBank) 5'-nucleotidase	-2.122
BC3133	K06889 uncharacterized protein | (GenBank) putative hydrolase	-2.257
BC1284	K09607 immune inhibitor A [EC:3.4.24.-] | (GenBank) Immune inhibitor A precursor	-2.27
BC1281	no KO assigned | (GenBank) Cell envelope-bound metalloprotease (camelysin)	-2.377
BC5359	K19701 aminopeptidase YwaD [EC:3.4.11.6 3.4.11.10] | (GenBank) Aminopeptidase Y	-2.684
BC0802	K19745 acrylyl-CoA reductase (NADPH) [EC:1.3.1.-] | (GenBank) Alcohol dehydrogenase	-2.736
BC5289	no KO assigned | (GenBank) hypothetical protein	-2.738

Table S10.

Biofilm formation of WT and *ΔcapP* strains. Biofilm mass was measured by crystal violet staining at different time points, quantified as absorbance at 595 nm. Values from all individual measurements of three independent experiments, each with three technical replicates, are reported.

24 hours	Experiment 1			Experiment 2			Experiment 3		
WT	0.53	0.57	0.88	0.90	1.10	2.21	2.68	1.18	0.81
ΔcapP	1.02	0.82	0.63	1.37	0.61	0.60	0.47	0.54	2.47
ΔcapP (sacA::capP)	0.48	1.04	1.74	2.46	2.38	2.93	2.32	1.53	0.98
ΔC-domain	0.62	0.78	0.72	2.63	3.41	1.93	0.85	0.77	0.93
48 hours	Experiment 1			Experiment 2			Experiment 3		
WT	8.60	4.84	5.96	2.60	7.03	6.44	7.88	6.72	7.46
ΔcapP	1.96	1.45	0.93	0.89	2.55	2.25	1.68	1.55	1.81
ΔcapP (sacA::capP)	3.63	3.78	5.91	4.61	5.44	4.03	4.20	3.57	4.52
ΔC-domain	7.66	3.74	3.78	2.89	4.57	7.14	4.21	4.82	5.69
72 hours	Experiment 1			Experiment 2			Experiment 3		
WT	29.32	31.16	29.20	37.00	19.76	25.64	24.08	22.04	30.02
ΔcapP	0.75	4.64	3.16	4.17	3.38	2.75	4.70	4.67	4.68
ΔcapP (sacA::capP)	21.80	37.56	30.24	49.44	42.16	46.00	30.72	30.84	33.45
ΔC-domain	23.48	22.68	14.84	25.41	17.00	18.36	43.96	35.72	27.23

Table S11.

Differentially expressed genes in the *ΔcapP* strain compared to the wild-type strain at 24 hours. Genes with a $\log_2$(Fold Change) value >1 or <-1, and a p-value < 0.05, were considered as differentially expressed.

Gene ID	Annotation	Log_2FC	p-value
BC2306	Glycine-AMP ligase (EC 2.7.7.-)	2.14	7.04E-113
BC2305	Isochorismatase (EC 3.3.2.1)	1.88	4.88E-49
BC5449	Conserved membrane protein (Hemolysin III-like protein)	1.75	6.50E-29
BC3734	IroE protein	1.71	1.17E-43
BC4545	Ferrichrome transport system permease protein fhuB	1.70	2.34E-14
BC0211	Oligopeptide-binding protein oppA	1.67	3.46E-45
BC2307	Glycine-AMP ligase (EC 2.7.7.-)	1.60	1.43E-37
BC1982	Uncharacterized protein	1.57	2.72E-34
BC1155	Catalase (EC 1.11.1.6)	1.51	3.09E-83
BC4546	High-affinity heme uptake system protein IsdE (Iron-regulated surface determinant protein E) (Staphylococcal iron-regulated protein F)	1.44	2.65E-11
BC3735	Iron(III) dicitrate transport ATP-binding protein fecE	1.41	7.59E-32
BC1941	Transport ATP-binding protein cydC	1.40	3.33E-30
BC2309	Antibiotic/siderophore biosynthesis protein	1.38	2.20E-05
BC1983	Hypothetical Cytosolic Protein	1.38	4.93E-22
BC1981	Acyl carrier protein	1.35	2.21E-16
BC2308	Glycine-AMP ligase (EC 2.7.7.-)	1.32	6.43E-10
BC2310	Multidrug resistance protein B	1.31	7.48E-15
BC3572	Uncharacterized protein	1.28	2.44E-19
BC1980	AMP-(Fatty)acid ligases	1.27	8.87E-26
BC3570	Sulfur carrier protein FdhD	1.26	1.05E-22
BC1436	Phage shock protein A	1.25	1.95E-03
BC2304	2 3-dihydroxybenzoate-AMP ligase (EC 2.7.7.58)	1.23	2.02E-30
BC5210	PTS system lichenan oligosaccharide-specific IIA component (EC 2.7.1.69)	1.21	1.39E-05
BC4544	Ferrichrome transport ATP-binding protein fhuC	1.19	5.51E-08
BC0808	PTS system diacetylchitobiose-specific IIB component (EC 2.7.1.69)	1.18	1.11E-08
BC3571	Hypothetical Cytosolic Protein	1.15	2.75E-16
BC5380	Ferrichrome-binding protein	1.13	4.77E-38
BC3670	Murein hydrolase export regulator	1.12	6.48E-04
BC3667	ABC transporter permease protein	1.11	5.10E-02
BC3565	Molybdopterin (MPT) converting factor subunit 2	1.10	4.89E-03
BC5385	K06314 prespore-specific regulator	1.08	2.51E-03
BC3736	Iron(III) dicitrate transport system permease protein fecC	1.04	4.97E-22
BC0210	Oligopeptide transport ATP-binding protein oppF	1.03	7.66E-17
BC1154	Coproporphyrin III ferrochelatase 2 (EC 4.99.1.9)	1.03	3.59E-36

Gene ID	Annotation	Log_2FC	p-value
BC1036	Glycerol-3-phosphate dehydrogenase (EC 1.1.5.3)	1.01	2.70E-20
BC4126	Ornithine carbamoyltransferase (OTCase) (EC 2.1.3.3)	-1.00	8.85E-19
BC1396	Branched-chain-amino-acid aminotransferase (BCAT) (EC 2.6.1.42)	-1.10	2.57E-03
BC1209	O-antigen biosynthesis protein rfbC	-1.10	6.40E-03
BC0768	Methylthioribose-binding protein	-1.17	3.72E-05

Table S12.

Differentially expressed genes in the *ΔcapP* strain compared to the wild-type strain at 48 hours. Genes with a $\log_2$(Fold Change) value >1 or <-1, and a p-value < 0.05, were considered as differentially expressed.

Gene ID	Annotation	Log_2FC	p-values
BC0851	Mercuric resistance operon regulatory protein	1.49	7.49E-04
BC0472	Uncharacterized protein	1.49	3.23E-12
BC4814	Uncharacterized protein	1.48	1.04E-19
BC3715	Uncharacterized protein	1.37	1.27E-02
BC2053	Phosphohydrolase (MutT/nudix family protein)	1.34	3.77E-05
BC2011	Non-specific DNA-binding protein Dps / Iron-binding ferritin-like antioxidant protein / Ferroxidase (EC 1.16.3.1)	1.22	5.43E-20
BC5439	Antiholin-like protein LrgA	1.21	7.62E-14
BC2856	Ankyrin repeat protein	1.21	6.33E-03
BC5438	Antiholin-like protein LrgB	1.19	1.29E-17
BC2307	Glycine-AMP ligase (EC 2.7.7.-)	1.09	1.19E-12
BC1844	Nitroreductase family protein	1.09	2.91E-08
BC1133	Uncharacterized protein	1.08	2.99E-04
BC0983	Uncharacterized protein	1.08	3.28E-02
BC2969	ABM domain-containing protein	1.06	2.01E-12
BC5445	Superoxide dismutase [Mn] 2 (EC 1.15.1.1)	1.06	6.20E-21
BC5087	Putative lantibiotic precursor peptide	1.06	9.91E-13
BC1689	Uncharacterized protein	1.04	2.35E-06
BC4242	L-cystine uptake protein TcyP (Transporter of cystine TcyP)	1.03	6.49E-09
BC2300	Oxalate/formate antiporter	1.02	8.32E-12
BC2385	Dehydrogenase	1.02	3.43E-02
BC1436	Phage shock protein A	1.01	5.49E-16
BC0346	Methionine import ATP-binding protein MetN 2 (EC 7.4.2.11)	-1.01	2.44E-02
BC1760	3-oxoacyl-[acyl-carrier-protein] synthase 3 protein 2 (EC 2.3.1.180) (3-oxoacyl-[acyl-carrier-protein] synthase III protein 2) (Beta-ketoacyl-ACP synthase III 2) (KAS III 2)	-1.01	8.95E-10
BC0233	Uncharacterized protein	-1.01	3.00E-02
BC2606	Hypothetical Membrane Spanning Protein	-1.05	1.74E-03
BC0710	Phosphate-binding protein	-1.07	6.16E-05
BC3007	Acetylornithine aminotransferase (EC 2.6.1.11)	-1.09	5.75E-04
BC3890	Uracil permease	-1.11	7.16E-10
BC0363	Nucleoside permease nupC	-1.12	2.35E-02
BC1954	Esterase (EC 3.1.1.-)	-1.13	4.03E-15
BC2771	Hypothetical Exported Protein	-1.14	1.68E-01
BC4802	Uncharacterized protein	-1.19	2.00E-10
BC0711	Phosphate transport system permease protein	-1.22	7.00E-04

BC2796	Ribose-5-phosphate isomerase A (EC 5.3.1.6) (Phosphoriboisomerase A) (PRI)	-1.23	2.76E-01
BC3030	SMI1_KNR4 domain-containing protein	-1.23	1.26E-01
BC3319	Uncharacterized protein	-1.23	1.17E-02
BC5027	Protein erfK/srfK	-1.29	2.32E-13
BC0768	Methylthioribose-binding protein	-1.37	2.95E-03
BC1422	Sulfate adenylyltransferase (EC 2.7.7.4) (ATP-sulfurylase) (Sulfate adenylate transferase) (SAT)	-1.37	4.40E-03
BC1424	Ferredoxin--nitrite reductase (EC 1.7.7.1)	-1.47	6.28E-09
BC1423	Adenylyl-sulfate kinase (EC 2.7.1.25) (APS kinase) (ATP adenosine-5'-phosphosulfate 3'-phosphotransferase) (Adenosine-5'-phosphosulfate kinase)	-1.47	4.64E-04
BC2799	Macrolide glycosyltransferase (EC 2.4.1.-)	-1.57	9.23E-02
BC1060	Collagen adhesion protein	-1.58	2.99E-10
BC3031	Isoflavone reductase	-1.62	2.83E-02
BC5026	Uncharacterized protein	-2.02	4.94E-10
BC1281	Cell envelope-bound metalloprotease (Camelysin) (EC 3.4.24.-)	-3.14	2.16E-38
BC1279	Spore coat-associated protein N	-3.23	6.35E-05
BC2793	ATP-dependent Clp protease proteolytic subunit 1 (EC 3.4.21.92) (Endopeptidase Clp 1)	-8.11	1.39E-03
BC2794	RNA polymerase ECF-type sigma factor	-8.43	6.24E-04

Table S13.

Individual measurements of biofilm biomass after 72 hours in *ΔcapP* strains overexpressing *bc2793-bc2794* or *bc2794*. Biofilms were stained with crystal violet and biomass was quantified by absorbance at 575 nm. Individual measurements from three biological replicates, each with three technical replicates, are shown.

ΔcapP (pUTE675-*bc2793,bc2794*)									
IPTG (μM)	Experiment 1			Experiment 2			Experiment 3		
0	0.51	1.25	0.92	1.05	0.86	0.60	0.51	0.95	1.57
5	0.85	0.79	0.73	1.09	0.74	0.81	0.74	1.11	0.76
10	1.38	3.00	2.14	2.01	1.48	1.42	3.01	2.15	1.52
ΔcapP (pUTE675-*bc2794*)									
IPTG (μM)	Experiment 1			Experiment 2			Experiment 3		
0	1.368	1.75	1.91	1.26	1.32	1.122	0.734	1.108	1.272
10	6.16	4.66	2.27	1.252	2.176	0.814	0.85	0.642	0.914
100	11.44	12.26	4.02	11.74	6.4	4.04	4.54	5.7	7.45

Table S14.

Biofilm formation at 72 hours of *Δbc2793-bc2794* and *Δbc2794* deletion mutants compared to *B. cereus* ATCC14579 wild-type and *ΔcapP* strains. Biofilms were stained with crystal violet, and surface-adhered biomass was quantified by measuring absorbance at 575 nm. Individual measurements from three biological replicates, each with three technical replicates, are shown.

	Experiment 1			Experiment 2			Experiment 3		
WT	29.32	27.86	29.20	35.12	23.15	25.87	21.65	27.42	31.16
ΔcapP	1.68	2.65	1.72	2.16	1.85	2.59	1.91	2.36	2.48
Δbc2793-bc2794	15.88	16.90	18.36	16.94	14.74	16.28	15.9	22.06	15.42
Δbc2794	25.39	22.64	25.92	31.16	20.86	28.18	27.66	28.24	18.44

Table S15.

Biofilm formation of *B. cereus* ATCC14579 wild-type and *ΔcapP* strains after overexpression of *tasA*, *calY* or both using the pUTE657 plasmid and induction with 10 µM IPTG. Strains carrying the plasmid grown without IPTG were included as negative controls. Biofilms were incubated for 72 hours, stained with crystal violet and biomass quantified by absorbance at 575 nm. Values represent individual measurements from three biological experiments with ≥ 2 technical replicates.

		Experiment 1			Experiment 2			Experiment 3	
WT		25.50	22.92	27.06	21.20	30.34	14.76	22.70	24.24
WT + pUTE675 (*tasA*)	No IPTG	31.92	35.38	32.50	32.66	28.58	34.74	24.94	31.24
	10 µM IPTG	29.24	26.72	22.36	31.74	20.44	15.76	16.50	25.06
WT + pUTE675 (*calY*)	No IPTG	30.84	34.05	31.89	32.47	28.46	33.87	25.12	30.76
	10 µM IPTG	21.02	24.23	30.73	20.77	27.31	21.17	22.21	29.47
WT + pUTE675 (*tasA,calY*)	No IPTG	17.58	13.40	26.10	17.66	15.48	15.84	7.90	14.44
	10 µM IPTG	12.62	24.14	13.24	13.12	9.36	4.24	5.14	8.48
ΔcapP		0.62	0.67	0.45	0.58	0.83	0.58	0.32	0.32
ΔcapP + pUTE675 (*tasA*)	No IPTG	0.76	0.31	0.40	0.35	0.23	0.28	0.24	0.40
	10 µM IPTG	1.16	0.90	0.72	1.09	0.18	0.35	0.19	0.53
ΔcapP + pUTE675 (*calY*)	No IPTG	1.61	0.76	1.48	0.85	0.29	0.52	0.39	0.28
	10 µM IPTG	0.41	0.65	2.50	1.17	1.34	0.77	0.88	0.71
ΔcapP + pUTE675 (*tasA,calY*)	No IPTG	0.33	0.27	0.17	0.13	0.31	0.15	0.18	0.42
	10 µM IPTG	0.37	0.16	0.14	0.23	0.21	0.17	0.16	0.18

Table S16.

Biofilm biomass measurements from extracellular complementation assays of the *ΔcapP* mutant with 6 µM TasA or CalY (monomeric or polymerized). The negative control corresponds to *in vitro*-produced protein added to TY medium. Biofilm biomass was assessed by crystal violet staining and quantified by absorbance at 575 nm. Values represent three independent experiments, each comprising three technical replicates.

	Experiment 1			Experiment 2			Experiment 3		
ΔcapP	1.95	2.00	1.50	2.90	1.87	2.15	1.65	2.67	2.08
ΔcapP + TasA monomer	1.85	1.55	1.09	1.78	1.72	1.42	1.10	1.52	1.50
ΔcapP + TasA aggregates	4.20	5.80	6.30	4.90	5.10	6.50	4.60	5.90	5.42
ΔcapP + CalY monomer	1.30	3.34	3.16	1.98	1.94	2.10	2.85	1.60	2.29
ΔcapP + CalY aggregates	5.10	7.20	6.50	4.80	7.60	5.90	6.80	6.30	6.27
Empty well + TasA monomer	0.17	0.19	0.18	0.19	0.17	0.16	0.18	0.17	0.16
Empty well + TasA aggregates	0.23	0.24	0.27	0.26	0.24	0.24	0.26	0.27	0.30
Empty well + CalY monomer	0.18	0.18	0.19	0.19	0.18	0.18	0.19	0.18	0.18
Empty well + CalY aggregates	0.43	0.44	0.43	0.44	0.46	0.43	0.44	0.46	0.43

Table S17.

Individual measurements of biofilm formation in the *B. cereus* ATCC14579 wild-type strain overexpressing full-length *capP-His* or the *N-domain*$_{39\text{-}190}$*-His* from the pUTE657 plasmid, induced with 10 or 100 µM IPTG. Biofilms were stained with crystal violet after 72 hours, and biomass was quantified by absorbance 575 nm. Data represent three biological experiments, each with at least two technical replicates.

B. cereus ATCC14579 WT		Experiment 1			Experiment 2			Experiment 3	
+ pUTE675 (*capP-His*)	No IPTG	62.55	52.81	53.10	46.12	46.17	50.61	52.50	36.00
	10 µM IPTG	48.72	31.39	44.79	52.79	36.46	38.39	30.13	39.52
	100 µM IPTG	14.52	50.80	26.64	41.88	36.60	36.72	33.72	33.72
+ pUTE675 (*N-domain*$_{39\text{-}190}$*-His*)	No IPTG	62.50	52.89	53.10	46.13	46.19	50.67	52.50	36.05
	10 µM IPTG	48.72	31.39	44.79	52.79	36.46	38.39	30.13	39.52
	100 µM IPTG	14.52	50.80	33.79	41.88	36.60	36.72	33.72	26.64

Table S18.

Individual measurements of biofilm formation in the *B. cereus* ATCC14579 strain *ΔcapP* overexpressing full-length *capP-His* or the *N-domain*$_{39\text{-}190}$*-His* from the pUTE657 plasmid, induced with 10 or 100 µM IPTG. Biofilms were stained with crystal violet after 72 hours, and biomass was quantified by absorbance 575 nm. Data represent three biological experiments, each with three technical replicates.

B. cereus ATCC14579 *ΔcapP*		Experiment 1			Experiment 2			Experiment 3		
+pUTE675 (*capP-His*)	No IPTG	72.39	71.92	75.72	70.92	71.92	59.99	56.79	53.59	71.80
	10 µM IPTG	28.00	35.84	39.36	22.08	40.92	24.60	21.32	39.12	30.02
	100 µM IPTG	19.88	28.48	37.68	50.96	38.36	17.40	15.68	30.32	30.50
+pUTE675 (*N-domain*$_{39\text{-}190}$*-His*)	No IPTG	22.83	16.00	20.47	29.12	25.28	24.09	24.23	21.05	19.67
	10 µM IPTG	3.88	6.00	9.08	14.04	10.63	13.80	15.48	14.32	7.92
	100 µM IPTG	1.62	1.17	1.39	3.44	1.58	1.01	1.29	0.63	1.43

Table S19.

Quantitative DLS measurements of SEC elution peaks for CapP-His and N-domain$_{39-190}$-His.

	SEC elution	Signals detected by DLS	Size (nm)	Intensity	Z-average (d. nm)	PDI
CapP-His	1	Peak 1	152 ±84.18	98%	117.7	0.244
		Peak 2	4786 ± 730.9	1.2%		
	2	Peak 1	9.071 ± 2.446	17.6%	59.89	1.00
		Peak 2	333.3 ± 259.7	81.4%		
		Peak 3	4629 ± 807.1	1%		
N-domain$_{39-190}$-His	1	Peak 1	0.81 ± 0.17	11.7%	188.8	0.539
		Peak 2	388.7 ± 290.9	86.2%		
	2	Peak 1	7.605 ± 2.372	53.1%	93.48	0.174
		Peak 2	181 ± 72.70	37.8%		
		Peak 3	4862 ± 697.8	9.1%		

Table S20.

Secondary-structure content of CalY determined by DichroWeb analysis. CalY (40 µM) alone or with CapP-His (2 or 10 µM) was incubated for 16 hours at 37 °C under agitation (200 rpm). CD spectra were averaged over six scans after blank correction. CapP-His contributions were substracted and corrected spectra were analyzed using DichroWeb (53) (CDSSTR and CONTIN, reference set 7, 190-240 nm). Values represent the average of both algorithms.

	Helix	Strand	Turns	Unordered
CalY 40 µM	0.08	0.28	0.18	0.46
CalY 40 µM in presence of 2 µM CapP	0.15	0.29	0.18	0.46
CalY 40 µM in presence of 10 µM CapP	0.05	0.36	0.22	0.36

Table S21.
Bacterial strains used in this study.

Bacterial strain	Source	Identifier
B. cereus ATCC14579 WT	Spanish Collection of Type strains	ATCC14579
B. cereus ATCC14579 *ΔtasA*	Caro-Astorga, J. et al. 2015 (30)	N/A
B. cereus ATCC14579 *ΔcalY*	Caro-Astorga, J. et al. 2015 (30)	N/A
B. cereus ATCC14579 *Δflag*	Caro-Astorga, J. et al. 2020 (76)	N/A
B. cereus ATCC14579 *ΔtasA,flag*	This paper	N/A
B. cereus ATCC14579 *ΔcapP*	This paper	N/A
B. cereus ATCC14579 *ΔcapP (sacA::bc1280)*	This paper	N/A
B. cereus ATCC14579 *ΔC-domain*	This paper	N/A
B. cereus ATCC14579 *Δbc2794*	This paper	N/A
B. cereus ATCC14579 *Δbc2793-bc2794*	This paper	N/A
B. cereus ATCC14579 *ΔcapP* (pUTE657 – *tasA,* Spc^{+})	This paper	N/A
B. cereus ATCC14579 *ΔcapP* (pUTE657 – *calY,* Spc^{+})	This paper	N/A
B. cereus ATCC14579 *ΔcapP* (pUTE657 - *bc2793,bc2794,* Spc^{+})	This paper	N/A
B. cereus ATCC14579 *ΔcapP* (pUTE657- *bc2794,* Spc^{+})	This paper	N/A
B. cereus ATCC14579 *ΔcapP* (pUTE657 - *bc1280*-6His, Spc^{+})	This paper	N/A
B. cereus ATCC14579 *ΔcapP* (pUTE657 – *Ndomain*-6His, Spc^{+})	This paper	N/A
B. cereus AH187 (F4810/72) WT	Bacillus Genetic Stock Center	AH187 (F4810/72)
B. cereus AH187 (F4810/72) *ΔcapP*	This paper	N/A
B. cereus AH187 (F4810/72) *ΔtasA*	This paper	N/A
B. cereus AH187 (F4810/72) *ΔcalY*	This paper	N/A

Table S22

Primers used in this study for plasmid construction.

Name	Sequence
Flag.up.fw	cgatgcatgccatggtacccttattttgaaacgtcgatatttaatattttttaatatatc
Flag.up.rv	aacacagtaagtgcttgcatataagaaag
Flag.dw.fw	atgcaagcacttactgtgttacggtcataac
Flag.dw.rv	cttctagaattcgagctcccgtggcacagattagtatc
Up.1280.14579 Fw	tctatcgatgcatgccatggtacccattactaataaaaagactgtgtaaaattttg
Up.1280.14579 Rv	ttcactaaagcagcgaacattacgttctttatac
Down.1280.14579 Fw	aacgtaatgttcgctgctttagtgaaacagc
Down.1280.14579 Rv	agaagcttctagaattcgagctcccttgtacaaagataagacagac
mut1280 Fw	catcagcagtattaggagcagc
mut1280 Rv	caatgctgctgatgcaactc
Up.C-term Fw	tctatcgatgcatgccatggtaccccgatgaaattaatatggaagataattagc
Up.C-term Rv	actaaagcagttacgcttcaattgcaacgtttaattg
Down.C-term Fw	tgcaattgaagcgtaactgctttagtgaaacag
Down.C-term Rv	agaagcttctagaattcgagctccccccctaataaataatatgtattacaaattg
mutC-term Fw	gcgtgagtatggaacgatgaagg
mutC-term Rv	cctagactccgaatggacac
SacA UP_fwd	tctatcgatgcatgccatggtacccatggatatgaaacaaattgcaactc
SacA UP_rev	cagatttacctgcttatttactttctttatataaaatgaatgtgg
1280_fwd	gaaagtaaataagcaggtaaatctgataaattcaaag
1280_rev	agaggaatgtaacccctagcttttttttatatttttatc
SacA Down_fwd	aaaaagctaggggttacattcctctccaaaaatcatgc
SacA Down_rev	agaagcttctagaattcgagctccctcatttaatattagtaaatcttacgacg
Up.1280.AH187 Fw	tctatcgatgcatgccatggtacccattactagtttacaaggactg
Up.1280.AH187 Rv	tgtattttacttgtttaatctctcctatcgaac
Down.1280.AH187 Fw	aggagagattaaacaagtaaaatacagcgacatatag
Down.1280.AH187 Rv	agaagcttctagaattcgagctcccgtttgttctttttagcgaag
Up.tasA.AH187 Fw	aaaaggatccggctatgattacgagaggtg
Up.tasA.AH187 Rv	cctatcacctcaaatggttcgctgtaaagtcatgtccatatccccttaata
Down.tasA.AH187 Fw	cgagcgcctacgaggaatttgtatcggcgataaacttca attaaaatggacatttg
Down.tasA.AH187 Rv	aaaaccatggtttctttactttatcccctg
Up.calY.AH187 Fw	cgatgcatgccatggtacccatgctgaagcaaccgcgtaaattcaaaaag
Up.calY.AH187 Rv	gctttttggtaatcaattccccctagctgtttttttatatttttttattg
Down.calY.AH187 Fw	ggaattgattaccaaaaagcggggatttcccgctttttttataaag
Down.calY.AH187 Rv	cttctagaattcgagctcccccccttatttcctcccctaataaataatatgtattacaaattgaatt ttattaaaaattc
Up.2794Fw	tctatcgatgcatgccatggtaccctaaaaaggctagagaattaacatatcc
Up.2794Rv	taattttggcaacaagtattggaggaatttaaaatgaatac
Down.2794Fw	cctccaatacttgttgccaaaattaacaattgaaggtg
Down.2794Rv	agaagcttctagaattcgagctcccttacaatttctttatcccatttttagtac
2794Fw	cgctacgacactactcgctac
2794Rv	gttagcagcatattcgccagc

Name	Sequence
Up.2793-94Fw	cttctagaattcgagctcccccttatttcctcccctaataaataatatgtattacaaattgaatt ttattaaaaattc
Up.2793-94Rv	attttggcaattaatcctttacccattcatcaactaatg
Down.2793-94Fw	aaaggattaattgccaaaattaacaattgaaggtg
Down.2793-94Rv	cttctagaattcgagctcccttacaatttctttatcccatttttagtac
2793-94Fw	tcgctgttacgctttggaatc
2793-94Rv	gttagcagcatattcgccagc
657-1280HisFw	aaaaagtcgacagagaacaaggagggatgggagagattaag
657-1280HisRv	aaaaagcatgcttagtgatggtgatggtgatgctccgctttcttctgctcttctactttttc
657-NdomHisFw	aaaaagtcgacagagaacaaggagggatgggagagattaag
657-NdomHisRv	aaaaagcatgcttagtgatggtgatggtgatgtccttccgcttcaattgcaacgtttaattgtt gc
657-tasAFw	aaaaaagtcgacagagaacaaggagggatggacatgactttaaagaaaaaattagg
657-tasARv	aaaaagcatgcctatttttcttcacctgctgtttgttgtgc
657-calYFw	aaaaaagtcgacagagaacaaggagggttgattgtgagtctgaaaaagaaattagg
657-calYRv	aaaaagcatgcttatttttcttccccagcttcttggttggc
657-*tasA,calY*Up.Fw	gaattagcttgcatgcggctagctgagagaacaaggagggatggacatgac
657-*tasA,calY*Up.Rv	actcacaatcaactatttttcttcacctgctgtttgttg
657-*tasA,calY*Down.Fw	gaagaaaaatagttgattgtgagtctgaaaaagaaattag
657-*tasA,calY*Down.Rv	gtgagcggataacaattaagcttagttatttttcttccccagcttcttgg
657-2794Fw	agcttgcatgcggctagctgagagaacaaggagggatgtgcacaaaag
657-2794Rv	cggataacaattaagcttagttatgctgcgcaaagcatacaactg
657-2793,94Up.Fw	gaattagcttgcatgcggctagctgagagaacaaggagggatgaatacaattc
657-2793,94Up.Rv	tgcacatttttttttatttttcgtaacaacgtcatctac
657-2793,94Down.Fw	acgaaaaaataaaaaaaaaatgtgcacaaaagtaactcatg
657-2793,94Down.Rv	gtgagcggataacaattaagcttagttatgctgcgcaaagcatacaactg
NdeI.Fw CapP	aaaaaacatatggcgttcattcatgaaacgaaagtagaagcg
XhoI.Rv CapP	aaaaaactcgagctccgctttcttctgctcttctac
NdeI.Fw Ndom	aaaaaacatatggcgttcattcatgaaacgaaagtagaagcg
XhoI.Rv Ndom	aaaaaactcgagagaaccgcgtggcaccagtccttccgcttcaattgcaacg
NdeI.Fw Cdom	aaaaaacatatgcaaaagaaagtagaagaacaaaagaaagca

Table S23.
Primers used for RT-qPCR experiments.

Gene	Primer	Sequence	Amplicon
rpoA	Fw	TTTAACTGCAAAGCGTGGCC	153 bp
	Rv	TTAGCCACTTGTCCGACACG	
bc1280	Fw	ACAGAAGCGGCGTTCATTCA	200 bp
	Rv	TGTTGCCATACAGAAATCGCC	
tasA	Fw	ATCGGCTTGGTTCTGGGATG	158 bp
	Rv	CACCTGCTGTTTGTTGTGCA	
calY	Fw	CCTGAGTGGGGAGAAAAGGG	151 bp
	Rv	CAGCTTCTTGGTTGGCATTGA	
bc1583	Fw	CGGTGTGATTTGGTCACTTG	156 bp
eps2	Rv	CCCGAGCGAAACAAAAATAA	
bc5263	Fw	ATCGTTGATGCAGTTCGTGA	200 bp
eps1	Rv	GCATATCCATTCGGTTGCTT	
bc5268	Fw	TGGCTGGTCAACTGTCTGAG	198 bp
eps1	Rv	CCCAGCCGTTCAAATATCTC	
bc5277	Fw	TGTAACGGATGCGCAAATTA	160 bp
eps1	Rv	TTCACGTTCACGGTCGTTTA	
bc5279	Fw	TTAATGGCAGCAGGTCCAAT	155 bp
eps1	Rv	TTCGCAAGAATTTGTGCATC	

Table S24.
Primers used for the determination of the transcriptional unit in the *sipW*-to-*calY* genomic region.

Name	Sequence	Amplicon	Purpose
tasA-capP Fw	ATCGGCTTGGTTCTGGGATG	974 bp	Amplification of the intergenic region between *tasA* and *capP*.
tasA-capP Rv	TTTCTCACGCCCTTGTTGCC		
capP Fw	CGATGGGAGAGATTAAGATGCTG	572 bp	Amplification of *capP*.
capP Rv	TGTCCTTCCGCTTCAATTGC		
capP - calY Fw	GTAGAAGCGACGCTTTCTAC	1668 bp	Amplification of the intergenic region between *capP* and *calY*.
capP - calY Rv	GCTGATGCAACTCCCATACC		
calY Fw	AGGTATGGGAGTTGCATCAG	560 bp	Amplification of *calY*.
calY Rv	CAGCTTCTTGGTTGGCATTG		
